# $\Delta I=1/2$ rule, $\varepsilon '/\varepsilon$ and $K\rightarrow \pi \nu \bar{\nu }$ in $Z^\prime (Z)$ and $G^\prime$ models with FCNC quark couplings

**DOI:** 10.1140/epjc/s10052-014-2950-z

**Published:** 2014-07-11

**Authors:** Andrzej J. Buras, Fulvia De Fazio, Jennifer Girrbach

**Affiliations:** 1 TUM Institute for Advanced Study, Lichtenbergstr. 2a, 85747 Garching, Germany; 2 Physik Department, Technische Universität München, James-Franck-Straße, 85747 Garching, Germany; 3 Istituto Nazionale di Fisica Nucleare, Sezione di Bari, Via Orabona 4, 70126 Bari, Italy

## Abstract

The experimental value for the isospin amplitude $\mathrm{Re}A_2$ in $K\rightarrow \pi \pi$ decays has been successfully explained within the standard model (SM), both within the large $N$ approach to QCD and by QCD lattice calculations. On the other hand within the large $N$ approach the value of $\mathrm{Re}A_0$ is by at least $30\,\%$ below the data. While this deficit could be the result of theoretical uncertainties in this approach and could be removed by future precise QCD lattice calculations, it cannot be excluded that the missing piece in $\mathrm{Re}A_0$ comes from new physics (NP). We demonstrate that this deficit can be significantly softened by tree-level FCNC transitions mediated by a heavy colourless $Z^\prime$ gauge boson with a flavour-violating *left-handed* coupling $\Delta ^{sd}_L(Z^\prime )$ and an approximately universal flavour diagonal *right-handed* coupling $\Delta ^{qq}_R(Z^\prime )$ to the quarks. The approximate flavour universality of the latter coupling assures negligible NP contributions to $\mathrm{Re}A_2$. This property, together with the breakdown of the GIM mechanisms at tree level, allows one to enhance significantly the contribution of the leading QCD-penguin operator $Q_6$ to $\mathrm{Re}A_0$. A large fraction of the missing piece in the $\Delta I=1/2$ rule can be explained in this manner for $M_{Z^\prime }$ in the reach of the LHC, while satisfying the constraints from $\varepsilon _K$, $\varepsilon '/\varepsilon$, $\Delta M_K$, LEP-II and the LHC. The presence of a small right-handed flavour-violating coupling $\Delta ^{sd}_R(Z^\prime )\ll \Delta ^{sd}_L(Z^\prime )$ and of enhanced matrix elements of $\Delta S=2$ left–right operators allows one to satisfy simultaneously the constraints from $\mathrm{Re}A_0$ and $\Delta M_K$, although this requires some fine-tuning. We identify the *quartic* correlation between $Z^\prime$ contributions to $\mathrm{Re}A_0$, $\varepsilon '/\varepsilon$, $\varepsilon _K$ and $\Delta M_K$. The tests of this proposal will require much improved evaluations of $\mathrm{Re}A_0$ and $\Delta M_K$ within the SM, of $\langle Q_6 \rangle _0$ as well as precise tree-level determinations of $|V_{ub}|$ and $|V_{cb}|$. We present correlations between $\varepsilon '/\varepsilon$, $K^+\rightarrow \pi ^+\nu \bar{\nu }$ and $K_{L}\rightarrow \pi ^0\nu \bar{\nu }$ with and without the $\Delta I=1/2$ rule constraint and generalise the whole analysis to $Z^\prime$ with colour ($G^\prime$) and $Z$ with FCNC couplings. In the latter case no improvement on $\mathrm{Re}A_0$ can be achieved without destroying the agreement of the SM with the data on $\mathrm{Re}A_2$. Moreover, this scenario is very tightly constrained by $\varepsilon '/\varepsilon$. On the other hand, in the context of the $\Delta I=1/2$ rule $G^\prime$ is even more effective than $Z^\prime$: it provides the missing piece in $\mathrm{Re}A_0$ for $M_{G^\prime }=(3.5$–$4.0)\, \mathrm{TeV}$.

## Introduction

The non-leptonic $K_L\rightarrow \pi \pi$ decays have played already for almost 60 years an important role in particle physics and were instrumental in the construction of the standard model (SM) and in the selection of allowed extensions of this model. The three pillars in these decays are:

- The real parts of the amplitudes $A_I$ for a kaon to decay into two pions with isospin $I$, which are measured to be [1]
  1 $$\begin{aligned} \mathrm{Re}A_0&= 27.04(1)\times 10^{-8}~\, \mathrm{GeV}, \nonumber \\ \mathrm{Re}A_2&= 1.210(2) \times 10^{-8}~\, \mathrm{GeV}, \end{aligned}$$
  and expressing the so-called $\Delta I=1/2$ rule [2, 3],
  2 $$\begin{aligned} R=\frac{\mathrm{Re}A_0}{\mathrm{Re}A_2}=22.35. \end{aligned}$$
- The parameter $\varepsilon _K$, a measure of indirect CP violation in $K_L\rightarrow \pi \pi$ decays, which is found to be
  3 $$\begin{aligned} \varepsilon _K=2.228(11)\times 10^{-3}e^{i\phi _\varepsilon }, \end{aligned}$$
  where $$\phi _\varepsilon =43.51(5)^\circ $$.
- The ratio of the direct CP violation and indirect CP violation in $K_L\rightarrow \pi \pi$ decays measured to be [1, 4–6]
  4 $$\begin{aligned} \mathrm{Re}(\varepsilon '/\varepsilon )=(16.5\pm 2.6)\times 10^{-4}. \end{aligned}$$

Also the strongly suppressed branching ratio for the rare decay $K_L\rightarrow \mu ^+\mu ^-$ and the tiny experimental value for the $K_L-K_S$ mass difference

5 $$\begin{aligned} (\Delta M_{K})_\mathrm{exp} = 3.484(6) 10^{-15} \ \mathrm GeV =5.293(9)\text {ps}^{-1} \end{aligned}$$

were strong motivations for the GIM mechanism [7] and in turn allowed one to predict not only the existence of the charm quark but also approximately its mass [8].

While due to the GIM mechanism $\varepsilon _K$, $\varepsilon '/\varepsilon$ and $\Delta M_K$ receive contributions from the SM dynamics first at one-loop level and as such are sensitive to NP contributions, the $\Delta I=1/2$ rule involving tree-level decays has been expected already for a long time to be governed by SM dynamics. Unfortunately due to non-perturbative nature of non-leptonic decays precise calculation of the amplitudes $\mathrm{Re}A_0$ and $\mathrm{Re}A_2$ do not exist even today. However, a significant progress in reaching this goal over last 40 years has been made.

Indeed, after pioneering calculations of short distance QCD effects in the amplitudes $\mathrm{Re}A_0$ and $\mathrm{Re}A_2$ [9, 10], termed in the past an *octet enhancement*, and the discovery of QCD-penguin operators [11], which in the isospin limit contribute only to $\mathrm{Re}A_0$, the dominant dynamics behind the $\Delta I=1/2$ has been identified in [12]. To this end an *analytic* approximate approach based on the dual representation of QCD as a theory of weakly interacting mesons for large $N$, advocated previously in [15, 16], has been used. In this approach $\Delta I=1/2$ rule for $K\rightarrow \pi \pi$ decays has a simple origin. The octet enhancement through the long but slow quark–gluon renormalisation group evolution down to the scales $\mathcal {O}(1\, \mathrm{GeV})$, analysed first in [9, 10], is continued as a short but fast meson evolution down to zero momentum scales at which the factorisation of hadronic matrix elements is at work. The recent inclusion of lowest-lying vector meson contributions in addition to the pseudoscalar ones and of NLO QCD corrections to Wilson coefficients in a momentum scheme improved significantly the matching between quark–gluon and meson evolutions [17]. In this approach QCD-penguin operators play a subdominant role but one can uniquely predict an enhancement of $\mathrm{Re}A_0$ through QCD-penguin contributions. Working at scales $\mathcal {O}(1\, \mathrm{GeV})$ this enhancement amounts to roughly $15\,\%$ of the experimental value of $\mathrm{Re}A_0$, subject to uncertainties to which we will return below.

In the present era of the dominance of non-perturbative QCD calculations by lattice simulations with dynamical fermions, which have a higher control over uncertainties than the approach in [12, 17], it is very encouraging that the structure of the enhancement of $\mathrm{Re}A_0$ and suppression of $\mathrm{Re}A_2$, identified already in [12], has also been found by RBC-UKQCD collaboration [18–21]. The comparison between the results of both approaches in [17] indicates that the experimental value of the amplitude $\mathrm{Re}A_2$ can be well described within the SM, in particular, as the calculations in these papers have been performed at rather different scales and using a different technology.

On the other hand both approaches cannot presently obtain a sufficiently large value of $\mathrm{Re}A_0$. Within the dual QCD approach one finds then $R=16.0\pm 1.5$, while the first lattice results for $\mathrm{Re}A_0$ imply $R\approx 11$. However, the latter result has been obtained with non-physical kinematics and it is to be expected that larger values of $R$, even as high as its experimental value in (2), could be obtained in lattice QCD in the future.

Presently the theoretical value of $\mathrm{Re}A_0$ within dual QCD approach is by $30\,\%$ below the data and even more in the case of lattice QCD. While this deficit could be the result of theoretical uncertainties in both approaches, it cannot be excluded that the missing piece in $\mathrm{Re}A_0$ comes from NP. In this context we would like to emphasise that, although the explanation of the dynamics behind the $\Delta I=1/2$ rule is not any longer at the frontiers of particle physics, it is important to determine precisely the room for the NP contribution left not only in $\mathrm{Re}A_0$ but also $\mathrm{Re}A_2$. From the present perspective only lattice simulations with dynamical fermions can provide precise values of $\mathrm{Re}A_{0,2}$ one day, but this may still take several years of intensive efforts by the lattice community [22–24]. Having precise SM values for $\mathrm{Re}A_{0,2}$ would give us two observables which could be used to constrain NP. Our paper demonstrates explicitly the impact of such constraints.

In this context we would like to strongly emphasise that, while the dominant part of the $\Delta I=1/2$ rule originates in the SM dynamics, it is legitimate to ask whether some subleading part of it comes from much shorter distance scales and we can either exclude this possibility or demonstrate that this indeed could be the case under certain assumptions.

In what follows our working assumption will be that roughly $30\,\%$ of $\mathrm{Re}A_0$ comes from some kind of NP which does not affect $\mathrm{Re}A_2$ in order not to spoil the agreement of the SM with the data. As the missing piece in $\mathrm{Re}A_0$ is by about 8 times larger than the measured value of $\mathrm{Re}A_2$, the required NP must have a particular structure: tiny or absent contributions to $\mathrm{Re}A_2$ and at the same time large contributions to $\mathrm{Re}A_0$. Moreover, it should satisfy other constraints coming from $\varepsilon _K$, $\Delta M_K$, $\varepsilon '/\varepsilon$ and rare kaon decays.

As $K\rightarrow \pi \pi$ decays originate already at tree level, we expect that NP contributing to these decays at one-loop level will not help us in reaching our goal. Consequently we have to look for NP that contributes to $K\rightarrow \pi \pi$ decays already at tree level as well. Moreover, in order not to spoil the agreement of the SM with the data for $\mathrm{Re}A_2$ only Wilson coefficients of QCD-penguin operators should be modified. In this context we recall that in [25] an additional enhancement (with respect to previous estimates) of the QCD-penguin contributions to $\mathrm{Re}A_0$ has been identified. It comes from an incomplete GIM cancellation above the charm quark mass. But as the analyses in [12, 17] show, this enhancement is insufficient to reproduce fully the experimental value of $\mathrm{Re}A_0$.

However, the observation that the breakdown of GIM mechanism and the enhanced contributions of QCD-penguin operators could in principle provide the missing part of the $\Delta I=1/2$ rule gives us a hint of what kind of NP could do the job here. We have to break the GIM mechanism at a much higher scale than the scales $\mathcal {O}(m_c)$ and allow the QCD renormalisation group evolution to enhance the Wilson coefficient of the leading QCD-penguin operator $Q_6$ by a larger amount than is possible within the SM.

It then turns out that a tree-level exchange of heavy neutral gauge boson, colourless ($Z^\prime$) or carrying colour ($G^\prime$), can provide a significant part of the missing piece of $\mathrm{Re}A_0$ but the couplings of these heavy gauge bosons to SM fermions must have a very special structure in order to satisfy existing constraints from other observables. Let us assume $M_{Z^\prime } (M_{G^\prime })$ to be in the ballpark of a few $\, \mathrm{TeV}$ and let us denote left-handed (LH) and right-handed (RH) couplings of $Z^\prime (G^\prime )$ to two SM fermions with flavours $i$ and $j$, as in [26], by $\Delta _{L,R}^{ij}(Z^\prime )$. Then we find that, in the mass eigenstate basis for all particles involved, a $Z^\prime$ or $G^\prime$ with the following general structure of its couplings is required:

- $\mathrm{Re}\Delta _L^{s d}(Z^\prime )=\mathcal {O}(1)$ and $\mathrm{Re}\Delta _R^{qq}(Z^\prime )=\mathcal {O}(1)$ in order to generate a $Q_6$ penguin operator with sizable Wilson coefficient in the presence of a heavy $Z^\prime$.
- The diagonal couplings $\Delta _R^{qq}(Z^\prime )$ must be flavour universal in order not to affect the amplitude $\mathrm{Re}A_2$. But this universality cannot be exact, as this would not allow one to generate a small $\mathrm{Re}\Delta _R^{s d}(Z^\prime )=\mathcal {O}(10^{-3})$ coupling, which is required in order to satisfy the constraint on $\Delta M_K$ in the presence of $\mathrm{Re}\Delta _L^{s d}(Z^\prime )=\mathcal {O}(1)$.
- $\mathrm{Im}\Delta _L^{s d}(Z^\prime )$ and $\mathrm{Im}\Delta _R^{qq}(Z^\prime )$ must be typically $\mathcal {O}(10^{-3}-10^{-4})$ in order to be consistent with the data on $\varepsilon _K$ and $\varepsilon '/\varepsilon$.
- The couplings to the leptons must be sufficiently small in order not to violate the existing bounds on rare kaon decays. This is automatically satisfied for $G^\prime$.
- Finally, $\Delta _L^{uu}(Z^\prime )$ must be small in order not to generate large contributions to the current–current operators $Q_1$ and $Q_2$ that could affect the amplitude $\mathrm{Re}A_2$.

We observe that indeed the structure of the $Z^\prime$ or $G^\prime$ couplings must be rather special. But in the context of $\varepsilon '/\varepsilon$ it is interesting to note that in this NP scenario, as opposed to many NP scenarios, there is no modification of the Wilson coefficients of electroweak penguin operators up to tiny renormalisation group effects, which can be neglected for all practical purposes. The NP part of $\varepsilon '/\varepsilon$ involves only QCD-penguin operators, in particular $Q_6$, and the size of this effect, as we will demonstrate below, is correlated with the NP contribution to $\mathrm{Re}A_0$, $\varepsilon _K$ and $\Delta M_K$.

Now comes an important point. While the SM contribution to $\mathrm{Re}A_0$ practically does not involve any CKM uncertainties, this is not the case of $\varepsilon _K$, $\varepsilon '/\varepsilon$ and branching ratios on rare kaon decays which all involve potential uncertainties due to present inaccurate knowledge of the elements of the CKM matrix $|V_{ub}|$ and $|V_{cb}|$. Therefore there are uncertainties in the room left for NP in these observables and these uncertainties in turn affect indirectly the allowed size of the NP contribution to $\mathrm{Re}A_0$. Therefore it will be of interest to consider several scenarios for the pair $|V_{ub}|$ and $|V_{cb}|$ and investigate in each case whether $Z^\prime$ couplings required to improve the situation with the $\Delta I=1/2$ rule could also help in explaining the data on $\varepsilon _K$, $\varepsilon '/\varepsilon$, $\Delta M_K$ and rare kaon decays in case the SM would fail to do it one day. Of course presently one cannot reach clear cut conclusions on these matters due to hadronic uncertainties affecting $\varepsilon _K$, $\varepsilon '/\varepsilon$ and $\Delta M_K$ but it is expected that the situation will improve in this decade.

In order to be able to discuss implications for $K^+\rightarrow \pi ^+\nu \bar{\nu }$ and $K_{L}\rightarrow \pi ^0\nu \bar{\nu }$ we will assume in the first part of our paper that $Z^\prime$ is colourless. This is also the case analysed in all our previous $Z^\prime$ papers [26–33]. Subsequently, we will discuss how our analysis changes in the case of $G^\prime$. The fact that in this case $G^\prime$ does not contribute to $K^+\rightarrow \pi ^+\nu \bar{\nu }$ and $K_{L}\rightarrow \pi ^0\nu \bar{\nu }$ allows one already to distinguish this case from the colourless $Z^\prime$ but also the LHC bounds on the couplings of such bosons and the NP contributions to $\mathrm{Re}A_0$, $\varepsilon '/\varepsilon$, $\varepsilon _K$ and $\Delta M_K$ are different in these two cases. In our presentation we will also first assume exact flavour universality for $\Delta _R^{qq}(Z^\prime )$ and $\Delta _R^{qq}(G^\prime )$ couplings in order to demonstrate that in this case the experimental constraints from $\mathrm{Re}A_0$ and $\Delta M_K$ cannot be simultaneously satisfied. Fortunately, already a very small violation of flavour universality in $\Delta _R^{qq}(Z^\prime )$ or $\Delta _R^{qq}(G^\prime )$ allows one to cure this problem because of the enhanced matrix elements of left–right operators contributing in this case to $\Delta M_K$.

Our paper is organised as follows. In Sect. 2 we briefly describe some general aspects of $Z^\prime$ and $G^\prime$ models considered by us. In Sect. 3 we present general formulae for the effective Hamiltonian for $K\rightarrow \pi \pi$ decays including all operators, list the initial conditions for Wilson coefficients at $\mu =M_{Z^\prime }$ for the case of a colourless $Z^\prime$ and find the expressions for $\mathrm{Re}A_0$ and $\varepsilon '/\varepsilon$ that include SM and $Z^\prime$ contributions. In Sect. 4 we discuss briefly $\varepsilon _K$, $\Delta M_K$, $K^+\rightarrow \pi ^+\nu \bar{\nu }$ and $K_{L}\rightarrow \pi ^0\nu \bar{\nu }$, again for a colourless $Z^\prime$, referring for details to our previous papers. In Sect. 5 we present numerical analysis of $\mathrm{Re}A_0$, $\varepsilon '/\varepsilon$ and $K^+\rightarrow \pi ^+\nu \bar{\nu }$ and $K_{L}\rightarrow \pi ^0\nu \bar{\nu }$ taking into account the constraints from $\varepsilon _K$ and $\Delta M_K$. We consider two scenarios. One in which we impose the $\Delta I=1/2$ constraint (scenario A) and one in which we ignore this constraint (scenario B). These two scenarios can be clearly distinguished through the rare decays $K^+\rightarrow \pi ^+\nu \bar{\nu }$ and $K_{L}\rightarrow \pi ^0\nu \bar{\nu }$ and their correlation with $\varepsilon '/\varepsilon$. In Sect. 6 we repeat the full analysis for $G^\prime$ and in Sect. 7 for the $Z$ boson with flavour-violating couplings. We conclude in Sect. 8.

## General aspects of $Z'$ and $G'$ models

The present paper is the continuation of our extensive study of NP represented by a new neutral heavy gauge boson ($Z^\prime$) in the context of a general parametrisation of its couplings to the SM fermions and within specific models like the 331 models [26–33]. The new aspect of the present paper is the generalisation of these studies to $K\rightarrow \pi \pi$ decays with the goal to answer three questions:

- Whether the existence of a $Z^\prime$ or $G^\prime$ with a mass in the reach of the LHC could have an impact on the $\Delta I=1/2$ rule, in particular on the amplitude $\mathrm{Re}A_0$.
- Whether such gauge bosons could have sizable impact on the ratio $\varepsilon '/\varepsilon$.
- What is the impact of $\varepsilon '/\varepsilon$ constraint on FCNC couplings of the SM $Z$ boson.

To our knowledge the first question has not been addressed in the literature, while selected analyses of $\varepsilon '/\varepsilon$ within models with tree-level flavour changing neutral currents can be found in [34, 35]. However, in these papers NP entered $\varepsilon '/\varepsilon$ through electroweak penguin operators while in the case of $Z^\prime$ scenarios considered here only QCD-penguin operators are relevant. Concerning the last point we refer to earlier analyses in [36, 37]. The present paper provides a modern look at this scenario and in particular investigates the sensitivity to the CKM parameters. A review of $Z^\prime$ models can be found in [38] and a collection of papers related mainly to $B_{s,d}$ decays can be found in [26].

Our paper will deal with NP in $K^0$–$\bar{K}^0$ mixing, $K\rightarrow \pi \pi$ and rare $K$ decays dominated either by a heavy $Z^\prime$, heavy $G^\prime$ or FCNC processes mediated by $Z$. We will not provide a complete model in which other fields like heavy vector-like fermions, heavy Higgs scalars and charged gauge bosons are generally present and gauge anomalies are properly cancelled. Examples of such models can be found in [38] and the 331 models analysed by us can be mentioned here [27, 33]. A general discussion can also be found in [39] and among more recent papers we refer to [40, 41]. But none of these papers discusses the hierarchy of the couplings of $Z^\prime$ and $G^\prime$ couplings, which is required to make these gauge bosons to be relevant for the $\Delta I=1/2$ rule. Our goal then is to find this hierarchy first and postpone the construction of a concrete model to a future analysis.

$Z^\prime$ contributions to ${{\mathrm{Re}}A_0}$, ${{\mathrm{Re}}A_2}$ and $\varepsilon '/\varepsilon$ involve generally in addition to $M_{Z^\prime }$ the following couplings:

6 $$\begin{aligned} \Delta _L^{s d}(Z^\prime ),\qquad \Delta _R^{s d}(Z^\prime ), \qquad \Delta _L^{q q}(Z^\prime ),\qquad \Delta _R^{qq}(Z^\prime ), \end{aligned}$$

where $q=u,d,c,s,b,t$. The same applies to $G^\prime$. The diagonal couplings can be generally flavour dependent, but as we already stated above in order to protect the small amplitude ${{\mathrm{Re}}A_2}$ from significant NP contributions in the process of modification of the large amplitude ${{\mathrm{Re}}A_0}$ either the coupling $\Delta _L^{q q}(Z^\prime )$ or the coupling $\Delta _R^{qq}(Z^\prime )$ must be approximately flavour universal. They cannot be both flavour universal as then it would not be possible to generate large flavour-violating couplings in the mass eigenstate basis. In what follows we will assume that $\Delta _R^{qq}(Z^\prime )$ are either exactly flavour universal or flavour universal to a high degree still allowing for a strongly suppressed but non-vanishing coupling $\Delta _R^{s d}(Z^\prime )$.

For the left-handed couplings it will turn out that $\Delta _L^{s d}(Z^\prime )$ $=\mathcal {O}(1)$ in order to reach the first goal on our list. Such a coupling could be in principle generated in the presence of heavy vectorial fermions or other dynamics at scales above $M_{Z^\prime }$. In order to simplify our analysis and reduce the number of free parameters, we will finally assume that $\Delta _L^{q q}(Z^\prime )$ are very small. Thus in summary the hierarchy of couplings in the present paper will be assumed to be as follows:

7 $$\begin{aligned} \Delta _L^{s d}(Z^\prime )&\gg \Delta _L^{qq}(Z^\prime ), \qquad \Delta _R^{sd}(Z^\prime )\ll \Delta _R^{qq}(Z^\prime ),\nonumber \\ \Delta _L^{s d}(Z^\prime )&\gg \Delta _R^{sd}(Z^\prime ) \end{aligned}$$

with the same hierarchy assumed for $G^\prime$.

Only the coupling $\Delta _{L,R}^{s d}(Z^\prime )$ will be assumed to be complex while as we will see in the context of our analysis the remaining two can be assumed to be real without particular loss of generality. We should note that the hierarchy in (7) will suppress in the case of $K\rightarrow \pi \pi$ decays the primed operators that are absent in the SM anyway.

In our previous papers we have considered a number of scenarios for flavour-violating $Z^\prime$ couplings to quarks. These are defined as follows:

1. Left-handed Scenario (LHS) with complex $\Delta _L^{sd}\not =0$ and $\Delta _R^{sd}=0$,
2. Right-handed Scenario (RHS) with complex $\Delta _R^{sd}\not =0$ and $\Delta _L^{sd}=0$,
3. Left–Right symmetric Scenario (LRS) with complex $\Delta _L^{sd}=\Delta _R^{sd}\not =0$,
4. Left–Right asymmetric Scenario (ALRS) with complex $\Delta _L^{sd}=-\Delta _R^{sd}\not =0$.

Among them only the LHS scenario is consistent with (7) if $\Delta _R^{sd}$ is assumed to vanish. But as we will demonstrate in this case it is not possible to satisfy simultaneously the constraints from ${{\mathrm{Re}}A_0}$ and $\Delta M_K$. Consequently $\Delta _R^{sd}$ has to be non-vanishing, although very small, in order to satisfy these two constraints simultaneously. Thus in the scenarios considered in our previous papers the status of the $\Delta I=1/2$ rule cannot be improved with respect to the SM.

## General formulae for $K\rightarrow \pi \pi$ decays

### General structure

Let us begin our presentation with the general formula for the effective Hamiltonian relevant for $K\rightarrow \pi \pi$ decays in the model in question

8 $$\begin{aligned} \mathcal {H}_\mathrm{eff}(K\rightarrow \pi \pi )&= \mathcal {H}_\mathrm{eff}(K\rightarrow \pi \pi )(\mathrm{SM})\nonumber \\&+\, \mathcal {H}_\mathrm{eff}(K\rightarrow \pi \pi )(Z^\prime ) \end{aligned}$$

where the SM part is given by [42]

9 $$\begin{aligned} \begin{aligned}&\mathcal {H}_\mathrm{eff}(K\rightarrow \pi \pi )(\mathrm{SM})=\frac{G_F}{\sqrt{2}}V_{ud}V_{us}^*\sum _{i=1}^{10}(z^\mathrm{SM}_i(\mu )\\&\qquad \qquad \qquad \qquad \qquad \qquad +\tau y^\mathrm{SM}_i(\mu ))Q_i,\\&\quad \tau =-\frac{V_{td}V_{ts}^*}{V_{ud}V_{us}^*}, \end{aligned} \end{aligned}$$

and the operators $Q_i$ as follows:

**Current–Current:**

10 $$\begin{aligned} \begin{aligned} Q_1&= ({\bar{s}}_{\alpha } u_{\beta })_{V-A}\;(\bar{u}_{\beta } d_{\alpha })_{V-A}\\ Q_2&= ({\bar{s}}u)_{V-A}\;(\bar{u}d)_{V-A} \end{aligned} \end{aligned}$$

**QCD-Penguins:**

11 $$\begin{aligned} \begin{aligned} Q_3&= ({\bar{s}} d)_{V-A}\sum _{q=u,d,s,c,b,t}({\bar{q}}q)_{V-A}\\ Q_4&= ({\bar{s}}_{\alpha } d_{\beta })_{V-A}\sum _{q=u,d,s,c,b,t}({\bar{q}}_{\beta } q_{\alpha })_{V-A} \end{aligned} \end{aligned}$$

12 $$\begin{aligned} \begin{aligned} Q_5&= ({\bar{s}} d)_{V-A} \sum _{q=u,d,s,c,b,t}({\bar{q}}q)_{V+A}\\ Q_6&= ({\bar{s}}_{\alpha } d_{\beta })_{V-A}\sum _{q=u,d,s,c,b,t} ({\bar{q}}_{\beta } q_{\alpha })_{V+A} \end{aligned} \end{aligned}$$

**Electroweak Penguins:**

13 $$\begin{aligned} \begin{aligned} Q_7&= \frac{3}{2}\;({\bar{s}} d)_{V-A}\sum _{q=u,d,s,c,b,t}e_q\;({\bar{q}}q)_{V+A}\\ Q_8&= \frac{3}{2}\;({\bar{s}}_{\alpha } d_{\beta })_{V-A}\sum _{q=u,d,s,c,b,t}e_q ({\bar{q}}_{\beta } q_{\alpha })_{V+A} \end{aligned} \end{aligned}$$

14 $$\begin{aligned} \begin{aligned} Q_9&= \frac{3}{2}\;({\bar{s}} d)_{V-A}\sum _{q=u,d,s,c,b,t}e_q({\bar{q}} q)_{V-A}\\ Q_{10}&=\frac{3}{2}\; ({\bar{s}}_{\alpha } d_{\beta })_{V-A}\sum _{q=u,d,s,c,b,t}e_q\; ({\bar{q}}_{\beta }q_{\alpha })_{V-A} \end{aligned} \end{aligned}$$

Here, $\alpha ,\beta$ denote colours and $e_q$ denotes the electric quark charges reflecting the electroweak origin of $Q_7,\ldots ,Q_{10}$. Finally, $({\bar{s}}d)_{V-A}\equiv {\bar{s}}_\alpha \gamma _\mu (1-\gamma _5) d_\alpha$.

The coefficients $z^\mathrm{SM}_i(\mu )$ and $y^\mathrm{SM}_i(\mu )$ are the Wilson coefficients of these operators within the SM. They are known at the NLO level in the renormalisation group improved perturbation theory including both QCD and QED corrections [42, 43]. Also some elements of NNLO corrections can be found in the literature [44, 45].

As discussed in the previous section $Z^\prime$ contributions to $K\rightarrow \pi \pi$ in the class of $Z^\prime$ models discussed by us can be well approximated by the following effective Hamiltonian:

15 $$\begin{aligned} \mathcal {H}_\mathrm{eff}(K\rightarrow \pi \pi )(Z^\prime )=\sum _{i=3}^{6}(C_i(\mu )Q_i+C_i^\prime (\mu ) Q_i^\prime ), \end{aligned}$$

where the primed operators $Q_i^\prime$ are obtained from $Q_i$ by interchanging $V-A$ and $V+A$. For the sake of completeness we keep still $Q_i^\prime$ operators even if at the end due to the hierarchy of couplings in (7), $Z^\prime$ contributions will be well approximated by $Q_i$ and the contributions from the $Q_i^\prime$ operators can be neglected.

Due to the fact that $M_{Z^\prime }\gg m_t$ the summation over flavours in (11)–(14) now includes also the top quark. This structure is valid for both $Z^\prime$ and $G^\prime$. As the hadronic matrix elements of $Q_i$ do not depend on the properties of $Z^\prime$ or $G^\prime$, these two cases can only be distinguished by the values of the coefficients $C_i(\mu )$ and $C_i^\prime (\mu )$. In this and two following sections we analyse the case of $Z^\prime$. But in Sect. 6 we will also discuss $G^\prime$.

The important feature of the effective Hamiltonian in (15) is the absence of $Q_{1,2}$ operators dominating the $A_2$ amplitude and the absence of electroweak penguin operators, which in some of the extensions of the SM are problematic for $\varepsilon '/\varepsilon$. In our model NP effects in $\mathrm{Re}A_0$, relevant for the $\Delta I=1/2$ rule and $\mathrm{Im}A_0$, relevant for $\varepsilon '/\varepsilon$, will enter only through QCD-penguin contributions. This is a novel feature when compared with other scenarios, like the LHT [46] and the Randall–Sundrum scenarios [34, 35], where NP contributions to $\varepsilon '/\varepsilon$ are dominated by electroweak penguin operators. In particular, in the latter case, where FCNCs are mediated by new heavy Kaluza–Klein gauge bosons, the flavour universality of their diagonal couplings to quarks is absent due to different positions of light and heavy quarks in the bulk. Consequently the pattern of NP contributions to $\varepsilon '/\varepsilon$ differs from the one in the models discussed here.

Denoting by $\Delta _{L,R}^{ij}$, as in [26], the couplings of $Z^\prime$ to two quarks with flavours $i$ and $j$, a tree-level $Z^\prime$ exchange generates in our model only the operators $Q_3$, $Q_5$, $Q^\prime _3$ and $Q^\prime _5$ at $\mu =M_{Z^\prime }$. The inclusion of QCD effects, in particular the renormalisation group evolution down to low energy scales, generates the remaining QCD-penguin operators. In principle, using the two-loop anomalous dimensions of [42, 43] and the $\mathcal {O}(\alpha _s)$ corrections to the coefficients $C_i$ and $C_i^\prime$ at $\mu _{Z^\prime }=\mathcal {O}(M_{Z^\prime })$ in the NDR-$\mathrm{\overline{MS}}$ scheme in [47] the full NLO analysis of $Z^\prime$ contributions could be performed. However, due to the fact that the mass of $Z^\prime$ is free and other parametric and hadronic uncertainties, a leading order analysis of NP contributions is sufficient for our purposes. In this manner it will also be possible to see certain properties analytically.

The non-vanishing Wilson coefficients at $\mu =M_{Z^\prime }$ are then given at the LO as follows:

16 $$\begin{aligned} \begin{aligned} C_3(M_{Z^\prime })&= \frac{\Delta _L^{s d}(Z^\prime )\Delta _L^{qq}(Z^\prime )}{4M^2_{Z^\prime }},\\ C_3^\prime (M_{Z^\prime })&= \frac{\Delta _R^{sd}(Z^\prime )\Delta _R^{q q}(Z^\prime )}{4 M^2_{Z^\prime }} , \end{aligned} \end{aligned}$$

17 $$\begin{aligned} \begin{aligned} C_5(M_{Z^\prime })&= \frac{\Delta _L^{sd}(Z^\prime )\Delta _R^{q q}(Z^\prime )}{4M^2_{Z^\prime }},\\ C_5^\prime (M_{Z^\prime })&= \frac{\Delta _R^{sd}(Z^\prime )\Delta _L^{q q}(Z^\prime )}{4 M^2_{Z^\prime }}. \end{aligned} \end{aligned}$$

### Renormalisation group analysis (RG)

With these results at hand we will perform RG analysis of NP contributions at the LO level.^1^ We will then see that the only operator that matters at scales $\mathcal {O}(1 \, \mathrm{GeV})$ in our $Z^\prime$ models is either $Q_6$ or $Q_6^\prime$. This is to be expected if we recall that at $\mu =M_W$ the Wilson coefficient of the electroweak penguin operator $Q_8$, the electroweak analog of $Q_6$, also vanishes. But due to its large anomalous dimension and enhanced hadronic $K\rightarrow \pi \pi$ matrix elements $Q_8$ is by far the dominant electroweak penguin operator in $\varepsilon '/\varepsilon$ within the SM, leaving behind the $Q_7$ operator whose Wilson coefficient does not vanish at $\mu =M_W$. Even if the structure of the present RG analysis differs from the SM one, due to the absence of the remaining operators in the NP part, in particular the absence of $Q_2$, much longer RG evolution from $M_{Z^\prime }$ and not $M_W$ down to low energies makes $Q_6$ or $Q_6^\prime$ the winner at the end. This fact, as we will see, simplifies significantly the phenomenological analysis of the NP contributions to $\mathrm{Re}A_0$ and $\varepsilon '/\varepsilon$.

The relevant $4\times 4$ one-loop anomalous dimension matrix

18 $$\begin{aligned} \hat{\gamma }_s(\alpha _s)=\hat{\gamma }_s^{(0)}\frac{\alpha _s}{4\pi } \end{aligned}$$

can be extracted from the known $6\times 6$ matrix [48]. The evolution of the operators in the NP part is then governed in the $(Q_3,Q_4,Q_5,Q_6)$ basis by

19 $$\begin{aligned} \hat{\gamma }^{(0)}_s = \left( \begin{array}{c@{\quad }c@{\quad }c@{\quad }c} \frac{-22}{9} &{} \frac{22}{3} &{} -\frac{4}{9} &{} \frac{4}{3}\\ 6 - f\frac{2}{9} &{} -2 + f \frac{2}{3} &{} -f\frac{2}{9} &{} f\frac{2}{3} \\ 0 &{} 0 &{} {2} &{} -6 \\ -f \frac{2}{9} &{} f \frac{2}{3} &{} -f\frac{2}{9} &{} -16 + f\frac{2}{3} \end{array}\right) , \end{aligned}$$

where $f$ is the number of effective flavours: $f=6$ for $\mu \ge m_t$ and $f=3$ for $\mu \le m_c$. The same matrix governs the evolution of primed operators.

In order to see what happens analytically we then assume first that in the mass eigenstate basis only the couplings $\Delta _L^{s d}$ and $\Delta _R^{qq}$ are non-vanishing with $\Delta _R^{qq}$ being exactly flavour universal. While the coefficients of the operators $Q_3$ and $Q_4$ can still be generated through RG evolution, these effects are very small and can be neglected. Then to an excellent approximation only the operators $Q_5$ and $Q_6$ matter and the RG evolution is governed by the reduced $2\times 2$ anomalous dimension matrix given in the $(Q_5,Q_6)$ basis as follows:

20 $$\begin{aligned} \hat{\gamma }^{(0)}_s = \left( \begin{array}{c@{\quad }c} 2 &{} -6 \\ -f\frac{2}{9} &{} -16 + f\frac{2}{3} \end{array}\right) . \end{aligned}$$

Denoting then by $\vec {C}(M_{Z^\prime })$ the column vector with components given by the Wilson coefficients $C_5$ and $C_6$ at $\mu =M_{Z^\prime }$ we find their values at $\mu =m_c$ by means of^2^

21 $$\begin{aligned} \vec {C}(m_c)=\hat{U}(m_c,M_{Z^\prime }) \vec {C}(M_{Z^\prime }) \end{aligned}$$

where

22 $$\begin{aligned} \hat{U}(m_c,M_{Z^\prime })&= \hat{U}^{(f=4)}(m_c,m_b)\hat{U}^{(f=5)}(m_b, m_t)\nonumber \\&\times \hat{U}^{(f=6)}(m_t, M_{Z^\prime }) \end{aligned}$$

and [49]

23 $$\begin{aligned} \hat{U}^{(f)}(\mu _1,\mu _2)= \hat{V} \left( {\left[ \frac{\alpha _s(\mu _2)}{\alpha _s(\mu _1)} \right] }^{\frac{\vec \gamma ^{(0)}}{2\beta _0}} \right) _D \hat{V}^{-1}. \end{aligned}$$

Here $$\hat{V}$$ diagonalises $${\hat{\gamma }^{(0)T}}$$,

24 $$\begin{aligned} \hat{\gamma }^{(0)}_D=\hat{V}^{-1} {\hat{\gamma }^{(0)T}} \hat{V} \end{aligned}$$

and $\vec \gamma ^{(0)}$ is the vector containing the diagonal elements of the diagonal matrix:

25 $$\begin{aligned} \hat{\gamma }^{(0)}_D= \left( \begin{array}{cc} \gamma ^{(0)}_+ &{} 0 \\ 0 &{} \gamma ^{(0)}_- \end{array}\right) \end{aligned}$$

with

26 $$\begin{aligned} \beta _0= \frac{33-2f}{3}. \end{aligned}$$

For $\alpha _s(M_Z)=0.1185$, $m_c=1.3\, \mathrm{GeV}$ and $M_{Z^\prime }=3\, \mathrm{TeV}$ we have

27 $$\begin{aligned} \left[ \begin{array}{c} C_5(m_c) \\ C_6(m_c) \end{array}\right]&= \left[ \begin{array}{c@{\quad }c} 0.86 &{} 0.19 \\ 1.13 &{} 3.60 \end{array}\right] \left[ \begin{array}{c} 1 \\ 0 \end{array}\right] \nonumber \\&\times \frac{\Delta _L^{s d}(Z^\prime )\Delta _R^{q q}(Z^\prime )}{4 M^2_{Z^\prime }}. \end{aligned}$$

Consequently

28 $$\begin{aligned} \begin{aligned} C_5(m_c)&= 0.86 \frac{\Delta _L^{s d}(Z^\prime )\Delta _R^{qq}(Z^\prime )}{4 M^2_{Z^\prime }}\\ C_6(m_c)&= 1.13\frac{\Delta _L^{s d}(Z^\prime )\Delta _R^{q q}(Z^\prime )}{4M^2_{Z^\prime }}. \end{aligned} \end{aligned}$$

Due to the large $(1,2)$ element in the matrix (20) and the large anomalous dimension of the $Q_6$ operator represented by the $(2,2)$ element of this matrix, $C_6(m_c)$ is by a factor of $1.3$ larger than $C_5(m_c)$ even if $C_6(M_{Z^\prime })$ vanishes at LO. Moreover, the matrix element $\langle Q_5\rangle _0$ is colour suppressed, which is not the case for $\langle Q_6\rangle _0$, and within a good approximation we can neglect the contribution of $Q_5$. In summary, it is sufficient to keep only $Q_6$ contribution in the decay amplitude in this scenario for $Z^\prime$ couplings.

### The total $A_0$ amplitude

Adding the NP contributions to the SM contribution we find

29 $$\begin{aligned} A_0= A_0^\mathrm{SM}+A_0^\mathrm{NP}, \qquad \end{aligned}$$

with the SM contribution given by

30 $$\begin{aligned} {\mathrm{Re}} A_0^\mathrm{SM}&= \frac{G_F}{\sqrt{2}}\lambda _u\sum _{i=1}^{10} z^\mathrm{SM}_i(\mu )\langle Q_i(\mu )\rangle _0,\end{aligned}$$

31 $$\begin{aligned} {\mathrm{Im}} A_0^\mathrm{SM}&= -\frac{G_F}{\sqrt{2}}{\mathrm{Im}} \lambda _t\sum _{i=3}^{10} y^\mathrm{SM}_i(\mu )\langle Q_i(\mu )\rangle _0. \end{aligned}$$

Here

32 $$\begin{aligned} \lambda _i=V_{id}V_{is}^* \end{aligned}$$

is the usual CKM factor. As NP enters only the Wilson coefficients and

33 $$\begin{aligned} \langle Q^\prime _i(\mu )\rangle _0=-\langle Q_i(\mu )\rangle _0, \end{aligned}$$

the NP contributions can be included by modifying $z_i$ and $y_i$ with $i=3$–$6$ as follows:

34 $$\begin{aligned} \Delta z_i(\mu )=\frac{\sqrt{2}}{\lambda _u G_F}\left( {\mathrm{Re}} C_i(\mu )-{\mathrm{Re}} C^\prime _i(\mu )\right) \end{aligned}$$

and

35 $$\begin{aligned} \Delta y_i(\mu )=-\frac{\sqrt{2}}{{\mathrm{Im}}\lambda _t G_F}\left( {\mathrm{Im}} C_i(\mu )-{\mathrm{Im}} C^\prime _i(\mu )\right) . \end{aligned}$$

In the scenario just discussed only the $Q_6$ operator is relevant and we have

36 $$\begin{aligned} {\mathrm{Re}} A_0^\mathrm{NP}&= \frac{G_F}{\sqrt{2}}\lambda _u \Delta z_6(\mu )\langle Q_6(\mu )\rangle _0\nonumber \\&= {\mathrm{Re}} C_6(\mu )\langle Q_6(\mu )\rangle _0\end{aligned}$$

37 $$\begin{aligned} {\mathrm{Im}} A_0^\mathrm{NP}&= -\frac{G_F}{\sqrt{2}}{\mathrm{Im}}\lambda _t \Delta y_6(\mu )\langle Q_6(\mu )\rangle _0\nonumber \\&= {\mathrm{Im}} C_6(\mu )\langle Q_6(\mu )\rangle _0, \end{aligned}$$

where we have written two equivalent expressions so that one can either work with $z_6$ and $y_6$ as in the SM or directly with the NP coefficient $C_6$. The latter expressions exhibit better the fact that the NP contributions do not depend explicitly on the CKM parameters. For the matrix element $\langle Q_6(\mu )\rangle _0$ we will use the large $N$ result [12, 17]

38 $$\begin{aligned} \langle Q_6(\mu ) \rangle _0=-\,4 \left[ \frac{m_\mathrm{K}^2}{m_s(\mu ) + m_d(\mu )}\right] ^2 (F_K-F_\pi ) \,B_6^{(1/2)},\nonumber \\ \end{aligned}$$

except that we will allow for variation of $B_6^{(1/2)}$ around its strict large $N$ limit $B_6^{(1/2)}=1$. In writing this formula we have removed the factor $\sqrt{2}$ from formula (97) in [17] in order to compensate for the fact that our $F_K$ and $F_\pi$ are larger by this factor relative to their definition in [17]. Their numerical values are given in Table 2.

In our numerical analysis we will use for the quark masses the values from FLAG 2013 [50]

39 $$\begin{aligned} m_s(2\, \mathrm{GeV})&= (93.8\pm 2.4) \, \mathrm{MeV}, \nonumber \\ m_d(2\, \mathrm{GeV})&= (4.68\pm 0.16)\, \mathrm{MeV}. \end{aligned}$$

Then at the nominal value $\mu =m_c=1.3\, \mathrm{GeV}$ we have

40 $$\begin{aligned} m_s(m_c)&= (108.6\pm 2.8) \, \mathrm{MeV}, \nonumber \\ m_d(m_c)&= (5.42\pm 0.18)\, \mathrm{MeV}. \end{aligned}$$

Consequently for $\mu =\mathcal {O}(m_c)$ a useful formula is the following one:

41 $$\begin{aligned} \langle Q_6(\mu ) \rangle _0=-0.50 \, \left[ \frac{114\, \mathrm{MeV}}{m_s(\mu ) + m_d(\mu )}\right] ^2 \,B_6^{(1/2)}\,\, \mathrm{GeV}^3.\nonumber \\ \end{aligned}$$

The final expressions for $Z^\prime$ contributions to $A_0$ are

42 $$\begin{aligned} {\mathrm{Re}} A_0^\mathrm{NP}={{\mathrm{Re}} \Delta _L^{sd}}(Z^\prime ) K_6(M_{Z^\prime }) \left[ 1.4\times 10^{-8}\, \mathrm{GeV}\right] ,\end{aligned}$$

43 $$\begin{aligned} {\mathrm{Im}} A_0^\mathrm{NP}={{\mathrm{Im}}\Delta _L^{sd}}(Z^\prime ) K_6(M_{Z^\prime }) \left[ 1.4\times 10^{-8}\, \mathrm{GeV}\right] , \end{aligned}$$

where we have defined the $\mu$-independent factor

44 $$\begin{aligned} K_6(M_{Z^\prime })&= -r_6(\mu ) \Delta _R^{qq}(Z^\prime )\, \left[ \frac{3\, \mathrm{TeV}}{M_{Z^\prime }}\right] ^2 \nonumber \\&\times \left[ \frac{114\, \mathrm{MeV}}{m_s(\mu ) + m_d(\mu )}\right] ^2 \,B_6^{(1/2)}\, \end{aligned}$$

with the renormalisation group factor $r_6(\mu )$ defined by

45 $$\begin{aligned} C_6(\mu ) = \frac{\Delta _L^{s d}(Z^\prime )\Delta _R^{q q}(Z^\prime )}{4 M^2_{Z^\prime }} r_6(\mu ). \end{aligned}$$

For $\mu =1.3\, \mathrm{GeV}$, as seen in (28), we find $r_6=1.13$.

Demanding now that $P\%$ of the experimental value of $\mathrm{Re}A_0$ in (1) comes from the $Z^\prime$ contribution, we arrive at the condition:

46 $$\begin{aligned} \mathrm{Re} \Delta _L^{sd}(Z^\prime ) K_6(Z^\prime ) = 3.9\, \left[ \frac{P\%}{20\%}\right] . \end{aligned}$$

Evidently the couplings $\mathrm{Re} \Delta _L^{sd}$ and $\Delta _R^{q q}(Z^\prime )$ must have opposite signs and must satisfy

47 $$\begin{aligned} \mathrm{Re} \Delta _L^{sd}(Z^\prime )\Delta _R^{q q}(Z^\prime )\left[ \frac{3\, \mathrm{TeV}}{M_{Z^\prime }}\right] ^2B_6^{(1/2)}= -3.4\, \left[ \frac{P\%}{20\%}\right] .\nonumber \\ \end{aligned}$$

We also find

48 $$\begin{aligned} {\mathrm{Im}} A_0^\mathrm{NP}=\frac{\mathrm{Im}\Delta _L^{sd}}{{\mathrm{Re}} \Delta _L^{sd}} \left[ \frac{P\%}{20\,\%}\right] \left[ 5.4\times 10^{-8}\, \mathrm{GeV}\right] , \end{aligned}$$

with implications for $\varepsilon '/\varepsilon$ which we will discuss below.

From (47) we observe that for $M_{Z^\prime }\approx 3\, \mathrm{TeV}$ and $B_6^{(1/2)}=1.0\,\pm \,0.25$ as expected from the large-$N$ approach, the product $|\mathrm{Re} \Delta _L^{sd}(Z^\prime )\mathrm{Re} \Delta _R^{qq}(Z^\prime )|$ must be larger than unity unless $P$ is smaller than $7$. The strongest bounds on $\mathrm{Re} \Delta _L^{sd}(Z^\prime )$ come from $\Delta M_K$ while the ones on $\mathrm{Re} \Delta _R^{qq}(Z^\prime )$ from the LHC.

In what follows we will discuss first $\varepsilon '/\varepsilon$, subsequently $\varepsilon _K$ and $\Delta M_K$ and finally in Sect. 5 the constraints from the LHC.

### The ratio $\varepsilon '/\varepsilon$

#### Preliminaries

The ratio $\varepsilon '/\varepsilon$ measures the size of the direct CP violation in $K_L\rightarrow \pi \pi$ relative to the indirect CP violation described by $\varepsilon _K$. In the SM $\varepsilon ^\prime$ is governed by QCD penguins but receives also an important destructively interfering contribution from electroweak penguins that is generally much more sensitive to NP than the QCD-penguin contribution. The interesting feature of NP presented here is that the electroweak penguin part of $\varepsilon '/\varepsilon$ remains as in the SM and only the QCD-penguin part gets modified.

The big challenge in making predictions for $\varepsilon '/\varepsilon$ within the SM and its extensions is the strong cancellation of QCD-penguin contributions and electroweak penguin contributions to this ratio. In the SM QCD-penguins give positive contribution, while the electroweak penguins negative one. In order to obtain useful prediction for $\varepsilon '/\varepsilon$ in the SM the corresponding hadronic parameters $B_6^{(1/2)}$ and $B_8^{(3/2)}$ have to be known with the accuracy of at least $10\,\%$. Recently significant progress has been made by RBC-UKQCD collaboration in the case of $B_8^{(3/2)}$ that is relevant for electroweak penguin contribution [20] but the calculation of $B_6^{(1/2)}$, which will enter our analysis is even more important. There are some hopes that also this parameter could be known from lattice QCD with satisfactory precision in this decade [24, 51].

On the other hand the calculations of short distance contributions to this ratio (Wilson coefficients of QCD and electroweak penguin operators) within the SM have been known already for 20 years at the NLO level [42, 43] and present technology could extend them to the NNLO level if necessary. First steps in this direction have been done in [44, 45]. As we have seen above due to the NLO calculations in [47] a complete NLO analysis of $\varepsilon '/\varepsilon$ can also be performed in the NP models considered here.

Selected analyses of $\varepsilon '/\varepsilon$ in various extensions of the SM and its correlation with $\varepsilon _K$, $K^+\rightarrow \pi ^+\nu \bar{\nu }$ and $K_{L}\rightarrow \pi ^0\nu \bar{\nu }$ can be found in [35–37, 46]. Useful information can also be found in [52–56].

#### $\varepsilon '/\varepsilon$ in the standard model

In the SM all QCD-penguin and electroweak penguin operators in (11)–(14) contribute to $\varepsilon '/\varepsilon$. The NLO renormalisation group analysis of these operators is rather involved [42, 43] but eventually one can derive an analytic formula for $\varepsilon '/\varepsilon$ [53] in terms of the basic one-loop functions

49 $$\begin{aligned} X_0(x_t)&= {\frac{x_t}{8}}\left[ {\frac{x_t+2}{x_t-1}} + {\frac{3 x_t-6}{(x_t -1)^2}}\; \ln x_t\right] ,\end{aligned}$$

50 $$\begin{aligned} Y_0(x_t)&= {\frac{x_t}{8}}\left[ {\frac{x_t -4}{x_t-1}} + {\frac{3 x_t}{(x_t -1)^2}} \ln x_t\right] ,\end{aligned}$$

51 $$\begin{aligned} Z_0(x_t)&= -{\frac{1}{9}}\ln x_t + {\frac{18x_t^4-163x_t^3 + 259x_t^2-108x_t}{144 (x_t-1)^3}}\nonumber \\&+\,{\frac{32x_t^4-38x_t^3-15x_t^2+18x_t}{72(x_t-1)^4}}\ln x_t\end{aligned}$$

52 $$\begin{aligned} E_0(x_t)&= -{\frac{2}{3}}\ln x_t+{\frac{x_t^2(15-16x_t+4x_t^2)}{6(1-x_t)^4}} \ln x_t\nonumber \\&+{\frac{x_t(18-11x_t-x_t^2)}{12(1-x_t)^3}}, \end{aligned}$$

where $$x_t=m^2_t/M_W^2$$.

The updated version of this formula used in the present paper is given as follows:

53 $$\begin{aligned} \left( \frac{\varepsilon '}{\varepsilon }\right) _\mathrm{SM}= a\,\mathrm{Im}\lambda _t \cdot F_{\varepsilon '}(x_t) \end{aligned}$$

where $a=0.92\,\pm \,0.03$ represents the correction coming from the $\Delta I=5/2$ transitions [57], which has not been included in [53]. Next

54 $$\begin{aligned} F_{\varepsilon '}(x_t)&= P_0 + P_X \, X_0(x_t) + P_Y \, Y_0(x_t)\nonumber \\&+ P_Z \, Z_0(x_t)+ P_E \, E_0(x_t), \end{aligned}$$

with the first term dominated by QCD-penguin contributions, the next three terms by electroweak penguin contributions and the last term being totally negligible. The coefficients $P_i$ are given in terms of the non-perturbative parameters $R_6$ and $R_8$ defined in (56) as follows:

55 $$\begin{aligned} P_i = r_i^{(0)} + r_i^{(6)} R_6 + r_i^{(8)} R_8. \end{aligned}$$

The coefficients $r_i^{(0)}$, $r_i^{(6)}$ and $r_i^{(8)}$ comprise information on the Wilson-coefficient functions of the $\Delta S=1$ weak effective Hamiltonian at the NLO. Their numerical values extracted from [53] are given in the NDR renormalisation scheme for $\mu =m_c$ and three values of $\alpha _s(M_Z)$ in Table 1.^3^ While other values of $\mu$ could be considered, the procedure for finding the coefficients $r_i^{(0)}$, $r_i^{(6)}$ and $r_i^{(8)}$ is most straightforward at $\mu =m_c$.

The details on the procedure in question can be found in [42, 53]. In particular in obtaining the numerical values in Table 1 the experimental value for ${\mathrm{Re}} A_2$ has been imposed to determine hadronic matrix elements of subleading electroweak penguin operators ($Q_9$ and $Q_{10}$). The matrix elements of $(V-A)\otimes (V-A)$ penguin operators have been bounded by relating them to the matrix elements $\langle Q_{1,2}\rangle _0$ that govern the octet enhancement of ${\mathrm{Re}} A_0$. Moreover, as $\varepsilon '/\varepsilon$ involves ${\mathrm{Re}} A_0$ also this amplitude has been taken from experiment. This procedure can also be used in $Z^\prime$ models as here experimental value of ${\mathrm{Re}} A_0$ will constitute an important constraint and the contributions of operators $Q_9$ and $Q_{10}$ are unaffected by new $Z^\prime$ contributions up to tiny $\mathcal {O}(\alpha )$ effects from mixing with the operator $Q_6$.

The dominant dependence on the hadronic matrix elements in $\varepsilon '/\varepsilon$ resides in the QCD-penguin operator $Q_6$ and the electroweak penguin operator $Q_8$. Indeed from Table 1 we find that the largest are the coefficients $r_0^{(6)}$ and $r_Z^{(8)}$ representing QCD-penguin and electroweak penguin contributions, respectively. The fact that these coefficients are of similar size but having opposite signs has been a problem since the end of 1980s when the electroweak penguin contribution increased in importance due to the large top-quark mass [58, 59].

The parameters $R_6$ and $R_8$ are directly related to the parameters $B_6^{(1/2)}$ and $B_8^{(3/2)}$ representing the hadronic matrix elements of $Q_6$ and $Q_8$, respectively. They are defined as

56 $$\begin{aligned} \begin{aligned} R_6&\equiv 1.13\,B_6^{(1/2)}\left[ \frac{114\, \mathrm{MeV}}{m_s(m_c)+m_d(m_c)}\right] ^2,\\ R_8&\equiv 1.13\,B_8^{(3/2)}\left[ \frac{114\, \mathrm{MeV}}{m_s(m_c)+m_d(m_c)} \right] ^2, \end{aligned} \end{aligned}$$

where the factor $1.13$ signals the decrease of the value of $m_s$ since the analysis in [53] has been done.

There is no reliable result on $B_6^{(1/2)}$ from lattice QCD. On the other hand one can extract the lattice value for $B_8^{(3/2)}$ from [21]. We find

57 $$\begin{aligned} B_8^{(3/2)}(3\, \mathrm{GeV})= 0.65\pm 0.05 \qquad \mathrm{(lattice)}. \end{aligned}$$

As $B_8^{(3/2)}$ depends very weakly on the renormalisation scale [42], the same value can be used at $\mu =m_c$. In the absence of the value for $B_6^{(1/2)}$ from lattice results, we will investigate how the result on $\varepsilon '/\varepsilon$ changes when $B_6^{(1/2)}$ is varied within $25\,\%$ from its large $N$ value $B_6^{(1/2)}=1$ [25]. Similar to $B_8^{(3/2)}$, the parameter $B_6^{(1/2)}$ exhibits a very weak $\mu$ dependence [42].

#### $Z^\prime$ contribution to $\varepsilon '/\varepsilon$

We will next present $Z^\prime$ contributions to $\varepsilon '/\varepsilon$. A straight forward calculation gives

58 $$\begin{aligned} \left( \frac{\varepsilon '}{\varepsilon }\right) _{Z^\prime }= -\frac{{\mathrm{Im}} A_0^\mathrm{NP}}{{\mathrm{Re}}A_0}\left[ \frac{\omega _+}{|\varepsilon _K|\sqrt{2}}\right] (1-\Omega _\mathrm{eff}), \end{aligned}$$

where [57]

59 $$\begin{aligned} \begin{aligned} \omega _+&=a\frac{{\mathrm{Re}} A_2}{{\mathrm{Re}}A_0}=(4.1\pm 0.1)\times 10^{-2},\\ \Omega _\mathrm{eff}&=(6.0\pm 7.7)\times 10^{-2}. \end{aligned} \end{aligned}$$

In order to obtain the first number we set $a=0.92\pm 0.02$ and as in the case of the SM we use the experimental values for $\mathrm{Re} A_0$ and $\mathrm{Re} A_2$ in (1). Also the experimental values for $|\varepsilon _K|$ and $\mathrm{Re}A_0$ should be used in (58).

The final expression for $\varepsilon '/\varepsilon$ is given by

60 $$\begin{aligned} \left( \frac{\varepsilon '}{\varepsilon }\right) _\mathrm{tot}=\left( \frac{\varepsilon '}{\varepsilon }\right) _\mathrm{SM}+\left( \frac{\varepsilon '}{\varepsilon }\right) _{Z^\prime } \end{aligned}$$

#### Correlation between $Z^\prime$ contributions to $\varepsilon '/\varepsilon$ and $\mathrm{Re}A_0$

In our favourite scenarios only the couplings $\Delta _L^{sd}(Z^\prime )$, $\Delta _R^{qq}(Z^\prime )$ and the operator $Q_6$ will be relevant in $K\rightarrow \pi \pi$ decays. In this case the expressions presented above allow one to derive the relation

61 $$\begin{aligned} \begin{aligned} \left( \frac{\varepsilon '}{\varepsilon }\right) _{Z^\prime }&= -12.3 \left[ \frac{\mathrm{Re}A^\mathrm{NP}_0}{\mathrm{Re}A_0}\right] \left[ \frac{{\mathrm{Im}\Delta _L^{sd}(Z^\prime )}}{{\mathrm{Re}\Delta _L^{sd}(Z^\prime )}}\right] \\&= -2.5\, \left[ \frac{P\%}{20\,\%}\right] \left[ \frac{{\mathrm{Im}\Delta _L^{sd}(Z^\prime )}}{{\mathrm{Re}\Delta _L^{sd}(Z^\prime )}}\right] , \end{aligned} \end{aligned}$$

which is free from the uncertainties in the CKM matrix and $\langle Q_6\rangle _0$. But the most important message that follows from this relation is that

62 $$\begin{aligned} \left[ \frac{{\mathrm{Im}\Delta _L^{sd}(Z^\prime )}}{{\mathrm{Re}\Delta _L^{sd}(Z^\prime )}}\right] =\mathcal {O}(10^{-4}) \end{aligned}$$

if we want to obtain $20\,\%$ shift in $\mathrm{Re}A_0$ and simultaneously be consistent with the data on $\varepsilon '/\varepsilon$. This also implies that $Z^\prime$ contributions to $\varepsilon _K$ and $K_{L}\rightarrow \pi ^0\nu \bar{\nu }$ which require complex CP-violating phases will be easier to keep under control than it is the case of $\Delta M_K$ and $K^+\rightarrow \pi ^+\nu \bar{\nu }$, which are CP conserving. In order to put these expectations on a firm footing we now have to discuss $\varepsilon _K$, $\Delta M_K$ and $K\rightarrow \pi \nu \bar{\nu }$.

## Constraints from $\varepsilon _K$, $\Delta M_K$ and $K\rightarrow \pi \nu \bar{\nu }$

### $\varepsilon _K$ and $\Delta M_K$

In the models in question we have

63 $$\begin{aligned} \begin{aligned}&\Delta M_K=(\Delta M_K)_\text {SM}+\Delta M_K(Z^\prime ),\\&\varepsilon _K =(\varepsilon _K)_\text {SM}+\varepsilon _K(Z^\prime ) \end{aligned} \end{aligned}$$

and similar for $G^\prime$. A very detailed analysis of these observables in a general $Z^\prime$ model with $\Delta _L^{s d}(Z^\prime )$ and $\Delta _R^{s d}(Z^\prime )$ couplings in LHS, RHS, LRS and ALRS scenarios has been presented in [26]. We will not repeat the relevant formulae for $\varepsilon _K$ and $\Delta M_K$, which can be found there. Still it is useful to recall the operators contributing in the general case. These are

64 $$\begin{aligned}&{Q}_1^\text {VLL}=\left( {\bar{s}}\gamma _\mu P_L d\right) \left( {\bar{s}}\gamma ^\mu P_Ld\right) ,\nonumber \\&{Q}_1^\text {VRR}=\left( {\bar{s}}\gamma _\mu P_R d\right) \left( {\bar{s}}\gamma ^\mu P_R d\right) ,\end{aligned}$$

65 $$\begin{aligned}&{Q}_1^\text {LR}=\left( {\bar{s}}\gamma _\mu P_L d\right) \left( {\bar{s}}\gamma ^\mu P_R d\right) ,\nonumber \\&{Q}_2^\text {LR}=\left( {\bar{s}} P_L d\right) \left( {\bar{s}} P_R d\right) , \end{aligned}$$

where $P_{R,L}=(1\pm \gamma _5)/2$ and we suppressed the colour indices as they are summed up in each factor. For instance ${\bar{s}}\gamma _\mu P_L d$ stands for ${\bar{s}}_\alpha \gamma _\mu P_L d_\alpha$ and similarly for other factors. In the SM only ${Q}_1^\text {VLL}$ is present. This operator basis applies also to $G^\prime$ but the Wilson coefficients of these operators at $\mu =M_{G^\prime }$ will be different as we will see in Sect. 6.

If only the Wilson coefficient of the operator ${Q}_1^\text {VLL}$ is affected by $Z^\prime$ contributions, as is the case of the LHS scenario, then the NP effects in $\varepsilon _K$ and $\Delta M_K$ can be summarised by the modification of the one-loop function $S$:

66 $$\begin{aligned} S(K)=S_0(x_t)+\Delta S(K) \end{aligned}$$

with the SM contribution represented by

67 $$\begin{aligned} S_0(x_t)&= \frac{4x_t - 11 x_t^2 + x_t^3}{4(1-x_t)^2}-\frac{3 x_t^2\log x_t}{2 (1-x_t)^3}\nonumber \\&= 2.31 \left[ \frac{m_t(m_t)}{163\, \mathrm{GeV}}\right] ^{1.52} \end{aligned}$$

and the one from $Z^\prime$ by

68 $$\begin{aligned} \Delta S(K)&= \left[ \frac{\Delta _L^{sd}(Z^\prime )}{\lambda _t}\right] ^2 \frac{4\tilde{r}}{M^2_{Z^\prime }g_{\text {SM}}^2},\nonumber \\ g_{\text {SM}}^2&= 4\frac{G_F}{\sqrt{2}}\frac{\alpha }{2\pi \sin ^2\theta _W}=1.781\times 10^{-7}\,\, \mathrm{GeV}^{-2}. \end{aligned}$$

Here $\tilde{r}$ is a QCD factor calculated in [28] at the NLO level. One finds $\tilde{r}=0.965$, $\tilde{r}=0.953$ and $\tilde{r} = 0.925$ for $M_{Z^\prime } =2,~3, ~10\, \mathrm{TeV}$, respectively. Neglecting logarithmic scale dependence of $\tilde{r}$ we find then

69 $$\begin{aligned} \Delta S(K)=2.4 \left[ \frac{\Delta _L^{sd}(Z^\prime )}{\lambda _t}\right] ^2 \left[ \frac{3\, \mathrm{TeV}}{M_{Z^\prime }}\right] ^2. \end{aligned}$$

For $\Delta ^{sd}_L(Z^\prime )$ with a small phase, as in (62), one can still satisfy the $\varepsilon _K$ constraint, but if we want to explain $30\,\%$ of $\mathrm{Re} A_0$ the bound from $\Delta M_K$ is violated by several orders of magnitude. Indeed allowing conservatively that the NP contribution is at most as large as the short distance SM contribution to $\Delta M_K$ we find the bound on a real $\Delta ^{sd}_L(Z^\prime )$

70 $$\begin{aligned} |\Delta ^{sd}_L(Z^\prime )|&\le 0.65|V_{us}|\sqrt{\frac{\eta _{cc}}{\eta _{tt}}}\frac{m_c}{M_W} \left[ \frac{M_{Z^\prime }}{3\, \mathrm{TeV}}\right] \nonumber \\&= 0.004\, \left[ \frac{M_{Z^\prime }}{3\, \mathrm{TeV}}\right] . \end{aligned}$$

This bound, as seen in (46), does not allow any significant contribution to occur to $\mathrm{Re} A_0$ unless the coupling $\Delta _R^{qq}$ and or $B_6^{(1/2)}$ are very large. We also note that the increase of $M_{Z^\prime }$ makes the situation even worse because the required value of $\mathrm{Re} \Delta ^{sd}_L(Z^\prime )$ by the condition (46) grows quadratically with $M_{Z^\prime }$, whereas this mass enters only linearly in (70). Evidently the LHS scenario does not provide any relevant NP contribution to $\mathrm{Re} A_0$ when the constraint from $\Delta M_K$ is imposed. On the other hand in this scenario still interesting results for $\varepsilon '/\varepsilon$, $K^+\rightarrow \pi ^+\nu \bar{\nu }$ and $K_{L}\rightarrow \pi ^0\nu \bar{\nu }$ can be obtained.

In order to remove the incompatibility of $\mathrm{Re} A_0$ and $\Delta M_K$ constraints we have to suppress somehow $Z^\prime$ contribution to $\Delta M_K$ in the presence of a coupling $\Delta ^{sd}_L(Z^\prime )$ that is sufficiently large so that the contribution of $Z^\prime$ to $\mathrm{Re} A_0$ is relevant. To this end we introduce an effective $[\Delta ^{sd}_L(Z^\prime )]_\mathrm{eff}$ to be used only in $\Delta S=2$ transitions and given by

71 $$\begin{aligned}{}[\Delta ^{sd}_L(Z^\prime )]_\mathrm{eff}=\Delta ^{sd}_L(Z^\prime )\delta \end{aligned}$$

with $\Delta ^{sd}_L(Z^\prime )$ still denoting the coupling used for the evaluation of $\mathrm{Re} A_0$ and $\delta$ a suppression factor. We do not care about the sign of $\Delta ^{sd}_L(Z^\prime )$, which can be adjusted by the sign of $\Delta _R^{qq}(Z^\prime )$. Imposing then the constraint (46) but demanding that simultaneously (70) is satisfied with $\Delta ^{sd}_L(Z^\prime )$ replaced by $[\Delta ^{sd}_L(Z^\prime )]_\mathrm{eff}$ we find that the required $\delta$ is given as follows:

72 $$\begin{aligned} \delta =\left[ \frac{r_6(m_c)}{1.13}\right] \Delta _R^{qq}(Z^\prime ), \left[ \frac{3\, \mathrm{TeV}}{M_{Z^\prime }}\right] B_6^{(1/2)}\left[ \frac{20\,\%}{P\%}\right] 10^{-3}.\nonumber \\ \end{aligned}$$

Here we neglected the small uncertainty in the quark masses. Evidently, increasing simultaneously $\Delta _R^{qq}(Z^\prime )$ and $B_6^{(1/2)}$ to above unity, decreasing $M_{Z^\prime }$ to below $3\, \mathrm{TeV}$ and $P$ to below $20\,\%$ can increase $\delta$ but then one has to check the other constraints, in particular from the LHC. We will study this issue below.

Such a small $\delta$ can be generated in the presence of flavour-violating right-handed couplings in addition to the left-handed ones. In this case at NLO the values of the Wilson coefficients of $\Delta S=2$ operators at $\mu =M_{Z^\prime }$ generated through $Z^\prime$ tree-level exchange are given in the NDR scheme as follows [60]:

73 $$\begin{aligned}&C_1^\text {VLL}(M_{Z^\prime })=\frac{(\Delta _L^{sd}(Z^\prime ))^2}{2M_{Z^\prime }^2}\left( 1+\frac{11}{3}\frac{\alpha _s(M_{Z^\prime })}{4\pi }\right) ,\end{aligned}$$

74 $$\begin{aligned}&C_1^\text {VRR}(M_{Z^\prime }) =\frac{(\Delta _R^{sd}(Z^\prime ))^2}{2M_{Z^\prime }^2}\left( 1+\frac{11}{3}\frac{\alpha _s(M_{Z^\prime })}{4\pi }\right) ,\end{aligned}$$

75 $$\begin{aligned}&C_1^\text {LR}(M_{Z^\prime }) =\frac{\Delta _L^{sd}(Z^\prime )\Delta _R^{sd}(Z^\prime )}{M_{Z^\prime }^2}\left( 1-\frac{1}{6}\frac{\alpha _s(M_{Z^\prime })}{4\pi }\right) ,\end{aligned}$$

76 $$\begin{aligned}&C_2^\text {LR}(M_{Z^\prime })= -\frac{\Delta _L^{sd}(Z^\prime )\Delta _R^{sd}(Z^\prime )}{M_{Z^\prime }^2}\frac{\alpha _s(M_{Z^\prime })}{4\pi }. \end{aligned}$$

The information about hadronic matrix elements of these operators calculated by various lattice QCD collaborations is given in the review [61].

Now, it is well known that similar to $Q_6$ and $Q_6^\prime$, the LR operators have in the case of the $K$ meson system chirally enhanced matrix elements over those of VLL and VRR operators; and as the LR operators have also large anomalous dimensions, their contributions to $\varepsilon _K$ and $\Delta M_K$ dominate the NP contributions in LRS and ALRS scenarios, while they are absent in the LHS and RHS scenarios.

In order to see how the problem with $\Delta M_K$ is solved in this case we calculate $\Delta M_K$ in a general case assuming for simplicity that the couplings $\Delta _{L,R}(Z^\prime )$ are real. We find

77 $$\begin{aligned}&\Delta M_K(Z^\prime ) = \frac{(\Delta _L^{sd}(Z^\prime ))^2}{M_{Z^\prime }^2} \langle \hat{Q}_1^\text {VLL}(M_{Z^\prime })\rangle \nonumber \\&\quad \times \left[ 1+\left( \frac{\Delta _R^{sd}(Z^\prime )}{\Delta _L^{sd}(Z^\prime )}\right) ^2 + 2\left( \frac{\Delta _R^{sd}(Z^\prime )}{\Delta _L^{sd}(Z^\prime )}\right) \frac{\langle \hat{Q}_1^\text {LR}(M_{Z^\prime })\rangle }{\langle \hat{Q}_1^\text {VLL}(M_{Z^\prime })\rangle }\right] ,\nonumber \\ \end{aligned}$$

where using the technology in [60, 62] we have expressed the final result in terms of the renormalisation scheme independent matrix elements,

78 $$\begin{aligned} \langle \hat{Q}_1^\text {VLL}(M_{Z^\prime })\rangle&= \langle Q_1^\text {VLL}(M_{Z^\prime })\rangle \left( 1+\frac{11}{3}\frac{\alpha _s(M_{Z^\prime })}{4\pi }\right) \end{aligned}$$

79 $$\begin{aligned} \langle \hat{Q}_1^\text {LR}(M_{Z^\prime })\rangle&= \langle Q_1^\text {LR}(M_{Z^\prime })\rangle \left( 1-\frac{1}{6}\frac{\alpha _s(M_{Z^\prime })}{4\pi }\right) \nonumber \\&-\frac{\alpha _s(M_{Z^\prime })}{4\pi }\langle Q_2^\text {LR}(M_{Z^\prime })\rangle . \end{aligned}$$

Here $\langle Q_1^\text {VLL}(M_{Z^\prime })\rangle$ and $\langle Q_{1,2}^\text {LR}(M_{Z^\prime })\rangle$ are the matrix elements evaluated at $\mu =M_{Z^\prime }$ in the NDR scheme and the presence of $\mathcal {O}(\alpha _s)$ corrections removes the scheme dependence.

But in the case of $K^0-\bar{K}^0$ matrix elements for $\mu =M_{Z^\prime }=3\, \mathrm{TeV}$

80 $$\begin{aligned} \begin{aligned}&\langle \hat{Q}^\text {VLL}(M_{Z^\prime })\rangle >0, \quad \langle \hat{Q}_1^\text {LR}(M_{Z^\prime })\rangle <0, \\&|\langle \hat{Q}_1^\text {LR}(M_{Z^\prime })\rangle |\approx 97 \, |\langle \hat{Q}^\text {VLL}(M_{Z^\prime })\rangle |. \end{aligned} \end{aligned}$$

The signs are independent of the scale $\mu =M_{Z^\prime }$ but the numerical factor in the last relation increases logarithmically with this scale. Consequently in LR and ALR scenarios the last term in (77) dominates so that the problem with $\Delta M_K$ is even worse. We conclude therefore that in LHS, RHS, LRS and ALRS scenarios analysed in our previous papers [26–33], the problem in question remains.

On the other hand we note that for a non-vanishing but small $\Delta _R^{sd}(Z^\prime )$ coupling

81 $$\begin{aligned} \delta \!=\!\left[ 1\!+\!\left( \frac{\Delta _R^{sd}(Z^\prime )}{\Delta _L^{sd}(Z^\prime )}\right) ^2\!+\!2\left( \frac{\Delta _R^{sd}(Z^\prime )}{\Delta _L^{sd}(Z^\prime )}\right) \frac{\langle \hat{Q}_1^\text {LR}(M_{Z^\prime })\rangle }{\langle \hat{Q}_1^\text {VLL}(M_{Z^\prime })\rangle }\right] ^{\!1/2},\!\!\nonumber \\ \end{aligned}$$

can be made very small and $Z^\prime$ contribution to $\Delta M_K$ and also $\varepsilon _K$ can be suppressed sufficiently and even totally eliminated.

In order to generate a non-vanishing $\Delta _R^{sd}(Z^\prime )$ in the mass eigenstate basis the exact flavour universality has to be violated generating a small contribution to $\mathrm{Re} A_2$ but in view of the required size of $\Delta _R^{sd}(Z^\prime )=\mathcal {O}(10^{-3})$ this effect can be neglected. Thus the presence of a small $\Delta _R^{sd}(Z^\prime )$ coupling has basically no impact on $K\rightarrow \pi \pi$ decays and serves only to avoid the problem with $\Delta M_K$ which we found in the LHS scenario. Even if this solution appears at first sight to be fine-tuned, its existence is interesting. Therefore we will analyse it numerically below for a $Z^\prime$ in a toy model for the coupling $\Delta _R^{sd}(Z^\prime )$ which satisfies (81) but allows for a non-vanishing $\delta$. The case of $G^\prime$ will be analysed in Sect. 6.

### $K^+\rightarrow \pi ^+\nu \bar{\nu }$ and $K_{L}\rightarrow \pi ^0\nu \bar{\nu }$

A very detailed analysis of these decays in a general $Z^\prime$ model with $\Delta _L^{s d}(Z^\prime )$ and $\Delta _R^{s d}(Z^\prime )$ couplings in various combinations has been presented in [26] and we will use the formulae of that paper. Still it is useful to recall the expression for the shift caused by $Z^\prime$ tree-level exchanges in the relevant function $X(K)$. One has now

82 $$\begin{aligned} X(K)=X_0(x_t)+ \Delta X(K) \end{aligned}$$

with $X_0(x_t)$ given in (49) and $Z^\prime$ contribution by

83 $$\begin{aligned} \Delta X(K)=\left[ \frac{\Delta _L^{\nu \nu }(Z')}{g^2_\mathrm{SM}M_{Z'}^2}\right] \frac{\left[ \Delta _L^{sd}(Z')+\Delta _R^{sd}(Z')\right] }{\lambda _t}. \end{aligned}$$

We note that in addition to the $\Delta _{L,R}^{s d}(Z^\prime )$ couplings that will be constrained by the $\Delta S=2$ observables as discussed above, also the unknown coupling $\Delta _L^{\nu \nu }(Z^\prime )$ will be involved and consequently it will not be possible to make definite predictions for the branching ratios for these decays. However, it will be possible to learn something about the correlation between them. Evidently in the presence of a large $\Delta _L^{sd}(Z^\prime )$ coupling the present bounds on $K\rightarrow \pi \nu \bar{\nu }$ branching ratios can be avoided by choosing sufficiently low value of $\Delta _L^{\nu \bar{\nu }}(Z')$. In the case of scenario B, in which we ignore the $\Delta I=1/2$ rule issue and work only with left-handed $Z^\prime$-couplings, $\Delta _L^{sd}(Z^\prime )$ is forced to be small by $\varepsilon _K$ and $\Delta M_K$ constraints so that $\Delta _L^{\nu \bar{\nu }}(Z')$ can be chosen to be $\mathcal {O}(1)$.

### A toy model

There is an interesting aspect of the possible contribution of a $Z^\prime$ to the $\Delta I=1/2$ rule in the case in which the suppression factor $\delta$ does not vanish. One can relate the physics responsible for the missing piece in $\mathrm{Re} A_0$ to the one in $\varepsilon '/\varepsilon$, $\varepsilon _K$, $\Delta M_K$ and rare decays $K^+\rightarrow \pi ^+\nu \bar{\nu }$ and $K_{L}\rightarrow \pi ^0\nu \bar{\nu }$ and consequently obtain correlations between the related observables.

In order to illustrate this we consider a model for the $\Delta _R^{sd}(Z^\prime )$ coupling:

84 $$\begin{aligned} \begin{aligned}&\frac{\Delta _R^{sd}(Z^\prime )}{\Delta _L^{sd}(Z^\prime )}= -\frac{1}{2} R_Q(1+h R^2_Q),\\&R_Q\equiv \frac{\langle \hat{Q}_1^\text {VLL}((M_{Z^\prime })\rangle }{\langle \hat{Q}_1^\text {LR}((M_{Z^\prime })\rangle }\approx -0.01 \end{aligned} \end{aligned}$$

where $h=\mathcal {O}(1)$. This implies

85 $$\begin{aligned} \delta =\frac{1}{2} R_Q(1-4 h)^{1/2} +\mathcal {O}(R_Q^2), \end{aligned}$$

which shows that by a proper choice of the parameter $h$ one can suppress the NP contributions to $\Delta M_K$ to the level that it agrees with experiment.

In this model we find

86 $$\begin{aligned}&\varepsilon _K(Z^\prime )=-\frac{\kappa _\epsilon e^{i\varphi _\epsilon }}{\sqrt{2}(\Delta M_K)_\text {exp}}\frac{(\mathrm{Re}\Delta ^{sd}_L)(\mathrm{Im}\Delta ^{sd}_L)}{ M_{Z^\prime }^2}\nonumber \\&\qquad \qquad \qquad \times \langle \hat{Q}_1^\text {VLL}((M_{Z^\prime })\rangle \delta ^2\equiv \tilde{\varepsilon }_K(Z^\prime )e^{i\varphi _\epsilon },\end{aligned}$$

87 $$\begin{aligned}&\Delta M_K(Z^\prime )=\frac{(\mathrm{Re}\Delta ^{sd}_L)^2}{M_{Z^\prime }^2}\langle \hat{Q}_1^\text {VLL}((M_{Z^\prime })\rangle \delta ^2, \end{aligned}$$

where $\varphi _\epsilon = (43.51\pm 0.05)^\circ$ and $\kappa _\epsilon =0.94\pm 0.02$ [63, 64] takes into account that $\varphi _\epsilon \ne \tfrac{\pi }{4}$ and includes long distance effects in $\mathrm{Im}( \Gamma _{12})$ and $\mathrm{Im}(M_{12})$. The shift in the function $X(K)$ is in view of (84) given by

88 $$\begin{aligned} \Delta X(K)=\left[ \frac{\Delta _L^{\nu \bar{\nu }}(Z')}{g^2_\mathrm{SM}M_{Z'}^2}\right] \frac{\left[ \Delta _L^{sd}(Z')\right] }{\lambda _t}. \end{aligned}$$

While the $\delta$ is at this stage not fixed, it will be required to be non-vanishing in case SM predictions for $\varepsilon _K$ and $\Delta M_K$ will disagree with data once the parametric and hadronic uncertainties will be reduced. Moreover, independently of $\delta$, as long as it is non-vanishing these formulae together with (61) imply correlations

89 $$\begin{aligned}&\tilde{\varepsilon }_K(Z^\prime )= -\frac{\kappa _\epsilon }{\sqrt{2} r_{\Delta M} }\left[ \frac{{\mathrm{Im}\Delta _L^{sd}(Z^\prime )}}{{\mathrm{Re}\Delta _L^{sd}(Z^\prime )}}\right] ,\nonumber \\&r_{\Delta M}=\left[ \frac{(\Delta M_K)_\text {exp}}{\Delta M_K(Z^\prime )}\right] ,\end{aligned}$$

90 $$\begin{aligned}&\left( \frac{\varepsilon '}{\varepsilon }\right) _{Z^\prime }= \frac{3.5}{\kappa _\epsilon }\, \tilde{\varepsilon }_K(Z^\prime ) \left[ \frac{P\%}{20\,\%}\right] r_{\Delta M}. \end{aligned}$$

Already without a detailed numerical analysis we note the following general properties of this model:

- $\Delta M_K(Z^\prime )$ is strictly positive.
- As $P$ is also positive $\varepsilon '/\varepsilon$ and $\varepsilon _K$ are correlated with each other. Therefore this scenario can only work if the SM predictions for both observables are either below or above the data.
- The ratio of the NP contributions to $\varepsilon '/\varepsilon$ and $\varepsilon _K$ depends only on the product of $P$ and $r_{\Delta M}$.
- For $P=20\pm 10$, the NP contribution to $\varepsilon '/\varepsilon$ is predicted to be by an order of magnitude larger than in $\varepsilon _K$. This tells us that in order for the $Z^\prime$ contribution to be relevant for the $\Delta I=1/2$ rule and simultaneously be consistent with the data on $\varepsilon '/\varepsilon$, its contribution to $\varepsilon _K$ must be small implying that the SM value for $\varepsilon _K$ must be close to the data.

The correlations in (89) and (90) together with the condition (47) allow one to test this NP scenario in a straightforward manner as follows.

#### Step 1

We will set $r_{\Delta M}=4$, implying that $Z^\prime$ contributes $25\,\%$ of the measured value of $\Delta M_K$. In view of a large uncertainty in $\eta _{cc}$ and consequently in $(\Delta M_K)_\text {SM}$ this value is plausible and used here only to illustrate the general structure of what is going on. In this manner (90) gives us the relation between the NP contributions to $\varepsilon _K$ and $\varepsilon '/\varepsilon$. Note that this relation does not involve $B_6^{(1/2)}$ and only $P$. But the SM contribution to $\varepsilon '/\varepsilon$ involves explicitly $B_6^{(1/2)}$. Therefore the correlation of the resulting total $\varepsilon '/\varepsilon$ and $\varepsilon _K$ will depend on the values of $P$ and $B_6^{(1/2)}$ as well as CKM parameters. Note that to obtain these results it was not necessary to specify the value of $\Delta _L^{sd}(Z^\prime )$. But already this step will tell us which combination of $P$ and $B_6^{(1/2)}$ are simultaneously consistent with data on $\varepsilon '/\varepsilon$ and $\varepsilon _K$.

#### Step 2

In order to find $\Delta _L^{sd}(Z^\prime )$ and to test whether the results of Step 1 are consistent with the LHC data, we use condition (47). As we will see below LHC implies an upper bound on $\Delta _R^{qq}(Z^\prime )$ as a function of $M_{Z^\prime }$. For fixed $M_{Z^\prime }$ setting $\Delta _R^{qq}(Z^\prime )$ at a value consistent with this bound allows one to determine the minimal value of $\mathrm{Re} \Delta _L^{sd}(Z^\prime )$ as a function of $P$ and $B_6^{(1/2)}$. Combining finally these results in Sect. 5.2 with the bound on $\mathrm{Re} \Delta _L^{sd}(Z^\prime )$ from the LHC we will finally be able to find what are the maximal values of $P$ consistent with all available constraints and this will also restrict the values of $B_6^{(1/2)}$.

Having $\mathrm{Re} \Delta _L^{sd}(Z^\prime )$ as a function of $P$, $B_6^{(1/2)}$ and $\Delta _R^{qq}(Z^\prime )$, we can next use the relation (89) to calculate $\mathrm{Im} \Delta _L^{sd}(Z^\prime )$ as a function of $\tilde{\varepsilon }_K(Z^\prime )$. We will then find that only a certain range of the values of $\mathrm{Im} \Delta _L^{sd}(Z^\prime )$ is consistent with the data on $\varepsilon _K$ and $\varepsilon '/\varepsilon$ and that this range depends on $P$, $B_6^{(1/2)}$ and $\Delta _R^{qq}(Z^\prime )$.

#### Step 3

With this information on the allowed values of the coupling $\Delta _L^{sd}(Z^\prime )$ we can find the correlation between the branching ratios for $K^+\rightarrow \pi ^+\nu \bar{\nu }$ and $K_{L}\rightarrow \pi ^0\nu \bar{\nu }$ and the correlation between these two branching ratios and $\varepsilon '/\varepsilon$. To this end $\Delta _L^{\nu \nu }(Z^\prime )$ has to be suitably chosen.

### Scaling laws in the toy model

While the outcome of this procedure depends on the assumed value of $r_{\Delta M}$, the relations (89) and (90) allow one to find what happens for different values of $r_{\Delta M}$. To this end let us note the following facts.

The correlation between the NP contributions to $\varepsilon '/\varepsilon$ and $\varepsilon _K$ in (90) depends only on the product of $P$ and $r_{\Delta M}$. But one should remember that the full results for $\varepsilon '/\varepsilon$ and $\varepsilon _K$ that include also the SM contributions depend on the scenario $(a)$–$(f)$ for the CKM parameters considered in Sect. 5 and on $B_6^{(1/2)}$, explicitly present in the SM contribution. In a given CKM scenario there is specific room left for the NP contribution to $\varepsilon _K$, which restricts the allowed range for $\tilde{\varepsilon }_K$, which dependently on the scenario considered could be negative or positive. Thus dependently on $P$, $B_6^{(1/2)}$ and the CKM scenario $(a)$–$(f)$, one can adjust $r_{\Delta M}$ to satisfy simultaneously the data on $\varepsilon '/\varepsilon$ and $\varepsilon _K$. But as $r_{\Delta M}$ is predicted, in the model considered, to be positive, and long distance contributions, at least within the large $N$ approach [17], although small, are also predicted to be positive, $r_{\Delta M}$ cannot be too small.

Once the agreement on $\varepsilon '/\varepsilon$ and $\varepsilon _K$ is achieved it is crucial to verify whether the selected values of $P$ and $B_6^{(1/2)}$ are consistent with the LHC bounds on the couplings $\mathrm{Re}\Delta _L^{sd}(Z^\prime )$ and $\Delta _R^{q q}(Z^\prime )$, which are related to $P$ and $B_6^{(1/2)}$ through the relation (47). The numerical factor $-3.4$ in this equation valid for $Z^\prime$ is, as seen in (125), modified to $-2.4$ in the case of $G^\prime$. Otherwise the correlations between $\varepsilon '/\varepsilon$, $\varepsilon _K$ and $r_{\Delta M}$ given above are valid also for $G^\prime$, although the bounds on $\mathrm{Re}\Delta _L^{sd}(G^\prime )$ and $\Delta _R^{q q}(G^\prime )$ from the LHC differ from the $Z^\prime$ case, as we will see in Sect. 6.4.

In order to be prepared for the improvement of the LHC bounds in question we define

91 $$\begin{aligned}{}[\Delta _R^{q q}(Z^\prime )]_\text {eff}=\Delta _R^{q q}(Z^\prime )\left[ \frac{3\, \mathrm{TeV}}{M_{Z^\prime }}\right] ^2. \end{aligned}$$

**Fig. 1:**
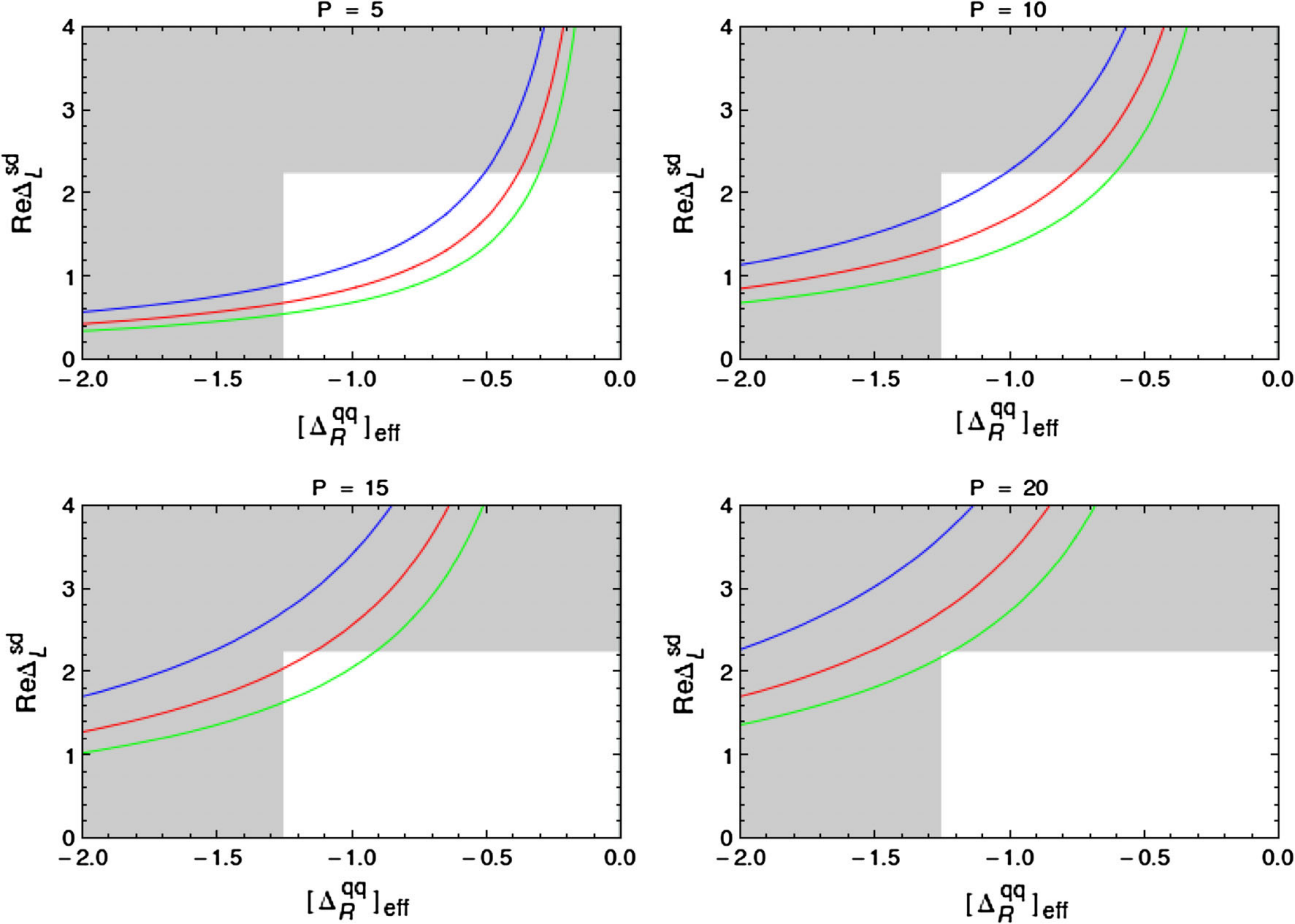
$\mathrm{Re} \Delta _L^{sd}(Z^\prime )$ versus $|[\Delta _R^{q q}(Z^\prime )]_\text {eff}|$ for $P = 5,~10,~15,~20$ and $B_6^{(1/2)}= 0.75$ (*blue*), $1.00$ (*red*) and $1.25$ (*green*). The *grey area* is basically excluded by the LHC. See Sect. 5.2

In the four panels in Fig. 1, corresponding to the four values of $P$ indicated in each of them, we plot $|[\Delta _R^{q q}(Z^\prime )]_\text {eff}|$ as a function of $\mathrm{Re} \Delta _L^{sd}(Z^\prime )$ for different values of $B_6^{(1/2)}$. For $M_{G^\prime }=M_{Z^\prime }$ the corresponding plot for $G^\prime$ can be obtained from Fig. 1 by either rescaling upwards all values of $P$ by a factor of $1.4$ or scaling down either $|[\Delta _R^{q q}(Z^\prime )]_\text {eff}|$ or $\mathrm{Re} \Delta _L^{sd}(Z^\prime )$ by the same factor. We will show such a plot in Sect. 6.4.

As we will discuss in Sect. 5.2 the values in the grey area corresponding to $|[\Delta _R^{q q}(Z^\prime )]_\text {eff}|\ge 1.25$ and $|\Delta _L^{sd}(Z^\prime )|\ge 2.3$ are basically ruled out by the LHC.^4^ We also note that, while for $P=5$ and $P=10$ and $B_6^{(1/2)}\ge 1.0$ the required values of $\mathrm{Re} \Delta _L^{sd}(Z^\prime )$ are in the ballpark of unity, for $P=20$ they are generally larger than 2, implying for $\mathrm{Re} \Delta _L^{sd}(Z^\prime )=2.3$

92 $$\begin{aligned} \alpha _L=\frac{[\mathrm{Re} \Delta _L^{sd}(Z^\prime )]^2}{4\pi }=0.42. \end{aligned}$$

As $\alpha _L$ is not small let us remark that in the case of a $U(1)$ gauge symmetry for even larger values of $\alpha _L$ it is difficult to avoid a Landau pole at higher scales. However, if only the coupling $\Delta _L^{sd}(Z^\prime )$ is large, a simple renormalisation group analysis shows that these scales are much larger than the LHC scales. Moreover, if $Z^\prime$ is associated with a non-abelian gauge symmetry that is asymptotically free, $\mathrm{Re} \Delta _L^{sd}(Z^\prime )$ could be even higher allowing one to reach values of $P$ as high as $25$–$30$. We will see in Sect. 6.4 that this is in fact the case for $G^\prime$.

In this context a rough estimate of the perturbativity upper bound on $\Delta _L^{sd}(Z^\prime )$ can be made by considering the loop expansion parameter^5^

93 $$\begin{aligned} L=N\frac{[\Delta _L^{sd}(Z^\prime )]^2}{16\pi ^2} \end{aligned}$$

where $N=3$ is the number of colours. For $\Delta _L^{sd}(Z^\prime )=2.5,~3.0,~3.5$ one has $L=0.12,~0.17,~0.23$, respectively, implying that using $\Delta _L^{sd}(Z^\prime )$ as large as $2.3$ can certainly be argued for.

### Strategy

This discussion and an independent numerical analysis using the general formulae presented above lead to the conclusion that for the goals of the present paper it is sufficient to consider only the following two scenarios for $Z^\prime$ couplings that satisfy the hierarchy (7).

#### Scenario A

This scenario is represented by our toy model constructed above. It provides a significant contribution to the $\Delta I=1/2$ rule without violating the constraints from the $\Delta F=2$ processes. Here, in addition to $\Delta _{L}^{sd}(Z^\prime )$ and $\Delta _R^{qq}(Z^\prime )$ of $\mathcal {O}(1)$, also a small $\Delta _{R}^{sd}(Z^\prime )$ satisfying (84) is required. Undoubtedly this scenario is fine-tuned but cannot be excluded at present. Moreover, it implies certain correlations between various observables and it is interesting to investigate them numerically. The three step procedure outlined above allows one to study transparently this scenario.

#### Scenario B

Among flavour-violating couplings only $\Delta _{L}^{sd}(Z^\prime )$ is non-vanishing or at all relevant. In this case only the SM operator contributes to $\varepsilon _K$ and $\Delta M_K$ and we deal with scenario LHS for flavour-violating couplings not allowing for the necessary shift in $\mathrm{Re}A_0$ due to the $\Delta M_K$ constraint but still providing interesting results for $\varepsilon '/\varepsilon$. Indeed only the QCD-penguin operator $Q_6$ contributes as in scenario A to the NP part in $K_L\rightarrow \pi \pi$ in an important manner. But $\mathrm{Re}A_0^\mathrm{NP}$ in this scenario is very small and there is no relevant correlation between the $\Delta I=1/2$ rule and the remaining observables. The novel part of our analysis in this scenario relative to our previous papers is the analysis of $\varepsilon '/\varepsilon$ and of its correlation with $K^+\rightarrow \pi ^+\nu \bar{\nu }$ and $K_{L}\rightarrow \pi ^0\nu \bar{\nu }$.

**Table 2:** Values of the experimental and theoretical quantities used as input parameters

$G_F = 1.16637(1)\times 10^{-5}\, \mathrm{GeV}^{-2}$	[1]	$M_W = 80.385(15) \, \mathrm{GeV}$	[1]
$\sin ^2\theta _W = 0.23116(13)$	[1]	$\alpha (M_Z) = 1/127.9$	[1]
$\alpha _s(M_Z)= 0.1185(6)$	[1]	$m_K= 497.614(24)\, \mathrm{MeV}$	[65]
$m_u(2\, \mathrm{GeV})=(2.1\pm 0.1)\, \mathrm{MeV}$	[50]	$m_\pi =135.0\, \mathrm{MeV}$	
$m_d(2\, \mathrm{GeV})=(4.68\pm 0.16)\, \mathrm{MeV}$	[50]	$F_\pi = 129.8\, \mathrm{MeV}$	
$m_s(2\, \mathrm{GeV})=(93.8\pm 2.4) \, \mathrm{MeV}$	[50]	$F_K = 156.1(11)\, \mathrm{MeV}$	[66]
$m_c(m_c) = (1.279\pm 0.013) \, \mathrm{GeV}$	[67]	$|V_{us}|=0.2252(9)$	[68]
$m_b(m_b)=4.19^{+0.18}_{-0.06}\, \mathrm{GeV}$	[1]	$|V^\text {incl.}_{ub}|=(4.41\pm 0.31)\times 10^{-3}$	[1]
$m_t(m_t) = 163(1)\, \mathrm{GeV}$	[66, 69]	$|V^\text {excl.}_{ub}|=(3.23\pm 0.31)\times 10^{-3}$	[1]
$\eta _{cc}=1.87(76)$	[70]	$|V_{cb}|=(40.9\pm 1.1)\times 10^{-3}$	[1]
$\eta _{tt}=0.5765(65)$	[71]	$\hat{B}_K= 0.75$	
$\eta _{ct}= 0.496(47)$	[72]	$\kappa _\epsilon =0.94(2)$	[63, 64]

## Numerical analysis

### Preliminaries

In order to proceed we have to describe how we treat parametric and hadronic uncertainties in the SM contributions, as this will determine the room left for NP contributions in the observables discussed by us.

First in order to simplify the numerical analysis we will set all parameters in Table 2, except for $|V_{ub}|$ and $|V_{cb}|$, at their central values. Concerning the latter two we will investigate six scenarios for them in order to stress the importance of their determination in the context of the search for NP through various observables. In order to bound the parameters of the model and to take hadronic and parametric uncertainties into account we will first only require that in scenario B the results for $\Delta M_K$ and $\varepsilon _K$ including the NP contributions satisfy

94 $$\begin{aligned}&0.75\le \frac{\Delta M_K}{(\Delta M_K)_\mathrm{SM}}\le 1.25,\nonumber \\&2.0\times 10^{-3}\le |\varepsilon _K|\le 2.5 \times 10^{-3}. \end{aligned}$$

However, it will be interesting to see what happens when the allowed range for $\varepsilon _K$ is reduced to the $3\sigma$ range around its experimental value. In scenario A, which is easier to handle numerically, we will see more explicitly what happens to $\Delta M_K$ and $\varepsilon _K$ and the latter $3\sigma$ range will be more relevant than the use of (94).

We will set $M_{Z^\prime }=3~\, \mathrm{TeV}$ as our nominal value. This is an appropriate value for being consistent with ATLAS and CMS experiments although as we will discuss below such a mass puts an upper bound on $\Delta _R^{qq}(Z')$. The scaling laws in [33] and our discussion in Sect. 4.4 allow us to translate our results to other values of $M_{Z^\prime }$. In particular when $\Delta _L^{sd}(Z')$ is bounded by $\Delta S=2$ observables, the NP effects in $\Delta F=1$ decrease with increasing $M_{Z^\prime }$. Therefore in order that NP plays a role in the $\Delta I=1/2$ rule and the involved couplings are in the perturbative regime, $M_{Z^\prime }$ should be smaller than $5~\, \mathrm{TeV}$ and consequently in the reach of the upgraded LHC.

Concerning the values of $\Delta _L^{sd}(Z')$ the numerical analyses in scenarios A and B differ in the following manner from each other:

- In scenario A, in which $\mathrm{Re}A_0$ plays an important role, we will use the three step procedure outlined in the previous section. In this manner we will find that $\Delta _L^{sd}(Z^\prime )\ge 1$ in order for $Z^\prime$ to play any role in the $\Delta I=1/2$ rule.
- In scenario B, we can proceed as in our previous papers by using the parametrisation
  95 $$\begin{aligned} \Delta _L^{sd}(Z')=-\tilde{s}_{12} e^{-i\delta _{12}}, \end{aligned}$$
  and searching for the allowed oases in the space $(\tilde{s}_{12},\delta _{12})$ that satisfy the constraints in (94) or the stronger $3\sigma$ constraint for $\varepsilon _K$. In this scenario $\Delta _L^{sd}(Z^\prime )$ will turn out to be very small. We will not show the results for these oases as they can be found in [26].

Having determined $\Delta _L^{sd}(Z')$ we can proceed to calculate the $\Delta F=1$ observables and study the correlations between them. Here additional uncertainties will come from $B_6^{(1/2)}$, which is hidden in the condition (47) so that it does not appear explicitly in the NP contributions but affects the SM contribution to $\varepsilon '/\varepsilon$. Also the $Z^\prime$ coupling to the neutrinos has to be fixed.

Finally the uncertainties due to the values of the CKM elements $|V_{cb}|$ and $|V_{ub}|$ have to be considered. These uncertainties are at first sight absent in the $Z^\prime$ contributions but affect the SM predictions for $\varepsilon _K$ and $\varepsilon '/\varepsilon$ and, consequently, indirectly also the $Z^\prime$ contributions through the size of the allowed range for $\Delta ^{sd}_{L}(Z^\prime )$ in both scenarios A and B. Indeed $\varepsilon '/\varepsilon$ and $K_{L}\rightarrow \pi ^0\nu \bar{\nu }$ depend in the SM on ${\mathrm{Im}\lambda _t}$, while $\varepsilon _K$ and $K^+\rightarrow \pi ^+\nu \bar{\nu }$ depend on both ${\mathrm{Im}\lambda _t}$ and ${\mathrm{Re}\lambda _t}$. Now within the accuracy of better than $0.5\,\%$ we have

96 $$\begin{aligned} {\mathrm{Im}\lambda _t}&= |V_{ub}||V_{cb}|\sin \gamma , \quad {\mathrm{Re}\lambda _t} = -{\mathrm{Im}\lambda _t}\cot (\beta -\beta _s)\nonumber \\ \end{aligned}$$

with $\gamma$ and $\beta$ being the well-known angles of the unitarity triangle and $-\beta _s\approx 1^\circ$ is the phase of $V_{ts}$ after the minus sign has been factored out. Consequently, within the SM not only $\varepsilon '/\varepsilon$ and $\varepsilon _K$ but also the branching ratios for $K^+\rightarrow \pi ^+\nu \bar{\nu }$ and $K_{L}\rightarrow \pi ^0\nu \bar{\nu }$ will depend sensitively on the chosen values for $|V_{cb}|$ and $|V_{ub}|$.

One should recall that the typical values for $|V_{ub}|$ and $|V_{cb}|$ extracted from *inclusive* decays are (see [73, 74] and references therein)^6^

97 $$\begin{aligned} |V_{ub}|&= 4.1\times 10^{-3}, \quad |V_{cb}|= 42.0\times 10^{-3}, \end{aligned}$$

while the typical values extracted from *exclusive* decays read [75, 76]

98 $$\begin{aligned} |V_{ub}|=3.2\times 10^{-3}, \qquad |V_{cb}|=39.0\times 10^{-3}. \end{aligned}$$

As the determinations of $|V_{ub}|$ and $|V_{cb}|$ are independent of each other, it will be instructive to consider the following scenarios for these elements:

99 $$\begin{aligned} (a)&\quad |V_{ub}|= 3.2\times 10^{-3}\quad |V_{cb}|= 39.0\times 10^{-3}\quad (\mathrm{purple)} \end{aligned}$$

100 $$\begin{aligned} (b)&\quad |V_{ub}|= 3.2\times 10^{-3}\quad |V_{cb}|= 42.0\times 10^{-3}\quad (\mathrm{cyan)}\end{aligned}$$

101 $$\begin{aligned} (c)&\quad |V_{ub}|= 4.1\times 10^{-3}\quad |V_{cb}|= 39.0\times 10^{-3}\quad (\mathrm{magenta)}\end{aligned}$$

102 $$\begin{aligned} (d)&\quad |V_{ub}|= 4.1\times 10^{-3}\quad |V_{cb}|= 42.0\times 10^{-3}\quad (\mathrm{yellow)}\end{aligned}$$

103 $$\begin{aligned} (e)&\quad |V_{ub}|= 3.7\times 10^{-3}\quad |V_{cb}|= 40.5\times 10^{-3}\quad (\mathrm{green)}\end{aligned}$$

104 $$\begin{aligned} (f)&\quad |V_{ub}|= 3.9 \times 10^{-3}\quad |V_{cb}|= 42.0\times 10^{-3} \quad (\mathrm{blue)} \end{aligned}$$

where we also included two additional scenarios, one for averaged values of $|V_{ub}|$ and $|V_{cb}|$ and the last one ($(f)$) particularly suited for the analysis of scenario A. We also give the colour coding for these scenarios used in the plots.

Concerning the parameter $\hat{B}_K$, which enters the evaluation of $\varepsilon _K$, the world average from lattice QCD is $\hat{B}_K=0.766\pm 0.010$ [50], very close to the strictly large $N$ limit value $\hat{B}_K=0.75$. On the other hand the recent calculation within the dual approach to QCD gives $\hat{B}_K=0.73\pm 0.02$ [17]. Moreover, the analysis in [77] indicates that in the absence of significant $1/N^2$ corrections to the leading large $N$ value one should have $\hat{B}_K\le 0.75$. It is an interesting question whether this result will be confirmed by future lattice calculations which have a better control over the uncertainties than is possible within the approach in [17, 77]. For the time being it is a very good approximation to set simply $\hat{B}_K=0.75$. Indeed compared to the present uncertainties from $|V_{cb}|$ and $|V_{ub}|$ in $\varepsilon _K$ proceeding in this manner is fully justified.

Concerning the value of $\gamma$ we will just set $\gamma =68^\circ$. This is close to central values from recent determinations [78–80] and varying $\gamma$ simultaneously with $|V_{cb}|$ and $|V_{ub}|$ would not improve our analysis.

As seen in Table 3 the six scenarios for the CKM parameters imply rather different values of ${\mathrm{Im}\lambda _t}$ and ${\mathrm{Re}\lambda _t}$ and consequently different values for various observables considered by us. This is seen in this table where we give SM values for $\varepsilon _K$, $\Delta M_K$, $\Delta M_s$, $\Delta M_d$, $S_{\psi K_S}$, $\varepsilon '/\varepsilon$, $\mathcal {B}(K_{L}\rightarrow \pi ^0\nu \bar{\nu })$ and $\mathcal {B}(K^+\rightarrow \pi ^+\nu \bar{\nu })$ together with their experimental values. To this end we have used the central values of the remaining parameters, relevant for the $B_{s,d}^0$ systems collected in [61]. For completeness we give also the values for $\overline{\mathcal {B}}(B_s\rightarrow \mu ^+\mu ^-)$ and ${\mathcal {B}}(B_d\rightarrow \mu ^+\mu ^-)$.

We would like to warn the reader that the SM values for various observables in Table 3 have been obtained directly by using CKM parameters from tree-level decays and consequently differ from SM results obtained usually from unitarity triangle fits that include constraints from processes in principle affected by NP.

We note that for a given choice of $|V_{ub}|$, $|V_{cb}|$ and $\gamma$ the SM predictions can differ sizably from the data but these departures are different for different scenarios:

- Only in scenario $(a)$ does $S_{\psi K_S}^\mathrm{SM}$ agree fully with the data. On the other hand in the remaining scenarios $Z^\prime$ contributions to $B_d^0$–$\bar{B}_d^0$ are required to bring the theory to agree with the data. But then also $\Delta M_s$ and $\Delta M_d$ have to receive new contributions, even in the case of scenario $(a)$. As in the models considered here $Z^\prime$ flavour-violating couplings involving $b$-quarks are not fixed, this can certainly be achieved. We refer to [26, 32] for details.
- On the other hand $\varepsilon _K$ is definitely below the experimental value in scenario $(a)$ but roughly consistent with experiment in other scenarios leaving still some room for NP contributions. In particular in scenarios $(d)$ and $(f)$ it is close to its experimental value.
- $\Delta M_K$ is as expected the same in all scenarios and roughly $10\,\%$ below its experimental value. But we should remember that the large uncertainty in $\eta _{cc}$ corresponds to $\pm 40\,\%$ uncertainty in $\Delta M_K$ and still sizable NP contributions are allowed.
- The dependence of $\mathcal {B}(K_{L}\rightarrow \pi ^0\nu \bar{\nu })$ on scenario considered is large but moderate in the case of $\mathcal {B}(K^+\rightarrow \pi ^+\nu \bar{\nu })$.
- We emphasise the strong dependence on $|V_{cb}|$ and consequently on $|V_{ts}|$ of the branching ratios $\overline{\mathcal {B}}(B_s\rightarrow \mu ^+\mu ^-)$ and ${\mathcal {B}}(B_d\rightarrow \mu ^+\mu ^-)$. For exclusive values of $|V_{cb}|$ both branching ratios are significantly lower than the official SM values [81] obtained using $|V_{cb}|=42.4\times 10^{-3}$.

In scenario B, where the constraint from $\Delta I=1/2$ is absent we will have more freedom in adjusting the NP parameters to improve in each of the scenarios $(a)$–$(f)$ the agreement of the theory with the data, but within scenario A we will find that only for certain scenarios of the CKM parameters it will be possible to fit the data.

**Fig. 2:**
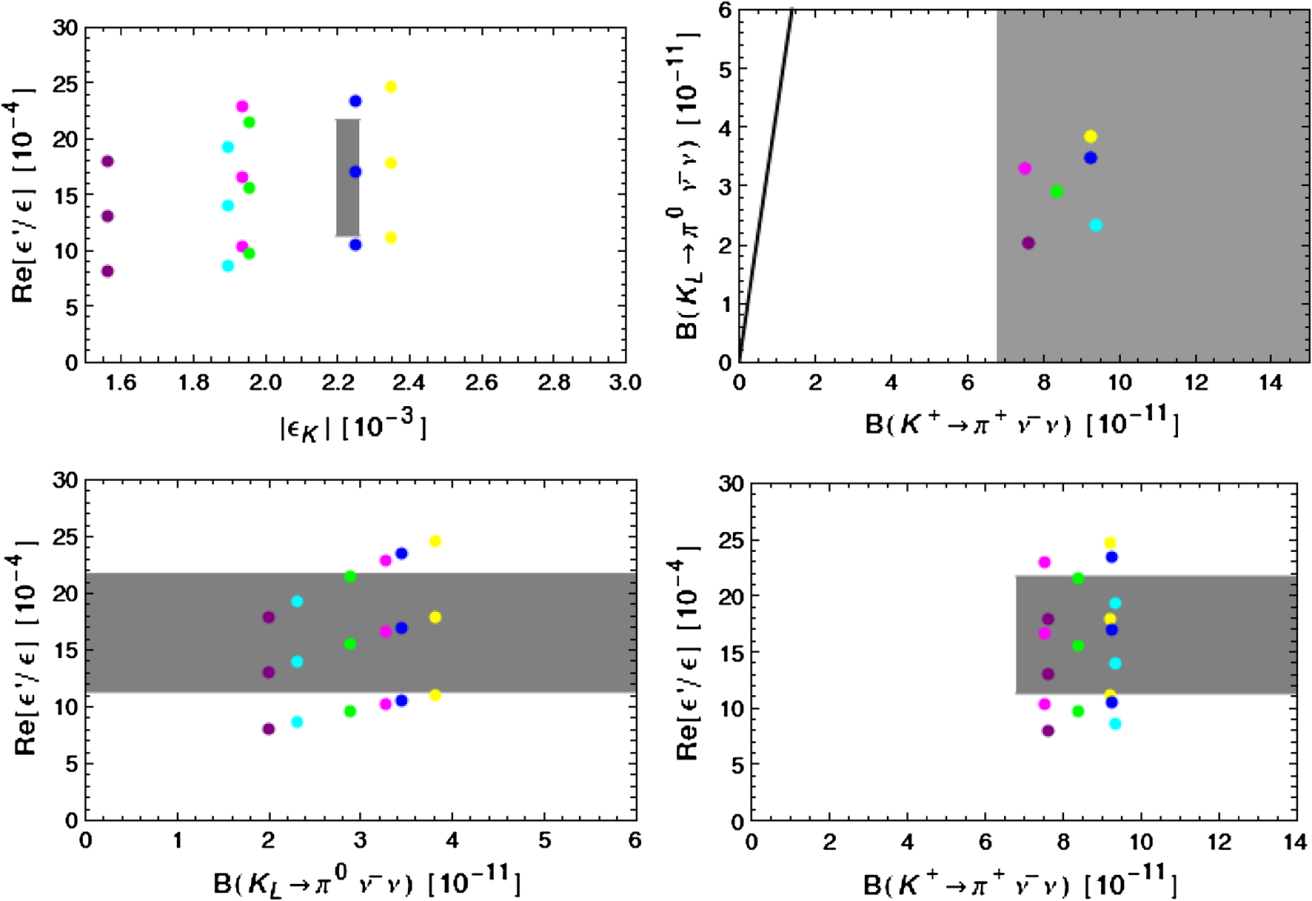
SM central values for $\varepsilon '/\varepsilon$, $\varepsilon _K$, $\mathcal {B}(K_{L}\rightarrow \pi ^0\nu \bar{\nu })$ and $\mathcal {B}(K_{L}\rightarrow \pi ^0\nu \bar{\nu })$ for scenarios (a) (*purple*), (b) (*cyan*), (c) (*magenta*), (d) (*yellow*), (e) (*green*) and (f) (*blue*) and different values of $B_6^{(1/2)} = 0.75,~1.00,~1.25$ corresponding to the increasing value of $\varepsilon '/\varepsilon$ for fixed colour. *Grey region* 2$\sigma$ experimental range of $\varepsilon '/\varepsilon$ and $3\sigma$ for $\varepsilon _K$

In Fig. 2 we summarise those results of Table 3 that will help us in following our numerical analysis in various NP scenarios presented by us. In particular, we observe in the lower left panel a strong correlation between $\varepsilon '/\varepsilon$ and $\mathcal {B}(K_{L}\rightarrow \pi ^0\nu \bar{\nu })$. Figure 2 shows graphically how important the determination of $|V_{ub}|$, $|V_{cb}|$ and $B_6^{(1/2)}$ in the indirect search for NP is. Let us hope that at the end of this decade there will be only a single point representing the SM in each of these four panels.

**Table 3:** Values of $\mathrm{Im}\lambda _t$, $\mathrm{Re}\lambda _t$ and of several observables within the SM for various scenarios of CKM elements as discussed in the text

	$$(a)$$	$$(b)$$	$$ (c)$$	$$ (d)$$	$$(e)$$	$$(f)$$	Data
$\mathrm{Im}\lambda _t\, [10^{-4}]$	$1.16$	$1.25$	$1.48$	$1.60$	$1.39$	$1.52$	$-$
$\mathrm{Re}\lambda _t\, [10^{-4}]$	$-2.90$	$-3.40$	$-2.76$	$-3.25$	$-3.07$	$-3.29$	$-$
$$S^\mathrm{SM}_{\psi K_S}$$	$$0.664$$	$$ 0.622$$	$$0.808$$	$$0.765$$	$$0.726$$	$$0.736$$	$$ 0.679(20)$$
$$\Delta M_s\,[\text {ps}^{-1}]$$	$$15.92$$	$$18.44$$	$$15.99$$	$$18.51$$	$$17.19$$	$$18.49 $$	$$17.69(8)$$
$$\Delta M_d\,[\text {ps}^{-1}]$$	$$ 0.47$$	$$0.54$$	$$0.47$$	$$0.54$$	$$0.50$$	$$0.54$$	$$0.510(4)$$
$$ \Delta M_K\,[10^{-3}\text {ps}^{-1}]$$	$$4.70$$	$$4.72 $$	$$4.70 $$	$$4.71$$	$$4.71$$	$$4.72$$	$$5.293(9)$$
$$|\varepsilon _K|\,[10^{-3}]$$	$$1.56$$	$$1.89$$	$$1.93$$	$$2.35$$	$$1.96$$	$$2.25 $$	$$2.228(11)$$
$$\varepsilon '/\varepsilon \,[10^{-4}](B_6^{(1/2)} = 0.75)$$	$$ 8.0$$	$$ 8.6$$	$$10.2$$	$$11.0$$	$$9.6$$	$$10.5 $$	$$16.5\pm 2.6$$
$\varepsilon '/\varepsilon \,[10^{-4}](B_6^{(1/2)} = 1.00)$	$12.9$	$13.9$	$16.5$	$17.8$	$15.5$	$16.9$	$16.5\pm 2.6$
$$\varepsilon '/\varepsilon \,[10^{-4}](B_6^{(1/2)} = 1.25)$$	$$17.8$$	$$19.2$$	$$22.8$$	$$24.6$$	$$21.4$$	$$23.4 $$	$$16.5\pm 2.6$$
$\mathcal {B}(K_L\rightarrow \pi ^0\nu \bar{\nu })\,[10^{-11}]$	$2.01$	$2.33$	$3.29$	$3.82$	$2.89$	$3.45$	$\le$ $2.6\times 10^{-8}$
$\mathcal {B}(K^+\rightarrow \pi ^+\nu \bar{\nu })\,[10^{-11}]$	$7.65$	$9.40$	$7.54$	$9.25$	$8.40$	$9.28$	$17.3^{+11.5}_{-10.5}$
$\overline{\mathcal {B}}(B_s\rightarrow \mu ^+\mu ^-)\,[10^{-9}]$	$3.00$	$3.47$	$3.01$	$3.48$	$3.23$	$3.48$	$2.9\pm 0.7$
$$\mathcal {B}(B_d\rightarrow \mu ^+\mu ^-)\,[10^{-10}]$$	$$ 0.94 $$	$$1.09$$	$$0.94$$	$$1.09$$	$$1.01$$	$$1.09$$	$$ 3.6^{+1.6}_{-1.4}$$

### LHC constraints

Finally, we should remember that $Z^\prime$ couplings to quarks can be bounded by collider data as obtained from LEP-II and the LHC. In the case of LEP-II all the bounds can be satisfied in our models by using sufficiently small leptonic couplings. However, in the case of $\Delta _R^{qq}$ and $\Delta _L^{sd}$ we have to check whether the values $\Delta _R^{qq}(Z^\prime )=\mathcal {O}(1)$ and $\Delta _L^{sd}(Z^\prime )=\mathcal {O}(1)$ necessary for a significant $Z^\prime$ contribution to $\mathrm{Re} A_0$ are allowed by the ATLAS and CMS outcome of the search for narrow resonances using the dijet mass spectrum in proton–proton collisions and by the effective operator bounds.

Bounds of this sort can be found in [40, 87–90] but the $Z^\prime$ models considered there have SM couplings or as in the case of [40] all diagonal couplings, both left-handed and right-handed, are flavour universal, which is not the case of our models in which the hierarchy (7) is assumed.

**Fig. 3:**
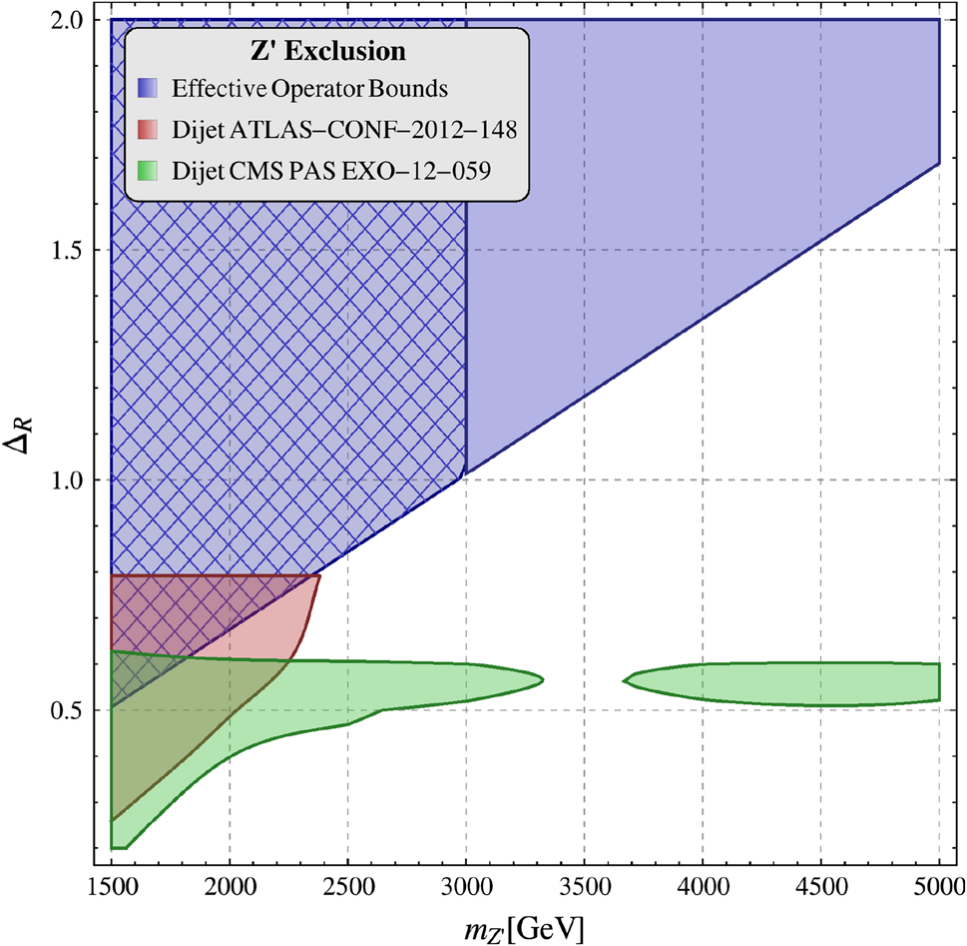
Exclusion limits for the $Z'$ in the mass-coupling plane, from various searches at the LHC as found in [82]. The *blue region* is excluded by effective operator limits studied by ATLAS [83] and CMS[84]. The *dashed surface* represents the region where the effective theory is not applicable, and the bounds here should be interpreted as a rough estimate. The *red* and *green* contours are excluded by dijet resonance searches by ATLAS [85] and CMS [86]. See additional comments in the text
- A second source of exclusion limits for $Z'$ boson couplings comes from the effective operator limits, in this case from four-quark operators studied by both ATLAS [83] and CMS [84]. As seen in Fig. 3 the upper bound on $|\Delta _R^{qq}(Z^\prime )|$ can be summarised by
  105 $$\begin{aligned} |\Delta _R^{qq}(Z^\prime )|\le 1.0\times \left[ \frac{M_{Z^\prime }}{3\, \mathrm{TeV}}\right] . \end{aligned}$$

For this reason a dedicated analysis of our toy model has been performed [82]^7^ using the most recent results from ATLAS and CMS. The result of this study is presented in Fig. 3 and can be briefly summarised as follows:

- The most up to date dijet searches from ATLAS [85] and CMS [86] allow one to put an upper bound on $|\Delta _R^{qq}(Z^\prime )|$ but only for $|\Delta _R^{qq}(Z^\prime )|\le 0.8$. As seen in Fig. 3 this maximal value is only allowed for $M_{Z^\prime }\ge 2.4\, \mathrm{TeV}$.

The following additional comments should be made in connection with the results in Fig. 3:

- The dijet limits are only effective if the width of the $Z'$ or $G'$ is below $15\,\%$ for ATLAS and $10\,\%$ for CMS.
- The lack of exclusion limits for CMS around $M_{Z'}=3.5$ TeV are the result of a fluctuation in the data and therefore their exclusion limits.
- It is important to note that the limits from effective operator constraints should not to be trusted when the centre of mass energy of the experiment is bigger than the mass of the particle, which is integrated out. For this analysis the effective centre of mass energy is $3\, \mathrm{TeV}$.

While dijets constraints would still allow for $[\Delta _R^{q q}(Z^\prime )]_\text {eff}$ $=1.25$ (see (91)) we will use for it $1.0$ so that our nominal values will be

106 $$\begin{aligned} \Delta _R^{qq}(Z^\prime )=-1.0, \qquad M_{Z^\prime }=3\, \mathrm{TeV}, \end{aligned}$$

consistent with the bound in (105). As seen in (47) the couplings $\Delta _R^{qq}(Z^\prime )$ and $\Delta _L^{sd}(Z^\prime )$ must have opposite signs in order to satisfy the $\Delta I=1/2$ constraint. On the basis of the present LHC data it is not possible to decide which of the two possible sign choices for these couplings is favoured by the collider data but this could be in principle possible in the future. The minus in $\Delta _R^{qq}(Z^\prime )$ is chosen here only to keep the coupling $\Delta _L^{sd}(Z^\prime )$ positive definite but presently the same results would be obtained with the other choice for signs of these two couplings.

As far as $\Delta _L^{sd}(Z^\prime )$ is concerned the derivation of corresponding bounds is more difficult, since the experimental collaborations do not provide constraints for flavoured four-quark interactions. However, there have been made efforts to obtain these from the current data [88, 91]. In particular the analysis of the $\Delta S=2$ operator in [91] turns out to be useful. With its help one finds the upper bound [82]

107 $$\begin{aligned} |\Delta _L^{sd}(Z^\prime )|\le 2.3 \left[ \frac{M_{Z^\prime }}{3\, \mathrm{TeV}}\right] . \end{aligned}$$

Now, as seen in Fig. 1 with (106), the values $P=20$–$30$ require $\mathrm{Re}\Delta _L^{sd}(Z^\prime )\approx 3$–$4$ dependently on the value of $B_6^{(1/2)}$. This would still be consistent with rough perturbativity bound $\mathrm{Re}\Delta _L^{sd}(Z^\prime )\le 4$ discussed by us in Sect. 4.4. However, the LHC bound in (107) seems to exclude this possibility, although a dedicated analysis of this bound including simultaneously left-handed and right-handed couplings would be required to put this bound on a firm footing. We hope to return to such an analysis in the future. For the time being we conclude that the maximal values of $P$ possible in this NP scenario are in the ballpark of $16$, which is roughly of the size of the SM QCD-penguin contribution.

Indeed, combining the bounds on the couplings of $Z^\prime$ and its mass and using the relation (47) we arrive at the upper bound

108 $$\begin{aligned} P\le 16\left[ \frac{B_6^{(1/2)}}{1.0}\right] , \qquad (Z^\prime ). \end{aligned}$$

This result is also seen in Fig. 1. In principle for $B_6^{(1/2)}$ significantly larger than unity one could increase the value of $P$ above 20, but as we will see soon this is not allowed when simultaneously the correlation between $\varepsilon '/\varepsilon$ and $\varepsilon _K$ is taken into account.

At this point it should be emphasised that the dashed surface in Fig. 3 has in fact not been completely excluded by ATLAS and CMS analyses and as an example $\Delta _R^{qq}(Z^\prime )=-1.5$ and $M_{Z^\prime }=2.5\, \mathrm{TeV}$, allowing $P$ to be as high as $30$, is still a valid point. While it is likely that a dedicated analysis of this model by ATLAS and CMS in this range of parameters would exclude the dashed surface completely, such an analysis has still to be done.

### Results

#### SM results for $\varepsilon '/\varepsilon$

We begin our presentation by discussing briefly the SM prediction for $\varepsilon '/\varepsilon$ given in Table 3 for different scenarios for CKM couplings and three values of $B_6^{(1/2)}$. We observe that for $B_6^{(1/2)}=1.00$, except for scenario $(a)$, the SM is in good agreement with the data but in view of the experimental error NP at the level of $\pm 20\,\%$ can still contribute. In the past when $B_8^{(3/2)}=1.0$ was used $\varepsilon '/\varepsilon$ for $B_6^{(1/2)}=1.0$ was below the data, but with the lattice result $B_8^{(3/2)}=0.65\pm 0.05$ [21] it looks like $B_6^{(1/2)}\approx 1.0$ is the favourite value within the SM. Except for scenario $(a)$ and $B_6^{(1/2)}=1.25$, for which SM gives values consistent with experiment, for the other two values of $B_6^{(1/2)}$ we get either visibly lower or visibly higher values of $\varepsilon '/\varepsilon$ than measured and some NP is required to fit the data.

### Scenario A

The question then arises whether simultaneous agreement with the data for ${\mathrm{Re}} A_0$, $\varepsilon _K$ and $\varepsilon '/\varepsilon$ can be obtained in the toy $Z^\prime$ model introduced by us.

**Fig. 4:**
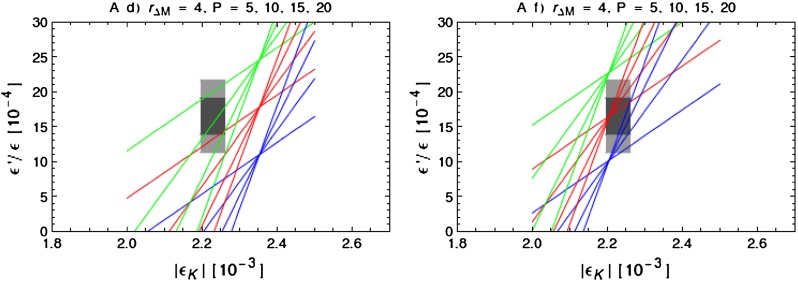
$\varepsilon '/\varepsilon$ versus $\varepsilon _K$ for scenario for scenario $(d)$ and $(f)$ for $r_{\Delta M} = 4$. *Light* (*dark*) *grey region*: experimental $2\sigma$($1\sigma$) range of $\varepsilon '/\varepsilon$ and $3\sigma$ range $2.195\times 10^{-3}\le |\varepsilon _K|\le 2.261\times 10^{-3}$. *Blue*, *red* and *green* stands for $B_6^{(1/2)} = 0.75,\,1.00,\,1.25$, respectively and for $P$ we use $5,\,10,\,15,\,20$ (the steeper the line, the larger $P$)

We use the three step procedure suited for this scenario that we outlined in the previous section. Investigating all six scenarios $(a)$–$(f)$ for $(|V_{cb}|,|V_{ub}|)$ we have found that only in scenarios $(d)$ and $(f)$ it is possible to obtain satisfactory agreement with the data on $\varepsilon '/\varepsilon$ and $\varepsilon _K$ for significant values of $P$. Indeed due to relation (90) NP in $\varepsilon _K$ must be small in order to keep $\varepsilon '/\varepsilon$ under control. As seen in Fig. 2 this is only the case in these two CKM scenarios. Yet, as seen in Fig. 4, even $(d)$ and $(f)$ scenarios can be distinguished by the correlation between $\varepsilon '/\varepsilon$ and $\varepsilon _K$ demonstrating again how important it is to determine precisely $|V_{cb}|$ and $|V_{ub}|$.

While, as seen in (90), the correlation between the NP contributions to $\varepsilon '/\varepsilon$ and $\varepsilon _K$ depends at fixed $r_{\Delta M}$ only on $P$, in the case of SM contributions it depends explicitly on $B_6^{(1/2)}$. Therefore we show in Fig. 4 the lines for $B_6^{(1/2)}=0.75,~1.00,~1.25$ using the colour coding

109 $$\begin{aligned} \begin{aligned} B_6^{(1/2)}&= 0.75 ~\text {(blue)},\quad B_6^{(1/2)}= 1.0~\text {(red)},\\ B_6^{(1/2)}&= 1.25~\text {(green)}. \end{aligned} \end{aligned}$$

The three lines carrying the same colour correspond to four values of $P=5,~10,~15,~20$. With increasing $P$ the lines become steeper. The dark (light) grey region corresponds to the $1(2)\sigma$ experimental range for $\varepsilon '/\varepsilon$ and $3\sigma$ range for $\varepsilon _K$.

Beginning with scenario $(d)$ we observe that only the following combinations of $P$ and $B_6^{(1/2)}$ are consistent with this range:

- For $B_6^{(1/2)}=1.25$ only $P=5,~10,~15$ are allowed when $1\sigma$ range for $\varepsilon '/\varepsilon$ is considered. At $2\sigma$ also $P=20$ is allowed. Larger values of $P$ are only possible for $B_6^{(1/2)}>1.25$. We conclude therefore that for $B_6^{(1/2)}=1.25$ we find the upper bound $P\le 20$.
- For $B_6^{(1/2)}=1.00$ the corresponding upper bound amounts to $P\le 10$.
- For $B_6^{(1/2)}=0.75$ even for $P=5$ one cannot obtain simultaneous agreement with the data on $\varepsilon '/\varepsilon$ and $\varepsilon _K$.

A rather different pattern is found for scenario $(f)$:

- For $B_6^{(1/2)}=1.25$ the values $P=5,~10,~15,~20$ are not allowed even at $2\sigma$ range for $\varepsilon '/\varepsilon$ but decreasing slightly $B_6^{(1/2)}$ would allow values $P\ge 20$.
- On the other hand, in the case of $B_6^{(1/2)}=1.00$ there is basically no restriction on $P$ from this correlation simply because in this scenario the NP contributions to $\epsilon _K$ are small (see Fig. 2). In fact in this case values of $P$ as high as $30$ would be allowed. While such values are not possible in the case of $Z^\prime$ due to LHC constraint in (108) we will see that they are allowed in the case of $G^\prime$.
- Similar situation is found for $B_6^{(1/2)}=0.75$ although here at $1\sigma$ for $\varepsilon '/\varepsilon$ one finds the bound $P\ge 10$.

We conclude therefore that in view of the fact that the NP effects in $\varepsilon '/\varepsilon$ in our toy model are by an order of magnitude larger than in $\varepsilon _K$, scenario $(f)$ is particularly suited for allowing large values of $P$ as it avoids strong constraints from $\varepsilon '/\varepsilon$ and $\varepsilon _K$. In scenario $(d)$ independently of the LHC we find $P<20$. While in the case of $Z^\prime$ model at hand this virtue of scenario $(f)$ cannot be fully used because of the LHC constraint (108) we will see in the next section that it plays a role in the case of $G^\prime$ model. These findings are interesting as they imply that only for the inclusive determinations of $|V_{ub}|$ and $|V_{cb}|$ $Z^\prime$ has a chance to contribute in a significant manner to the $\Delta I=1/2$ rule. This assumes the absence of other mechanisms at work which otherwise could help in this case if the exclusive determinations of these CKM parameters would turn out to be true.

**Fig. 5:**
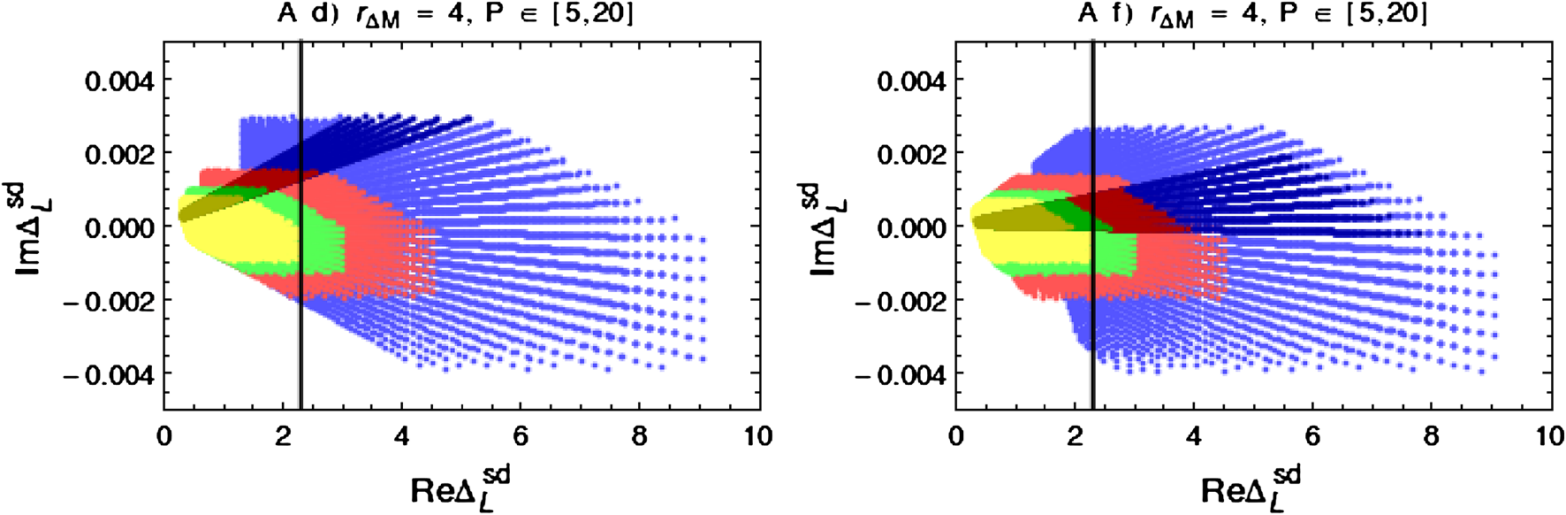
Here we show the allowed values of $\mathrm{Re}\Delta _L^{sd}$ and $\mathrm{Im}\Delta _L^{sd}$ in scenario A $(d)$ and $(f)$ for $\Delta _R^{qq} = -0.5$ (*blue*), $-1$ (*red*), $-1.5$ (*green*) and $-2$ (*yellow*). We varied $P\in [5,\,20]$ and $B_6^{(1/2)}\in [0.75,1.25]$ and took only those $(B_6^{(1/2)},P)$ combinations that fulfill the constraints on $\varepsilon '/\varepsilon$ ($2\sigma$) and $\varepsilon _K$ (*darker colours* $3\sigma$ and *lighter colours* $2.0\times 10^{-3}\le |\varepsilon _K|\le 2.5\times 10^{-3}$). The *vertical black line* indicates the LHC bound in (107)

In Fig. 5 we show with darker colours the allowed values of $\mathrm{Re}\Delta _L^{sd}$ and $\mathrm{Im}\Delta _L^{sd}$ in scenario A for CKM values $(d)$ and $(f)$ that correspond to the values of $P$ and $B_6^{(1/2)}$ selected by the light grey region in Fig. 4. In lighter colours we show the allowed values of $\mathrm{Re}\Delta _L^{sd}$ and $\mathrm{Im}\Delta _L^{sd}$ using (94) as constraint for $\varepsilon _K$. As for $M_{Z^\prime }=3\, \mathrm{TeV}$ only values $|\Delta _R^{qq}|\le 1.0$ are allowed by the LHC bound in (105), the *green* and *yellow* ranges are ruled out, but we show them anyway, as this demonstrates the power of the LHC in constraining our model. Among the remaining areas the *red* one is favoured as it corresponds to smaller values of $\mathrm{Re}\Delta _L^{sd}$ for a given $P$ and this is the reason why $\Delta _R^{qq}=-1.0$ has been chosen as nominal value for this coupling. This feature is not clearly seen in this figure where we varied $P$ but this is evident from plots in Fig. 1. The vertical black line shows the LHC bound in (107). Only values on the left of this line are allowed.

We have investigated the correlation between $\mathcal {B}(K_{L}\rightarrow \pi ^0\nu \bar{\nu })$ and $\mathcal {B}(K^+\rightarrow \pi ^+\nu \bar{\nu })$ for scenarios $(d)$ and $(f)$ finding the following pattern that follows from the fact that in scenario A, as can be seen in Fig. 5, $\mathrm{Re} \Delta _L^{sd}(Z^\prime )=\mathcal {O}(1)$. In view of this, the neutrino coupling $\Delta _L^{\nu \nu }(Z^\prime )$ must be sufficiently small in order to be consistent with the data on $\mathcal {B}(K^+\rightarrow \pi ^+\nu \bar{\nu })$. But as seen in Fig. 5 $\mathrm{Im} \Delta _L^{sd}(Z^\prime )$ is required to be small in order to satisfy the data on $\varepsilon '/\varepsilon$ and $\varepsilon _K$. The smallness of both $\Delta _L^{\nu \nu }(Z^\prime )$ and $\mathrm{Im} \Delta _L^{sd}(Z^\prime )$ implies in this scenario negligible NP contributions to $\mathcal {B}(K_{L}\rightarrow \pi ^0\nu \bar{\nu })$. Thus the main message from this exercise is that $\mathcal {B}(K_{L}\rightarrow \pi ^0\nu \bar{\nu })$ remains SM-like, while $\mathcal {B}(K^+\rightarrow \pi ^+\nu \bar{\nu })$ can be modified but this modification depends on the size of the unknown coupling $\Delta _L^{\nu \nu }(Z^\prime )$ and changing its sign one can obtain both suppression or enhancement of $\mathcal {B}(K^+\rightarrow \pi ^+\nu \bar{\nu })$ relative to the SM value. For $\Delta _L^{\nu \nu }(Z^\prime )$ in the ballpark of $5\times 10^{-4}$ significant enhancements or suppressions can be obtained. In view of this simple pattern and low predictive power we refrain from showing any plots.

Yet, the requirement of strongly suppressed leptonic couplings implies that unless $\Delta _{L,R}^{sb}(Z^\prime )$ and $\Delta _{L,R}^{db}(Z^\prime )$ are sizable, in scenario A NP contributions to rare $B_{s,d}$ decays with neutrinos and charged leptons in the final state are predicted to be small. On the other hand these effects could be sufficiently large in $\Delta B=2$ processes to cure SM problems in scenarios $d$ and $f$ seen in Table 3.

While for a fixed value of $\Delta _L^{\nu \nu }(Z^\prime )$ there exist correlations between $\varepsilon '/\varepsilon$ and $\mathcal {B}(K^+\rightarrow \pi ^+\nu \bar{\nu })$ such correlations are more interesting in the case of scenario B, which we will discuss next.

### Scenario B

Here we proceed as in [26] except that we use scenarios $(a)$–$(f)$ for $(|V_{cb}|,|V_{ub}|)$ and also present results for $\varepsilon '/\varepsilon$. To this end we use colour coding for these scenarios in (99)–(104) and the one for $B_6^{(1/2)}$ in (109) and set

110 $$\begin{aligned} \Delta _R^{qq}(Z^\prime )=0.5,\,1.0, \qquad \Delta _L^{\nu \nu }(Z^\prime )=0.5 \, \end{aligned}$$

with darker (lighter) colours representing $\Delta _R^{qq}(Z^\prime )\!=\!1.0(0.5)$. These values of $\Delta _R^{qq}(Z^\prime )$ satisfy the LHC bounds. The neutrino coupling can be chosen as in our previous papers because the coupling $\Delta _L^{sd}(Z^\prime )$ will be bounded by $\Delta M_K$ and $\varepsilon _K$ to be very small and this choice is useful as it allows one to see the impact of the $\varepsilon '/\varepsilon$ constraint on our results for the rare decays $K^+\rightarrow \pi ^+\nu \bar{\nu }$ and $K_{L}\rightarrow \pi ^0\nu \bar{\nu }$ obtained in [26] without this constraint.

**Fig. 6:**
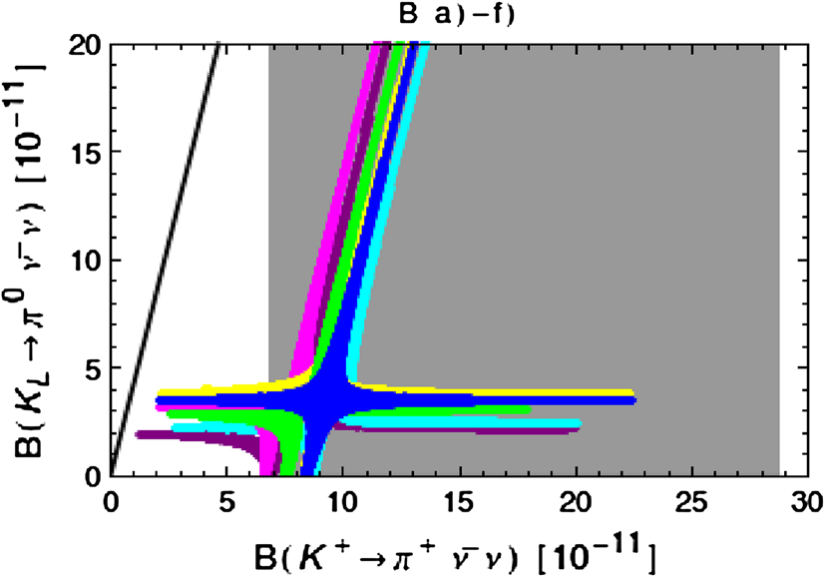
$\mathcal {B}(K_{L}\rightarrow \pi ^0\nu \bar{\nu })$ versus $\mathcal {B}(K^+\rightarrow \pi ^+\nu \bar{\nu })$ for scenario (a) (*purple*), (b) (*cyan*), (c) (*magenta*), (d) (*yellow*), (e) (*green*) and (f) (*blue*). *Grey region*: experimental range of $\mathcal {B}(K^+\rightarrow \pi ^+\nu \bar{\nu })$. The *black line* corresponds to the Grossman–Nir bound

**Fig. 7:**
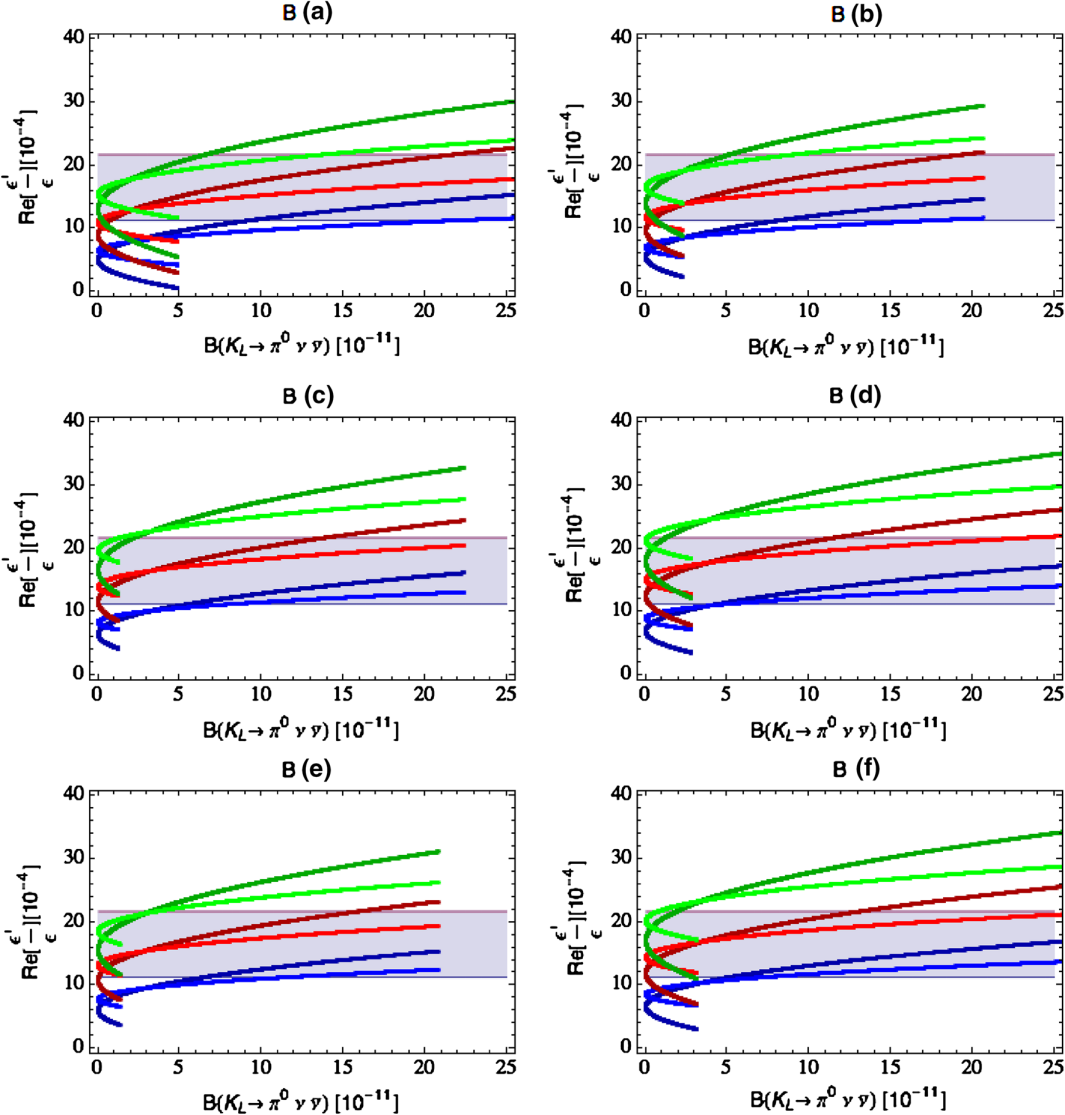
$\varepsilon '/\varepsilon$ versus $\mathcal {B}(K_{L}\rightarrow \pi ^0\nu \bar{\nu })$ for scenario $(a)$–$(f)$ and different values of $B_6^{(1/2)} = 0.75$ (*blue*), $B_6^{(1/2)} = 1.00$ (*red*), $B_6^{(1/2)} = 1.25$ (*green*) and $\Delta _R^{qq}(Z^\prime )=1.0(0.5)$ for *darker* (*lighter*) colours. *Grey region* 2$\sigma$ experimental range of $\varepsilon '/\varepsilon$

**Fig. 8:**
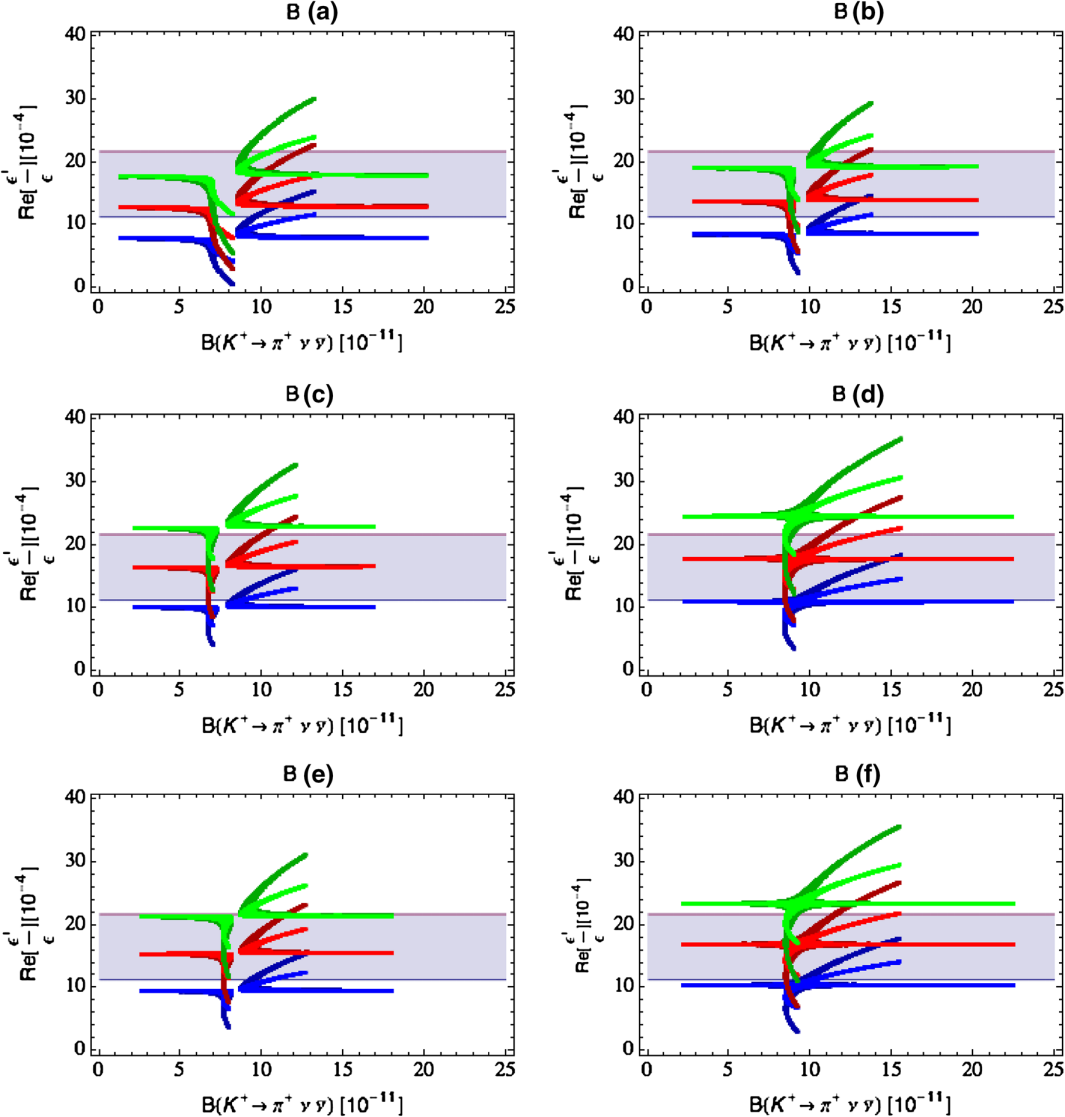
$\varepsilon '/\varepsilon$ versus $\mathcal {B}(K^+\rightarrow \pi ^+\nu \bar{\nu })$ for scenario $(a)$–$(f)$ and different values of $B_6^{(1/2)} = 0.75$ (*blue*), $B_6^{(1/2)} = 1.00$ (*red*), $B_6^{(1/2)} = 1.25$ (*green*) and $\Delta _R^{qq}(Z^\prime )=1.0(0.5)$ for *darker* (*lighter*) colours. *Grey region* 2$\sigma$ experimental range of $\varepsilon '/\varepsilon$

We find that due to the absence of the constraint from the $\Delta I=1/2$ rule in all six scenarios for $(|V_{cb}|,|V_{ub}|)$ agreement with the data on $\varepsilon _K$ and $\varepsilon '/\varepsilon$ can be obtained. In Fig. 6 we show the correlation between $\mathcal {B}(K_{L}\rightarrow \pi ^0\nu \bar{\nu })$ and $\mathcal {B}(K^+\rightarrow \pi ^+\nu \bar{\nu })$ for the six scenarios $(a)$–$(f)$ for $(|V_{cb}|,|V_{ub}|)$. In Figs. 7 and 8 we show correlations of $\varepsilon '/\varepsilon$ with $\mathcal {B}(K_{L}\rightarrow \pi ^0\nu \bar{\nu })$ and $\mathcal {B}(K^+\rightarrow \pi ^+\nu \bar{\nu })$, respectively.

We make the following observations:

- The plot in Fig. 6 is familiar from other NP scenarios. $\mathcal {B}(K_{L}\rightarrow \pi ^0\nu \bar{\nu })$ can be strongly enhanced on one of the branches and then $\mathcal {B}(K^+\rightarrow \pi ^+\nu \bar{\nu })$ is also enhanced. But $\mathcal {B}(K^+\rightarrow \pi ^+\nu \bar{\nu })$ can also be enhanced without modifying $\mathcal {B}(K_{L}\rightarrow \pi ^0\nu \bar{\nu })$. The last feature is not possible within the SM and any model with minimal flavour violation in which these two branching ratios are strongly correlated.
- As seen in Fig. 7, except for the smallest values of $\mathcal {B}(K_{L}\rightarrow \pi ^0\nu \bar{\nu })$, where this branching ratio is below the SM predictions, in each scenario there is a strong correlation between $\varepsilon '/\varepsilon$ and this branching ratio so that for fixed $B_6^{(1/2)}$ the increase of $\varepsilon '/\varepsilon$ uniquely implies the increase of $\mathcal {B}(K_{L}\rightarrow \pi ^0\nu \bar{\nu })$. In this case, as seen in Fig. 6, also $\mathcal {B}(K^+\rightarrow \pi ^+\nu \bar{\nu })$ increases so that we have actually a triple correlation.
- We note that even a small increase of $\varepsilon '/\varepsilon$ for fixed values of $B_6^{(1/2)}$ implies a strong increase of $\mathcal {B}(K_{L}\rightarrow \pi ^0\nu \bar{\nu })$. But this hierarchy applies only for $\Delta _R^{qq}(Z^\prime )$ and $\Delta _L^{\nu \nu }(Z^\prime )$ being of the same order as assumed in (110). Introducing a hierarchy in these couplings would change the effects in favour of $\varepsilon '/\varepsilon$ or $\mathcal {B}(K_{L}\rightarrow \pi ^0\nu \bar{\nu })$ relative to the results presented by us. In the case of $Z$ boson with FCNCs analysed in Sect. 7, where all diagonal couplings are fixed, definite results for this correlation will be obtained.
- Values of $B_6^{(1/2)}=1.25$ are disfavoured for scenarios $(c)$–$(f)$ unless $\mathcal {B}(K_{L}\rightarrow \pi ^0\nu \bar{\nu })$ is suppressed with respect to the SM value.
- For $B_6^{(1/2)}=1.0$ the branching ratio $\mathcal {B}(K_{L}\rightarrow \pi ^0\nu \bar{\nu })$ can reach values as high as $10^{-10}$ but in view of the experimental error in $\varepsilon '/\varepsilon$ this is not required by $\varepsilon '/\varepsilon$.
- For $B_6^{(1/2)}=0.75$ SM prediction for $\varepsilon '/\varepsilon$ is in all scenarios $(a)$–$(f)$ visibly below the data and curing this problem with $Z^\prime$ exchange enhances $\mathcal {B}(K_{L}\rightarrow \pi ^0\nu \bar{\nu })$ typically above $1.5\times 10^{-10}$.
- The main message from these plots is that values of $\mathcal {B}(K_{L}\rightarrow \pi ^0\nu \bar{\nu })$ as large as several $10^{-10}$ are not possible when the $\varepsilon '/\varepsilon$ constraint is taken into account unless the coupling $\Delta _R^{qq}(Z^\prime )$ is chosen to be much smaller than assumed by us.
- The correlation between $\varepsilon '/\varepsilon$ and $\mathcal {B}(K^+\rightarrow \pi ^+\nu \bar{\nu })$ is more involved as here also real part of $\Delta _L^{sd}(Z^\prime )$ plays a role. In particular we observe that $\mathcal {B}(K^+\rightarrow \pi ^+\nu \bar{\nu })$ can increase without affecting $\varepsilon '/\varepsilon$ at all. But then it is bounded from above by $K_L\rightarrow \mu ^+\mu ^-$, although this bound depends on the value of the $Z^\prime$ axial-vector coupling to muons, which is not specified here. If this coupling equals $\Delta _L^{\nu \nu }(Z^\prime )$ then as seen in Fig. 10 in [26] values of $\mathcal {B}(K^+\rightarrow \pi ^+\nu \bar{\nu })$ above $15\times 10^{-11}$ are excluded.

We emphasise that the correlation between $\varepsilon '/\varepsilon$ and the branching ratio $\mathcal {B}(K_{L}\rightarrow \pi ^0\nu \bar{\nu })$ shown in Figs. 7 and 8 differs markedly from many other NP scenarios, in particular LHT [46] and SM with four generations [92], where $\varepsilon '/\varepsilon$ was modified by electroweak penguin contributions. There, the increase of $\mathcal {B}(K_{L}\rightarrow \pi ^0\nu \bar{\nu })$ implied the decrease of $\varepsilon '/\varepsilon$ and only the values of $B_6^{(1/2)}$ significantly larger than unity allowed large enhancements of $\mathcal {B}(K_{L}\rightarrow \pi ^0\nu \bar{\nu })$. However, the correlations in Figs. 7 and 8 are valid for the assumed $\Delta _R^{qq}(Z^\prime )$. For the opposite sign of $\Delta _R^{qq}(Z^\prime )$ the values of $\varepsilon '/\varepsilon$ are flipped along the horizontal “central” line without the change in the branching ratios which do not depend on this coupling. Similarly, flipping the sign of $\Delta _L^{\nu \nu }(Z^\prime )$ would change the correlation between $\varepsilon '/\varepsilon$ and $\mathcal {B}(K_{L}\rightarrow \pi ^0\nu \bar{\nu })$ into anticorrelation.

### The primed scenarios and the $\Delta I=1/2$ rule

Clearly the solution for the missing piece in $\mathrm{Re} A_0$ can also be obtained by choosing $\Delta _R^{sd}(Z^\prime )$ and $\Delta _L^{qq}(Z^\prime )$ to be $\mathcal {O}(1)$ instead of $\Delta _L^{sd}(Z^\prime )$ and $\Delta _R^{qq}(Z^\prime )$, respectively. Interchanging $L$ and $R$ in the hierarchies (7) would then lead from the point of view of low energy flavour-violating processes to the same conclusions, which can be understood as follows.

In this primed scenario the operator $Q_6^\prime$ replaces $Q_6$ and as the matrix element $\langle Q_6^\prime \rangle _0$ differs by the sign from $\langle Q_6\rangle _0$, the $\Delta I=1/2$ rule requires the product $\Delta _R^{sd}(Z^\prime )\times \Delta _L^{qq}(Z^\prime )$ to be positive. Choosing then positive $\Delta _L^{qq}(Z^\prime )$ instead of a negative $\Delta _R^{qq}(Z^\prime )$ in scenario A our results for $\varepsilon '/\varepsilon$ and $\mathrm{Re} A_0$ remain unchanged as also the $\Delta S=2$ analysis remains unchanged. Similarly our analysis of $K^+\rightarrow \pi ^+\nu \bar{\nu }$ and $K_{L}\rightarrow \pi ^0\nu \bar{\nu }$ is not modified as these decays are insensitive to $\gamma _5$. The only change takes place in $K_L\rightarrow \mu ^+\mu ^-$ where for a fixed muon coupling the NP contribution has an opposite sign to the scenarios considered by us. But this change can be compensated by a flip of the sign of the muon coupling, which without a concrete model is not fixed.

On the other hand the difference between primed and unprimed scenarios could possibly be present in other processes, like the ones studied at the LHC, in which the constraints on the couplings could depend on whether the bounds on a negative product $\Delta _L^{sd}(Z^\prime )\times \Delta _R^{qq}(Z^\prime )$ or a positive product $\Delta _R^{sd}(Z^\prime )\times \Delta _L^{qq}(Z^\prime )$ are more favourable for the $\Delta I=1/2$ rule. However, presently, as discussed above, only separate bounds on the couplings involved and not their products are available. Whether the future bounds on these products will improve the situation of the $\Delta I=1/2$ rule remains to be seen.

## Coloured neutral gauge bosons $G^\prime$

### Modified initial conditions

In various NP scenarios neutral gauge bosons with colour ($G^\prime$) are present. One of the prominent examples of this type is that with Kaluza–Klein gluons in the Randal–Sundrum scenarios that belong to the adjoint representation of the colour $SU(3)_c$. In what follows we will assume that these gauge bosons carry a common mass $M_{G^\prime }$ and being in the octet representation of $SU(3)_c$ couple to fermions in the same manner as gluons do. However, we will allow for different values of their left-handed and right-handed couplings. Therefore up to the colour matrix $t^a$, the couplings to quarks will be again parametrised by

111 $$\begin{aligned}&\Delta _L^{s d}(G^\prime ),\qquad \Delta _R^{s d}(G^\prime ), \quad \Delta _L^{q q}(G^\prime ),\qquad \Delta _R^{qq}(G^\prime ) \end{aligned}$$

and the hierarchy in (7) will be imposed.

Calculating then the tree-diagrams with $G^\prime$ gauge boson exchanges and expressing the result in terms of the operators encountered in the previous sections we find that the initial conditions at $\mu =M_{G^\prime }$ are modified.

The new initial conditions for the operators entering $K\rightarrow \pi \pi$ now read at LO

112 $$\begin{aligned} \begin{aligned} C_3(M_{G^\prime })&= \left[ -\frac{1}{6}\right] \frac{\Delta _L^{s d}(G^\prime )\Delta _L^{q q}(G^\prime )}{4 M^2_{G^\prime }},\\ C_3^\prime (M_{G^\prime })&= \left[ -\frac{1}{6}\right] \frac{\Delta _R^{s d}(G^\prime )\Delta _R^{q q}(G^\prime )}{4 M^2_{G^\prime }}, \end{aligned} \end{aligned}$$

113 $$\begin{aligned} \begin{aligned} C_4(M_{G^\prime })&= \left[ \frac{1}{2}\right] \frac{\Delta _L^{s d}(G^\prime )\Delta _L^{q q}(G^\prime )}{4 M^2_{G^\prime }},\\ C_4^\prime (M_{G^\prime })&= \left[ \frac{1}{2}\right] \frac{\Delta _R^{s d}(G^\prime )\Delta _R^{q q}(G^\prime )}{4 M^2_{G^\prime }}, \end{aligned} \end{aligned}$$

114 $$\begin{aligned} \begin{aligned} C_5(M_{G^\prime })&= \left[ -\frac{1}{6}\right] \frac{\Delta _L^{s d}(G^\prime )\Delta _R^{q q}(G^\prime )}{4 M^2_{G^\prime }},\\ C_5^\prime (M_{G^\prime })&= \left[ -\frac{1}{6}\right] \frac{\Delta _R^{s d}(G^\prime )\Delta _L^{q q}(G^\prime )}{4 M^2_{G^\prime }}, \end{aligned} \end{aligned}$$

115 $$\begin{aligned} \begin{aligned} C_6(M_{G^\prime })&= \left[ \frac{1}{2}\right] \frac{\Delta _L^{s d}(G^\prime )\Delta _R^{q q}(G^\prime )}{4 M^2_{G^\prime }},\\ C_6^\prime (M_{G^\prime })&= \left[ \frac{1}{2}\right] \frac{\Delta _R^{s d}(G^\prime )\Delta _L^{q q}(G^\prime )}{4 M^2_{G^\prime }}. \end{aligned} \end{aligned}$$

Again due to the hierarchy in (7) the contributions of primed operators can be neglected. Moreover, due the non-vanishing value of $C_6(M_{G^\prime })$ the dominance of the operator $Q_6$ is this time even more pronounced than in the case of a colourless $Z^\prime$. Indeed we find now

116 $$\begin{aligned} \left[ \begin{array}{c} C_5(m_c) \\ C_6(m_c) \end{array}\right]&= \left[ \begin{array}{cc} 0.86 &{}\quad 0.19\\ 1.13 &{}\quad 3.60 \end{array}\right] \left[ \begin{array}{c} -1/6 \\ 1/2 \end{array}\right] \nonumber \\&\times \frac{\Delta _L^{s d}(G^\prime )\Delta _R^{q q}(G^\prime )}{4 M^2_{G^\prime }}. \end{aligned}$$

Consequently

117 $$\begin{aligned} \begin{aligned} C_5(m_c)&= -0.05 \frac{\Delta _L^{s d}(G^\prime )\Delta _R^{q q}(G^\prime )}{4 M^2_{G^\prime }}\\ C_6(m_c)&= 1.61\frac{\Delta _L^{s d}(G^\prime )\Delta _R^{q q}(G^\prime )}{4 M^2_{G^\prime }}. \end{aligned} \end{aligned}$$

Also the initial conditions for $\Delta S=2$ transition change:

118 $$\begin{aligned} C_1^\text {VLL}(M_{G^\prime })&= \left[ \frac{1}{3}\right] \frac{(\Delta _L^{sd}(G^\prime )^2}{2M_{G^\prime }^2},\nonumber \\ C_1^\text {VRR}(M_{G^\prime })&= \left[ \frac{1}{3}\right] \frac{(\Delta _R^{sd}(G^\prime ))^2}{2M_{G^\prime }^2}, \end{aligned}$$

119 $$\begin{aligned} C_1^\text {LR}(M_{G^\prime })&= \left[ -\frac{1}{6}\right] \frac{\Delta _L^{sd}(G^\prime )\Delta _R^{sd}(G^\prime )}{ M_{G^\prime }^2},\nonumber \\ C_2^\text {LR}(M_{G^\prime })&= \left[ -1\right] \frac{\Delta _L^{sd}(G^\prime )\Delta _R^{sd}(G^\prime )}{ M_{G^\prime }^2}. \end{aligned}$$

The NLO QCD corrections to tree-level coloured gauge boson exchanges at $\mu =M_{G^\prime }$ to $\Delta S=2$ are not known. They are expected to be small due to small QCD coupling at this high scale and serve mainly to remove certain renormalisation scheme and matching scale uncertainties. More important is the RG evolution from low energy scales to $\mu =M_{G^\prime }$ necessary to evaluate $\langle Q_1^\text {VLL}(M_{G^\prime })\rangle$ and $\langle Q_{1,2}^\text {LR}(M_{G^\prime })\rangle$. Here we include NLO QCD corrections using the technology in [62]. Again $Q_{1}^\text {VLL}$ remains the only operator in scenario B while $Q_{1,2}^\text {LR}$ contributing in scenario A help in solving the problem with $\Delta M_K$.

### $\mathrm{Re}A_0$ and $\mathrm{Im}A_0$

Proceeding as in the case of a colourless $Z^\prime$ we find

120 $$\begin{aligned} {\mathrm{Re}} A_0^\mathrm{NP}={{\mathrm{Re}} \Delta _L^{sd}}(G^\prime ) K^c_6(M_{G^\prime }) \left[ 0.7\times 10^{-8}\, \mathrm{GeV}\right] ,\end{aligned}$$

121 $$\begin{aligned} {\mathrm{Im}} A_0^\mathrm{NP}={{\mathrm{Im}}\Delta _L^{sd}}(G^\prime ) K^c_6(M_{G^\prime }) \left[ 0.7\times 10^{-8}\, \mathrm{GeV}\right] , \end{aligned}$$

where we have defined the $\mu$-independent factor

122 $$\begin{aligned} K_6(M_{G^\prime })&= -r^c_6(\mu ) \Delta _R^{qq}(G^\prime )\, \left[ \frac{3\, \mathrm{TeV}}{M_{G^\prime }}\right] ^2 \nonumber \\&\times \left[ \frac{114\, \mathrm{MeV}}{m_s(\mu ) + m_d(\mu )}\right] ^2 \,B_6^{(1/2)}\, \end{aligned}$$

with the renormalisation group factor $r^c_6(\mu )$ defined by

123 $$\begin{aligned} C_6(\mu ) = \left[ \frac{1}{2}\right] \frac{\Delta _L^{s d}(G^\prime )\Delta _R^{q q}(G^\prime )}{4 M^2_{G^\prime }} r^c_6(\mu ). \end{aligned}$$

Even if formulae (120) and (121) involve an explicit factor of $0.7$ instead of $1.4$ in the case of the colourless case, this decrease is overcompensated by the value of $r^c_6$, which for $\mu =1.3\, \mathrm{GeV}$ is found to be $r^c_6=3.23$, that is, by roughly a factor of 3 larger than $r_6$ in the colourless case.

Demanding now that $P\%$ of the experimental value of $\mathrm{Re}A_0$ in (1) comes from the $G^\prime$ contribution, we arrive at the condition:

124 $$\begin{aligned} {\mathrm{Re}\Delta _L^{sd}}(G^\prime ) K^c_6(M_{G^\prime }) = 7.8\, \left[ \frac{P\%}{20\,\%}\right] . \end{aligned}$$

Consequently the couplings $\mathrm{Re} \Delta _L^{sd}(G^\prime )$ and $\Delta _R^{q q}(G^\prime ))$ must have opposite signs and must satisfy

125 $$\begin{aligned} \mathrm{Re} \Delta _L^{sd}(G^\prime )\Delta _R^{q q}(G^\prime )\left[ \frac{3\, \mathrm{TeV}}{M_{Z^\prime }}\right] ^2B_6^{(1/2)}= -2.4\, \left[ \frac{P\%}{20\,\%}\right] .\nonumber \\ \end{aligned}$$

In view of the fact that $r^c_6$ is larger than $r_6$ by a factor of 2.9, ${\mathrm{Re}\Delta _L^{sd}}$ can be by a factor of $1.4$ smaller than in the colourless case in order to reproduce the data on $\mathrm{Re}A_0$.

We also find

126 $$\begin{aligned} {\mathrm{Im}} A_0^\mathrm{NP}=\frac{\mathrm{Im}\Delta _L^{sd}}{\mathrm{Re}\Delta _L^{sd}} \left[ \frac{P\%}{20\,\%}\right] \left[ 5.4\times 10^{-8}\, \mathrm{GeV}\right] . \end{aligned}$$

### $\Delta M_K$ constraint

Beginning with LHS scenario B we find that due to the modified initial conditions $\Delta S(K)$ is by the colour factor $1/3$ suppressed relative to the colourless case

127 $$\begin{aligned} \Delta S(K)=0.8\, \left[ \frac{\Delta _L^{sd}(G^\prime )}{\lambda _t}\right] ^2 \left[ \frac{3\, \mathrm{TeV}}{M_{G^\prime }}\right] ^2. \end{aligned}$$

Consequently allowing conservatively that the NP contribution is at most as large as the short distance SM contribution to $\Delta M_K$ we find the bound on a real $\Delta ^{sd}_L(G^\prime )$

128 $$\begin{aligned} |\Delta ^{sd}_L(G^\prime )|\le 0.007\, \left[ \frac{M_{G^\prime }}{3\, \mathrm{TeV}}\right] . \end{aligned}$$

This softer bound is still in conflict with (124) and we conclude that also in this case the LHS scenario does not provide a significant NP contribution to $\mathrm{Re} A_0$ when $\Delta M_K$ constraint is taken into account. On the other hand in this scenario there are no NP contributions to $K^+\rightarrow \pi ^+\nu \bar{\nu }$ and $K_{L}\rightarrow \pi ^0\nu \bar{\nu }$ because of the vanishing $G^\prime \nu \bar{\nu }$ coupling. This fact offers of course an important test of this scenario.

In scenario A for the couplings, assuming first for simplicity that the couplings $\Delta ^{sd}_{L,R}(G^\prime )$ are real, we find

129 $$\begin{aligned}&\Delta M_K(G^\prime ) = \frac{(\Delta _L^{sd}(G^\prime ))^2}{3M_{G^\prime }^2} \langle Q_1^\text {VLL}(M_{G^\prime })\rangle \nonumber \\&\quad \times \left[ 1+\left( \frac{\Delta _R^{sd}(G^\prime )}{\Delta _L^{sd}(G^\prime )}\right) ^2 +6\left( \frac{\Delta _R^{sd}(G^\prime )}{\Delta _L^{sd}(G^\prime )}\right) \frac{\langle Q^\text {LR}(M_{G^\prime })\rangle _c}{\langle Q_1^\text {VLL}(M_{G^\prime })\rangle }\right] ,\nonumber \\ \end{aligned}$$

with $\langle Q_1^\text {VLL}(M_{G^\prime })\rangle$ as before but

130 $$\begin{aligned}&\langle Q^\text {LR}(M_{G^\prime })\rangle _c\equiv -\frac{1}{6}\langle Q_1^\text {LR}(M_{G^\prime })\rangle \nonumber \\&\quad -\langle Q_2^\text {LR}(M_{G^\prime })\rangle \approx -143\, \langle Q_1^\text {VLL}(M_{G^\prime })\rangle . \end{aligned}$$

We indicate with the subscript ”c” that the initial conditions for the Wilson coefficients are modified relative to the case of a colourless $Z^\prime$. Hadronic matrix elements remain of course unchanged except that in view of the absence of NLO QCD corrections at the high matching scale no *hats* are present.

Denoting then the analogue of the suppression factor $\delta$ by $\delta _c$ we find that the required suppression of $\Delta M_K$ is given by

131 $$\begin{aligned} \delta _c&= 0.002 \left[ \frac{r^c_6(m_c)}{3.23}\right] \Delta _R^{qq}(G^\prime )\left[ \frac{3\, \mathrm{TeV}}{M_{G^\prime }}\right] B_6^{(1/2)}\left[ \frac{20\,\%}{P\%}\right] \,\nonumber \\ \end{aligned}$$

and in our toy model is given by

132 $$\begin{aligned} \delta _c&=\left[ 1+\left( \frac{\Delta _R^{sd}(G^\prime )}{\Delta _L^{sd}(G^\prime )}\right) ^2\right. \nonumber \\&\quad \left. +\, 6\left( \frac{\Delta _R^{sd}(G^\prime )}{\Delta _L^{sd} (G^\prime )}\right) \frac{\langle Q^\text {LR}(M_{G^\prime })\rangle _c}{\langle Q_1^\text {VLL}(M_{G^\prime })\rangle }\right] ^{1/2}. \end{aligned}$$

Consequently also in this case the problem with $\Delta M_K$ can be solved by suitably adjusting the coupling $\Delta _R^{sd}(G^\prime )$.

The expression for $\Delta _R^{sd}(G^\prime )$ in our toy model now reads

133 $$\begin{aligned}&\frac{\Delta _R^{sd}(G^\prime )}{\Delta _L^{sd}(G^\prime )}= -\frac{1}{6} R^c_Q(1+h (R^c_Q)^2),\nonumber \\&R^c_Q\equiv \frac{\langle Q_1^\text {VLL}((M_{G^\prime })\rangle }{\langle Q_1^\text {LR}((M_{G^\prime })\rangle _c}\approx -0.7\times 10^{-2}\nonumber \\ \end{aligned}$$

and consequently

134 $$\begin{aligned} \delta _c=\frac{1}{6} R^c_Q(1-36 h)^{1/2} +\mathcal {O}((R^c_Q)^2), \end{aligned}$$

which shows that by a proper choice of the parameter $h$ one can suppress the NP contributions to $\Delta M_K$ to the level that it agrees with experiment.

We find then

135 $$\begin{aligned}&\varepsilon _K(G^\prime )=-\frac{\kappa _\epsilon e^{i\varphi _\epsilon }}{\sqrt{2}(\Delta M_K)_\text {exp}}\frac{(\mathrm{Re}\Delta ^{sd}_L(G^\prime ))(\mathrm{Im}\Delta ^{sd}_L(G^\prime ))}{3\, M_{G^\prime }^2}\nonumber \\&\qquad \qquad \qquad \times \langle Q_1^\text {VLL}(M_{G^\prime })\rangle \delta _c^2\equiv \tilde{\varepsilon }_K(G^\prime )e^{i\varphi _\epsilon },\end{aligned}$$

136 $$\begin{aligned}&\Delta M_K(G^\prime )=\frac{(\mathrm{Re}\Delta ^{sd}_L(G^\prime ))^2}{3\, M_{G^\prime }^2} \langle Q_1^\text {VLL}(M_{G^\prime })\rangle \delta _c^2. \end{aligned}$$

Consequently we find the correlations

137 $$\begin{aligned}&\tilde{\varepsilon }_K(G^\prime )= -\frac{\kappa _\epsilon }{\sqrt{2} r_{\Delta M} }\left[ \frac{{\mathrm{Im}\Delta _L^{sd}(G^\prime )}}{{\mathrm{Re}\Delta _L^{sd}(G^\prime )}}\right] , \nonumber \\&r_{\Delta M}=\left[ \frac{(\Delta M_K)_\text {exp}}{\Delta M_K(G^\prime )}\right] ,\end{aligned}$$

138 $$\begin{aligned}&\left( \frac{\varepsilon '}{\varepsilon }\right) _{G^\prime }= \frac{3.5}{\kappa _\epsilon }\, \tilde{\varepsilon }_K(G^\prime ) \left[ \frac{P\%}{20\,\%}\right] r_{\Delta M}. \end{aligned}$$

We note that these correlations are exactly the same as in the colourless case and we can use the three step procedure used in the latter case. But there are the following differences, which will change the numerical analysis:

- The relation (125) differs from the one in (47) so that a smaller value of the product $|\mathrm{Re} \Delta _L^{sd}(G^\prime )\Delta _R^{q q}(G^\prime )|$ than of $|\mathrm{Re} \Delta _L^{sd}(Z^\prime )\Delta _R^{q q}(Z^\prime )|$ is required to obtain a given value of $P$.
- But the LHC constraints on $\Delta _R^{q q}(G^\prime )$, $\Delta _L^{sd}(G^\prime )$ and $M_{G^\prime }$ differ from the ones on $\Delta _R^{q q}(Z^\prime )$, $\Delta _L^{sd}(Z^\prime )$ and $M_{Z^\prime }$ and therefore in order to find whether $G^\prime$ or $Z^\prime$ contributes more to $\mathrm{Re}A_0$ these constraints have to be taken into account. See below.
- The NP contributions to $K^+\rightarrow \pi ^+\nu \bar{\nu }$ and $K_{L}\rightarrow \pi ^0\nu \bar{\nu }$ vanish.

### Numerical results

#### Scenario A

In the case of scenario A, we just follow the steps performed for $Z^\prime$ but, as the correlation between $\varepsilon '/\varepsilon$ and $\varepsilon _K$ is the same, we just indicate for which values of $B_6^{(1/2)}$ and $P$ this correlation is consistent with the data on $\varepsilon '/\varepsilon$ and $\varepsilon _K$ and the LHC constraints on the relevant couplings.

**Fig. 9:**
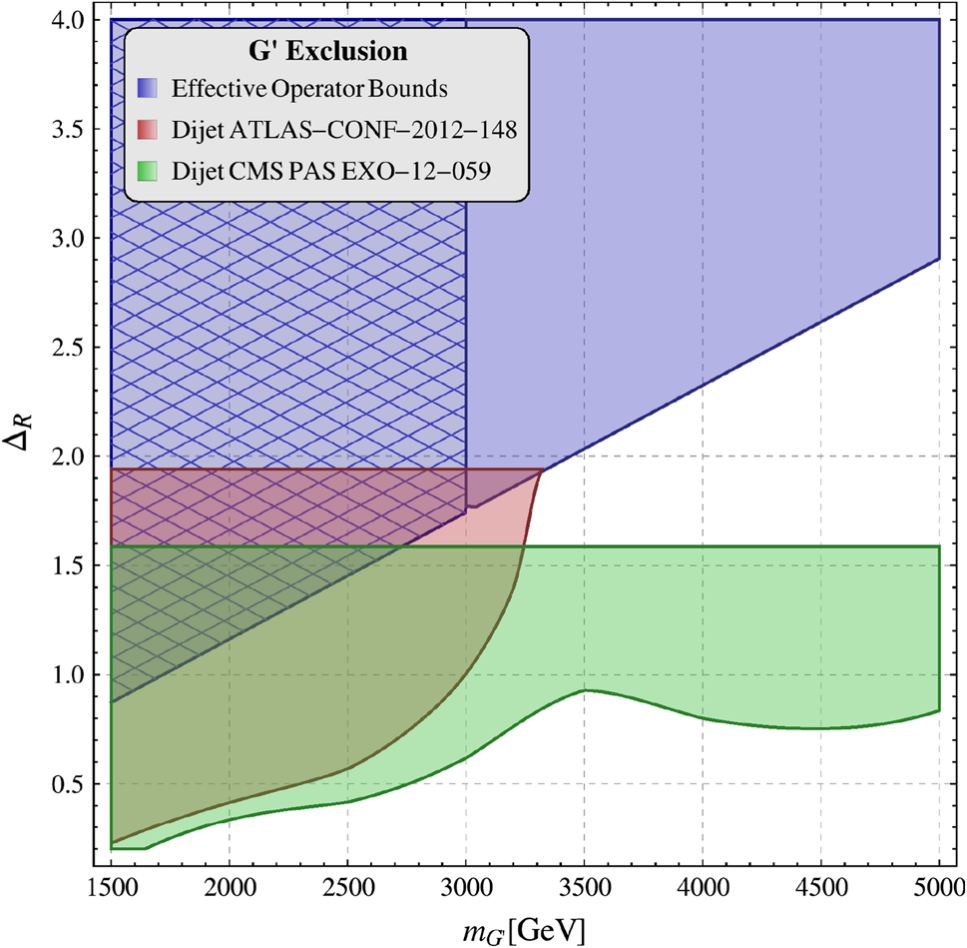
Exclusion limits for the $G'$ in the mass-coupling plane, from various searches at the LHC as found in [82]. The *blue region* is excluded by effective operator bounds provided by ATLAS [83] and CMS[84]. The *dashed surface* represents the region where the effective theory is not applicable, and the bounds here should be interpreted as a rough estimate. The *red* and *green contours* are excluded by dijet resonance searches by ATLAS [85] and CMS [86]. See for additional comments in the text

Concerning the LHC constraints a dedicated analysis of our toy $G^\prime$ model has been performed in [82] with the results given in Fig. 9. Additional comments made in connection with the bounds on $Z^\prime$ couplings in Fig. 3 also apply here. In particular the complete exclusion of the dashed surface would require a new ATLAS and CMS study in the context of our simple model.

These results can be summarised as follows:

- From dijets constraints the upper bounds can only be obtained for $|\Delta _R^{qq}(G^\prime )|\le 1.9$ and at this value only $M_{Z^\prime }\ge 3.3\, \mathrm{TeV}$ is allowed.
- The effective operator bounds can be summarised by
  139 $$\begin{aligned} |\Delta _R^{qq}(G^\prime )|\le 2.0\times \left[ \frac{M_{Z^\prime }}{3.5\, \mathrm{TeV}}\right] . \end{aligned}$$
  We note that the bound in this case is weaker than in the case of $Z^\prime$, which is partly the result of colour factors that suppress the NP contributions.
- We are not aware of any LHC bound on the $\Delta S=2$ operator in this case but we expect on the basis of the last finding that this bound is also weaker than the one on $\Delta _L^{sd}(Z^\prime )$ in (107). However, in the absence of any dedicated analysis we assume that the bound on $\Delta _L^{sd}(G^\prime )$ is as strong as the latter bound. A simple rescaling then gives
  140 $$\begin{aligned} |\Delta _L^{sd}(G^\prime )|\le 2.6 \left[ \frac{M_{Z^\prime }}{3.5\, \mathrm{TeV}}\right] . \end{aligned}$$

Even if a dedicated analysis of the latter bound would be necessary to put our analysis of LHC constraints on firm footing we conclude for the time being that $G^\prime$ copes much better with the missing piece in $\mathrm{Re}A_0$ than $Z^\prime$ and consequently can provide a significantly larger contribution than the SM QCD-penguin contribution. This is not only the result of the weaker LHC bound on $\Delta _R^{qq}$ but also of different renormalisation group effects, as seen in (125).

Putting all the factors together we conclude that $P$ as high as $30$–$35$ is still possible at present and this is sufficient to reproduce the $\Delta I=1/2$ rule within $5$–$10\,\%$. Indeed taking all these bounds into account and using (125) we arrive at the bound

141 $$\begin{aligned} P\le 32\left[ \frac{B_6^{(1/2)}}{1.0}\right] , \qquad (G^\prime )~. \end{aligned}$$

**Fig. 10:**
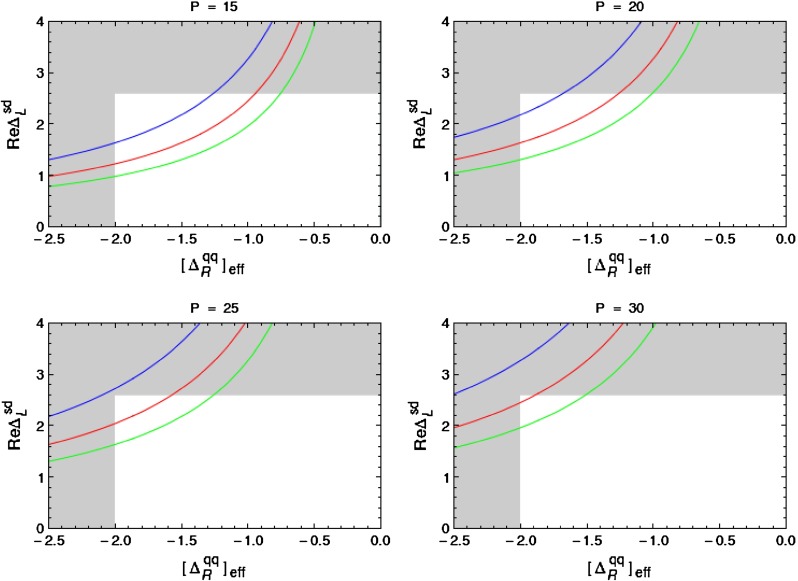
$\mathrm{Re} \Delta _L^{sd}(G^\prime )$ versus $|[\Delta _R^{q q}(G^\prime )]_\text {eff}|$ for $P = 15,~20,~25,~30$ and $B_6^{(1/2)}= 0.75$ (*blue*), $1.00$ (*red*) and $1.25$ (*green*). The *grey area* is basically excluded by the LHC. See additional comments in the text

In Fig. 10 we show the results for $G^\prime$ corresponding to Fig. 1. As now the values of $P$ can be larger we show the results for $P=15,~20,~25,~30$. With the definition

142 $$\begin{aligned}{}[\Delta _R^{q q}(G^\prime )]_\text {eff}=\Delta _R^{q q}(G^\prime )\left[ \frac{3.5\, \mathrm{TeV}}{M_{Z^\prime }}\right] ^2 \end{aligned}$$

the values in the grey area correspond to $|[\Delta _R^{q q}(G^\prime )]_\text {eff}|\ge 2.00$ and $\mathrm{Re}\Delta _L^{sd}(G^\prime )\ge 2.6$. Even if these values are already ruled out by the LHC it is evident that $G^\prime$ can provide significantly larger values of $P$ than $Z^\prime$. We do not show the plot corresponding to Fig. 4, as this correlation is also valid in the case of $G^\prime$, except that now also larger values of $P$, like 25–30, are allowed, which correspond to steeper lines than $P=20$ in Fig. 4.

#### Scenario B

In the case of scenario B in the absence of the $\Delta I=1/2$ constraint and NP contributions to $K^+\rightarrow \pi ^+\nu \bar{\nu }$ and $K_{L}\rightarrow \pi ^0\nu \bar{\nu }$ we can only illustrate how going from the $Z^\prime$ to the $G^\prime$ scenario modifies the allowed oases for $\Delta _L^{sd}$ when the $\varepsilon '/\varepsilon$, $\varepsilon _K$ and $\Delta M_K$ constraints are imposed. To this end we set^8^

143 $$\begin{aligned} \Delta _R^{qq}(G^\prime )=\Delta _R^{qq}(Z^\prime )= 0.5, \qquad M_{G^\prime }=M_{Z^\prime }=3.0\, \mathrm{TeV}\nonumber \\ \end{aligned}$$

and use in the $G^\prime$ case the formula (58) with ${\mathrm{Im}} A_0^\mathrm{NP}$ given in (121). For the corresponding contributions to $\varepsilon _K$ and $\Delta M_K$ we use the shift in the function $S$ given this time in (127).

In order to understand better the results below it should be noted that for the same values of the couplings $\Delta _R^{qq}$ and $\Delta _L^{sd}$ the contribution of $G^\prime$ to $\varepsilon '/\varepsilon$ is by a factor of $1.4$ larger than the $Z^\prime$ contribution. In the case of $\Delta M_K$ and $\varepsilon _K$ it is opposite: $G^\prime$ contribution is by a factor of $3$ smaller than in the $Z^\prime$ case.

**Fig. 11:**
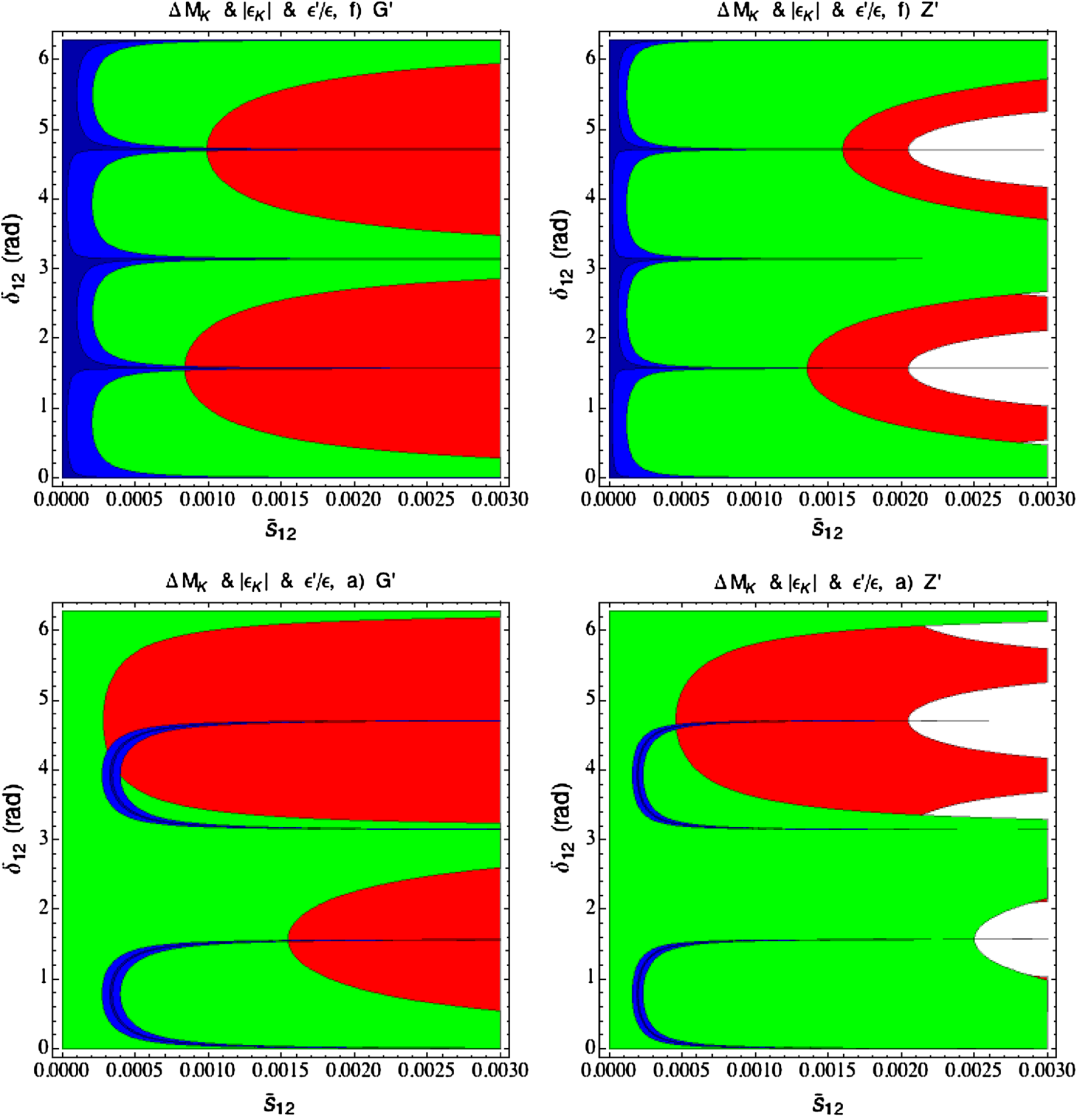
Ranges for $\Delta M_K$ (*red region*) and $\varepsilon _K$ (*blue region*) satisfying the bounds in Eq. (94) (*lighter blue*) and within its $3\sigma$ experimental range (*darker blue*) and $\varepsilon '/\varepsilon$ (*green region*) within its $2\sigma$ range $[11.3,\,21.7]\times 10^{-4}$ for $B_6^{(1/2)} =1$ and $\Delta _R^{qq} = 0.5$ (*green*) for CKM scenario $(f)$ (*top*) and $(a)$ (*down*) and $G'$ (*left*) and $Z'$ (*right*)

In Fig. 11 we compare the oases obtained in this manner for $G^\prime$ with those obtained for $Z^\prime$ for $B_6^{(1/2)}=1.00$ and the scenarios $(f)$ and $(a)$ for $(|V_{cb}|,|V_{ub}|)$. To this end we have used the $2\sigma$ constraint for $\varepsilon '/\varepsilon$ with (143) shown in *green.* For $\varepsilon _K$ we impose either softer constraint (lighter blue region) in (94) or a tighter $3\sigma$ experimental range (darker blue).

We observe the following features:

- In all plots the $3\sigma$ constraint from $\varepsilon _K$ (dark blue) determines the allowed oasis simply because the present experimental error on $\varepsilon '/\varepsilon$ is unfortunately significant.
- The bound on $\Delta _L^{sd}$ from $\varepsilon _K$ is stronger in the case of $Z^\prime$. On the other hand the corresponding bound from $\varepsilon '/\varepsilon$ is stronger in the case of $G^\prime$. Both properties follow from the different numerical factors in $\varepsilon '/\varepsilon$ and $\varepsilon _K$ summarised above.
- In scenario $(f)$, the coupling $\Delta _L^{sd}$ can vanish as SM value for $\varepsilon _K$ is very close to the data. This is not the case in scenario $(a)$, in which the SM value is well below the data and NP is required to enhance $\varepsilon _K$.
- In spite of the weak constraint from $\varepsilon '/\varepsilon$, also $\varepsilon '/\varepsilon$ in scenario $(a)$ has to be enhanced. This helps us to distinguish between two oases that follow from $\varepsilon _K$ favouring the one with smaller $\delta _{12}$, in which $\varepsilon '/\varepsilon$ is enhanced over its SM value. But the large experimental error on $\varepsilon '/\varepsilon$ does not allow one to exclude the second oasis in which $\varepsilon '/\varepsilon$ is suppressed unless $1\sigma$ constraint on $\varepsilon '/\varepsilon$ is used.

In presenting these results we have set $B_6^{(1/2)}=1.0$. Choosing different values would change the role of $\varepsilon '/\varepsilon$ but we do not show these results as it is straightforward to deduce the pattern of NP effects for these different values of $B_6^{(1/2)}$. Similar comment applies to other CKM scenarios.

## The case of $Z$ boson with FCNCs

### Preliminaries

We will next discuss the scenario of $Z$ with FCNC couplings in order to demonstrate that the missing piece in $\mathrm{Re}A_0$ cannot come from this corner, as this would imply total destruction of the SM agreement with the data on $\mathrm{Re}A_2$. Still interesting results for $\varepsilon '/\varepsilon$ and its correlation with the branching ratios for $K^+\rightarrow \pi ^+\nu \bar{\nu }$ and $K_{L}\rightarrow \pi ^0\nu \bar{\nu }$ can be found. They are more specific than in the $Z^\prime$ case due to the knowledge of all flavour diagonal couplings of $Z$ and of its mass.

Indeed the only freedom in the kaon system in this NP scenario are the complex couplings $\Delta ^{sd}_{L,R}(Z)$. Its detailed phenomenology including $\Delta S=2$ transitions and rare kaon decays has been presented by us in [26]. This section generalises that analysis to $K\rightarrow \pi \pi$ decays; in particular, the $\varepsilon '/\varepsilon$ constraint will eliminate some portions of the large enhancements found by us for the branching ratios of rare $K$ decays.

In order to understand better our results for $K^+\rightarrow \pi ^+\nu \bar{\nu }$ and $K_{L}\rightarrow \pi ^0\nu \bar{\nu }$ in the presence of simultaneous constraints from $\varepsilon '/\varepsilon$ and $K_L\rightarrow \mu ^+\mu ^-$ in addition to the $\Delta S=2$ constraints let us recall that $\varepsilon '/\varepsilon$ puts constraints only on imaginary parts of the NP contributions, while $K_L\rightarrow \mu ^+\mu ^-$ only puts constraints on the real ones. As demonstrated already in [26] the impact of the latter constraint on $K^+\rightarrow \pi ^+\nu \bar{\nu }$ and $K_{L}\rightarrow \pi ^0\nu \bar{\nu }$ depends strongly on the scenario for the $Z$ flavour-violating couplings: LHS, RHS, LRS, ALRS and to a lesser extent on the CKM scenarios considered. Moreover, it has a different impact on $K^+\rightarrow \pi ^+\nu \bar{\nu }$ and $K_{L}\rightarrow \pi ^0\nu \bar{\nu }$, as the latter decay is only sensitive to the imaginary parts in the NP contributions. Let us summarise briefly these findings adding right away brief comments on $\varepsilon '/\varepsilon$:

- In the LHS scenario the branching ratio for $K_L\rightarrow \mu ^+\mu ^-$ is strongly enhanced relatively to its SM value and this limits possible enhancement of $\mathcal {B}(K^+\rightarrow \pi ^+\nu \bar{\nu })$. But $K^+\rightarrow \pi ^+\nu \bar{\nu }$ receives also an NP contribution from imaginary parts so that its branching ratio is strongly correlated with the one for $K_{L}\rightarrow \pi ^0\nu \bar{\nu }$ on the branch on which both branchings can be significantly modified. As we will see below the imposition of the $\varepsilon '/\varepsilon$ constraint will eliminate some parts of these modifications but this will depend on $B_6^{(1/2)}$ and on the scenarios for the CKM parameters considered.
- In the RHS scenario the $K_L\rightarrow \mu ^+\mu ^-$ constraint has a different impact on $K^+\rightarrow \pi ^+\nu \bar{\nu }$. Indeed, as $K_L\rightarrow \mu ^+\mu ^-$ is sensitive to axial-vector couplings there is a sign flip in the NP contributions to the relevant decay amplitude, while there is no sign flip in the case of $K^+\rightarrow \pi ^+\nu \bar{\nu }$. Consequently the impact of $K_L\rightarrow \mu ^+\mu ^-$ on $K^+\rightarrow \pi ^+\nu \bar{\nu }$ is now much weaker on the branch where there is no NP contribution to $K_{L}\rightarrow \pi ^0\nu \bar{\nu }$, but on the branch where $K^+\rightarrow \pi ^+\nu \bar{\nu }$ and $K_{L}\rightarrow \pi ^0\nu \bar{\nu }$ are strongly correlated we will find the impact of the $\varepsilon '/\varepsilon$ constraint.
- In the LRS scenario there are no NP contributions to $K_L\rightarrow \mu ^+\mu ^-$ so that, as already found in Fig. 30 of [26], very large NP effects in $K^+\rightarrow \pi ^+\nu \bar{\nu }$ and $K_{L}\rightarrow \pi ^0\nu \bar{\nu }$ without $\varepsilon '/\varepsilon$ constraint can be found. $\varepsilon '/\varepsilon$ will again constrain both decays on the branch where these decays are strongly correlated but leave the other branch unaffected.
- In the ALRS scenario the NP contributions to $K^+\rightarrow \pi ^+\nu \bar{\nu }$ and $K_{L}\rightarrow \pi ^0\nu \bar{\nu }$ vanish. $\varepsilon '/\varepsilon$ receives NP contributions but they are unaffected by the ones in $K_L\rightarrow \mu ^+\mu ^-$. In this scenario then $\varepsilon '/\varepsilon$ is not correlated with rare $K$ decays and the only question we can ask is how the NP physics contributions to $\varepsilon '/\varepsilon$ are correlated with the ones present in $\varepsilon _K$.

### $\mathrm{Re}A_0$ and $\mathrm{Re}A_2$

It is straightforward to calculate the values of the Wilson coefficients entering the NP part of the $K\rightarrow \pi \pi$ Hamiltonian. The non-vanishing Wilson coefficients at $\mu =M_{Z}$ are then given at the LO as follows:

144 $$\begin{aligned} \begin{aligned} C_3(M_{Z})&= -\left[ \frac{g}{6 c_W}\right] \frac{\Delta _L^{s d}(Z)}{4 M^2_Z},\\ C_5^\prime (M_Z)&= -\left[ \frac{g}{6 c_W}\right] \frac{\Delta _R^{s d}(Z)}{4 M^2_Z}, \end{aligned} \end{aligned}$$

145 $$\begin{aligned} \begin{aligned} C_7(M_Z)&= -\left[ \frac{4 g s_W^2}{6 c_W}\right] \frac{\Delta _L^{s d}(Z)}{4 M^2_Z},\\ C_9^\prime (M_Z)&= -\left[ \frac{4 g s_W^2}{6 c_W}\right] \frac{\Delta _R^{s d}(Z)}{4 M^2_Z}\, \end{aligned} \end{aligned}$$

146 $$\begin{aligned} \begin{aligned} C_9(M_Z)&= \left[ \frac{4 g c_W^2}{6 c_W}\right] \frac{\Delta _L^{s d}(Z)}{4M^2_Z},\\ C_7^\prime (M_Z)&= \left[ \frac{4 g c_W^2}{6 c_W}\right] \frac{\Delta _R^{s d}(Z)}{4 M^2_Z}. \end{aligned} \end{aligned}$$

We have used the well-known flavour conserving couplings of $Z$ to the quarks, which are collected in the same notation in the appendix in [33]. The $SU(2)_L$ gauge coupling constant is $g(M_Z)=0.652$. We note that the values of the coefficients in front of $\Delta _{L,R}$ are in the case of $C_9$ and $C_7^\prime$ by a factor of 3 larger than for the remaining coefficients.

We will first discuss the LHS scenario so that $\Delta _R^{s d}(Z)=0$. Similar to $Z^\prime$ scenarios only left–right operators are relevant at low energy scales but this time it is the electroweak penguin operator $Q_8$ that dominates the scene. Concentrating then on the operators $Q_7$ and $Q_8$, the relevant one-loop anomalous dimension matrix in the $(Q_7,Q_8)$ basis is very similar to the one in (20),

147 $$\begin{aligned} \hat{\gamma }^{(0)}_s = \left( \begin{array}{cc} 2 &{}\quad -6 \\ 0 &{}\quad -16 \end{array}\right) . \end{aligned}$$

Performing the renormalisation group evolution from $M_Z$ to $m_c=1.3\, \mathrm{GeV}$ we find

148 $$\begin{aligned} C_7(m_c)= 0.87\, C_7(M_Z)\qquad C_8(m_c)= 0.76\, C_7(M_Z).\nonumber \\ \end{aligned}$$

Due to the large element $(1,2)$ in the matrix (147) and the large anomalous dimension of the $Q_8$ operator represented by the $(2,2)$ element in (147), the two coefficients are comparable in size. But the matrix elements $\langle Q_7\rangle _{0,2}$ are colour suppressed, which is not the case of $\langle Q_8\rangle _{0,2}$, and within a good approximation we can neglect the contributions of $Q_7$. In summary, it is sufficient to keep only the $Q_8$ contributions in the decay amplitudes in this scenario for flavour-violating $Z$ couplings.

We find then

149 $$\begin{aligned} {\mathrm{Re}} A_0^\mathrm{NP}&= {\mathrm{Re}} C_8(m_c)\langle Q_8(m_c)\rangle _0,\nonumber \\ {\mathrm{Re}} A_2^\mathrm{NP}&= {\mathrm{Re}} C_8(m_c)\langle Q_8(m_c)\rangle _2. \end{aligned}$$

Now the relevant hadronic matrix elements of $Q_8$ operator are given as follows:

150 $$\begin{aligned}&\frac{\langle Q_8(m_c)\rangle _2}{\langle Q_6(m_c)\rangle _0}\approx -\frac{R_8}{R_6}\frac{F_\pi }{2\sqrt{2}(F_K-F_\pi )}\nonumber \\&\qquad \qquad \qquad =-1.74 \,\frac{B_8^{(3/2)}}{B_6^{(1/2)}},\end{aligned}$$

151 $$\begin{aligned}&\frac{{\mathrm{Re}} A_2^\mathrm{NP}}{{\mathrm{Re}} A_0^\mathrm{NP}}=\frac{\langle Q_8(m_c)\rangle _2}{\langle Q_8(m_c)\rangle _0}\approx \frac{F_\pi }{\sqrt{2}F_K}\frac{B_8^{(3/2)}}{B_8^{(1/2)}}\nonumber \\&\,\,\qquad \qquad =0.59 \frac{B_8^{(3/2)}}{B_8^{(1/2)}}, \end{aligned}$$

with $B_8^{(3/2)}=B_8^{(1/2)}=1$ in the large $N$ limit but otherwise expected to be $\mathcal {O}(1)$ as confirmed in the case of $B_8^{(3/2)}$ by lattice QCD [21].

It is evident from (151) that the explanation of the missing piece in ${\mathrm{Re}} A_0$ with $Z$ exchange would totally destroy the agreement of the SM with the data on ${\mathrm{Re}} A_2$. Rather we should investigate the constraint on ${\mathrm{Re}} \Delta _L^{sd}(Z)$, which would allow us to keep this agreement in the presence of $Z$ with FCNC couplings.

Demanding then that at most $P\%$ of the experimental value of $\mathrm{Re}A_2$ in (1) comes from the $Z$ contribution, we arrive at the condition

152 $$\begin{aligned} |\mathrm{Re} \Delta _L^{sd}(Z) K_8(Z)| \le 6.2\times 10^{-4}\, \left[ \frac{P\%}{10\,\%}\right] , \end{aligned}$$

where

153 $$\begin{aligned} K_8(M_{Z})=- r_8(\mu ) \, \left[ \frac{114\, \mathrm{MeV}}{m_s(\mu ) + m_d(\mu )}\right] ^2 \, \left[ \frac{B_8^{(3/2)}}{0.65}\right] .\nonumber \\ \end{aligned}$$

The renormalisation group factor $r_8(m_c)=0.76$ is defined by

154 $$\begin{aligned} C_8(\mu )=r_8(\mu ) C_7(M_Z), \end{aligned}$$

with $C_7(M_Z)$ given in (145).

Consequently we arrive at the condition

155 $$\begin{aligned} |\mathrm{Re} \Delta _L^{sd}(Z)|\frac{B_8^{(3/2)}}{0.65}\le 8.2\times 10^{-4}\left[ \frac{P\%}{10\,\%}\right] . \end{aligned}$$

In fact this bound is weaker than the one following from $\Delta M_K$. Replacing $M_{Z^\prime }$ by $M_Z$, the bound in (70) is now replaced by

156 $$\begin{aligned} |\Delta ^{sd}_L(Z)|\le 1.2 \times 10^{-4}. \end{aligned}$$

Consequently imposing the $\Delta M_K$ bound in the numerical analysis below we are confident that no relevant NP contribution to ${\mathrm{Re}} A_2$ is present.

### $\varepsilon '/\varepsilon$, $K^+\rightarrow \pi ^+\nu \bar{\nu }$ and $K_{L}\rightarrow \pi ^0\nu \bar{\nu }$

We could as in the $Z^\prime$ case calculate separately the NP contribution to $\varepsilon '/\varepsilon$. However, in the present case the initial conditions for Wilson coefficients are at the electroweak scale as in the SM and it is easier to modify the functions $X$, $Y$ and $Z$ entering the analytic formula (53). We find then the shifts

157 $$\begin{aligned} \Delta X=\Delta Y =\Delta Z= c_W\frac{8\pi ^2}{g^3}\frac{\mathrm{Im}\Delta _L^{sd}(Z)}{\mathrm{Im}\lambda _t}. \end{aligned}$$

In doing this we include in fact all operators whose Wilson coefficients are affected by NP but effectively only the operator $Q_8$ is really relevant. The final formula for $\varepsilon '/\varepsilon$ in LHS scenario is then given by

158 $$\begin{aligned} \left( \frac{\varepsilon '}{\varepsilon }\right) _\text {LHS}= \left( \frac{\varepsilon '}{\varepsilon }\right) _\text {SM}+ \left( \frac{\varepsilon '}{\varepsilon }\right) ^L_{Z} \end{aligned}$$

where the second term stands for the modification related to the shifts in (157).

It should be emphasised that the shifts in (157) should only be used in the formula (53) so that $\mathrm{Im}\lambda _t$ cancels the one present in the SM contribution. $\Delta X$ can also be used in the case of $K_{L}\rightarrow \pi ^0\nu \bar{\nu }$. However, in the case of $K^+\rightarrow \pi ^+\nu \bar{\nu }$, where also real parts matter one should use the general formula

159 $$\begin{aligned} \Delta X= c_W\frac{8\pi ^2}{g^3}\frac{\Delta _L^{sd}(Z)}{\lambda _t}\, \end{aligned}$$

or equivalently simply use the formulae for $K^+\rightarrow \pi ^+\nu \bar{\nu }$ and $K_{L}\rightarrow \pi ^0\nu \bar{\nu }$ in the LHS scenario in [26].

### Numerical analysis in the LHS scenario

In [26] we have performed a detailed analysis of $K^+\rightarrow \pi ^+\nu \bar{\nu }$ and $K_{L}\rightarrow \pi ^0\nu \bar{\nu }$ decays in this NP scenario, imposing the constraints listed above and from $K_L\rightarrow \mu ^+\mu ^-$ decay, that is only relevant for $K^+\rightarrow \pi ^+\nu \bar{\nu }$. The present analysis generalises that analysis in two respects:

- We consider several scenarios $(a)$–$(f)$ for CKM parameters.
- We analyse the correlation between $\varepsilon '/\varepsilon$ and the branching ratios for $K^+\rightarrow \pi ^+\nu \bar{\nu }$ and $K_{L}\rightarrow \pi ^0\nu \bar{\nu }$.

**Fig. 12:**
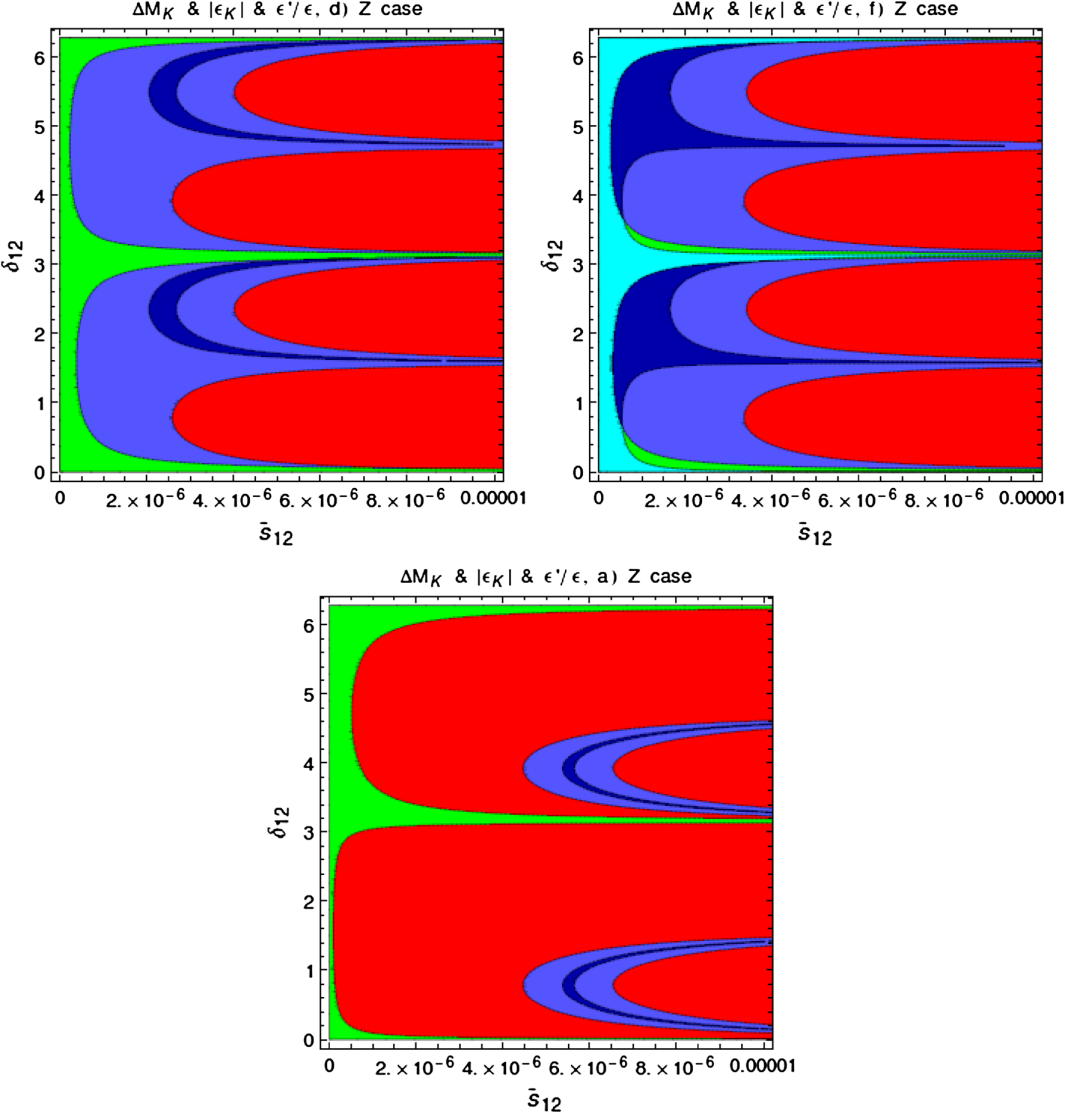
Ranges for $\Delta M_K$ (*red region*) and $\varepsilon _K$ (*blue region*) satisfying the bounds in Eq. (94) (*lighter blue*) and within its $3\sigma$ experimental range (*darker blue*) and $\varepsilon '/\varepsilon$ (*green region*) within its $2\sigma$ range $[11.3,\,21.7]\times 10^{-4}$ for $B_6^{(1/2)} =1$ for CKM scenario $(d)$ (*top left*), $(f)$ (*top right*) and $(a)$ (*down*). The *cyan* region in case $(f)$ corresponds to the overlap between the *green* and *dark blue* region

**Fig. 13:**
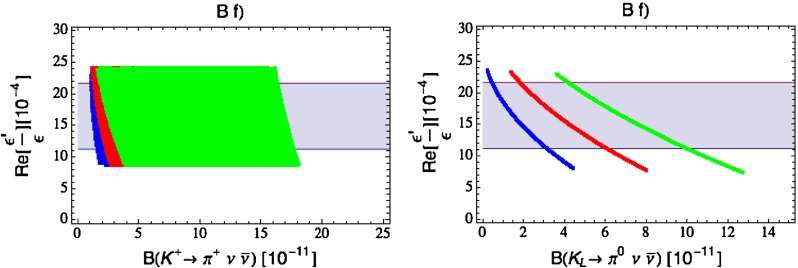
$\varepsilon '/\varepsilon$ versus $\mathcal {B}(K^+\rightarrow \pi ^+\nu \bar{\nu })$ (*left*) and $\varepsilon '/\varepsilon$ versus $\mathcal {B}(K_{L}\rightarrow \pi ^0\nu \bar{\nu })$ (*right*) in LHS for scenario $(f)$ including the constraints from $\Delta M_K$, $\varepsilon _K$ from Eq. (94), $\varepsilon '/\varepsilon$ within its $3\sigma$ experimental range for $B_6^{(1/2)} = 0.75$ (*blue*) $B_6^{(1/2)} = 1$ (*red*) and $B_6^{(1/2)} = 1.25$ (*green*) and $\mathcal {B}(K_L\rightarrow \mu ^+\mu ^-)\le 2.5\times 10^{-9}$. *Grey range* experimental $2\sigma$ range for $\varepsilon '/\varepsilon$

**Fig. 14:**
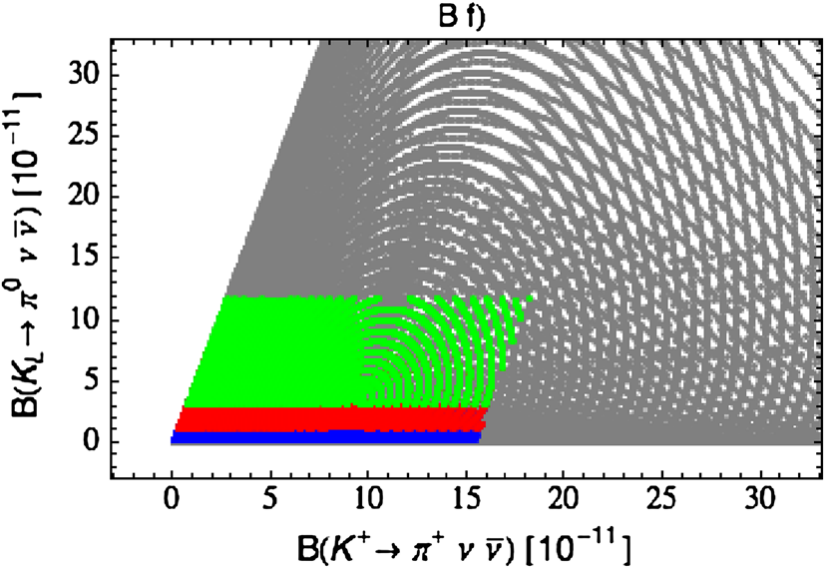
$\mathcal {B}(K_{L}\rightarrow \pi ^0\nu \bar{\nu })$ versus $\mathcal {B}(K^+\rightarrow \pi ^+\nu \bar{\nu })$ in LHS for scenario $(f)$ including the constraints from $\Delta M_K$, $\varepsilon _K$ from Eq. (94) (*grey region*) and $\varepsilon '/\varepsilon$ within its $3\sigma$ experimental range for $B_6^{(1/2)} = 0.75$ (*blue*) $B_6^{(1/2)} = 1$ (*red*) and $B_6^{(1/2)} = 1.25$ (*green*) and $\mathcal {B}(K_L\rightarrow \mu ^+\mu ^-)\le 2.5\times 10^{-9}$

It is straightforward to convince oneself that unless $\mathrm{Im}\Delta _L^{sd}(Z)= \mathcal {O}(10^{-8})$ the shifts in (157) imply modifications of $\varepsilon '/\varepsilon$ that are not allowed by the data. In turn, the NP contributions to $\varepsilon _K$ are negligible and the model can only agree with data on $\varepsilon _K$ for which also the SM agrees with them. Similar to scenario A in $Z^\prime$ case only scenarios $(d)$ and $(f)$ survive the $\varepsilon '/\varepsilon$ constraint. This can be seen in the oases plots in Fig. 12. In scenario $(d)$ shown there, and even more in scenario $(f)$, there is an overlap region of the blue ($\varepsilon _K$) and green ($\varepsilon '/\varepsilon$) range whereas in $(a)$ and also in the other CKM scenarios there is none. However, while in scenario $(d)$ there is a clear overlap between the $2\sigma$ range of $\varepsilon '/\varepsilon$ and the larger range of $\varepsilon _K$ in Eq. (94) (lighter blue), when using the smaller experimental $3\sigma$ range of $\varepsilon _K$ (darker blue) the overlap is tiny. In contrast in scenario $(f)$ the cyan region corresponds to the overlap of the darker blue and green region. Therefore in Fig. 13 we show the correlation of $\varepsilon '/\varepsilon$ and branching ratios for $K^+\rightarrow \pi ^+\nu \bar{\nu }$ and $K_{L}\rightarrow \pi ^0\nu \bar{\nu }$ and in Fig. 14 for the correlation between $K^+\rightarrow \pi ^+\nu \bar{\nu }$ and $K_{L}\rightarrow \pi ^0\nu \bar{\nu }$ only for the $(f)$ scenario. However, we checked that in scenario $(d)$ similar results are obtained and this is also the case of RHS, LRS and ALRS scenarios considered below. Therefore in the remainder of this section only results for scenario $(f)$ will be shown.

Comparing these results with those in the plots in Figs. 6, 7 and 8 for $Z^\prime$ we observe that they are more specific as the diagonal couplings of $Z$ and its mass are known and only selected CKM scenarios are allowed. While significant deviations from SM values for $\varepsilon '/\varepsilon$, $\mathcal {B}(K_{L}\rightarrow \pi ^0\nu \bar{\nu })$ and $\mathcal {B}(K^+\rightarrow \pi ^+\nu \bar{\nu })$ are in principle possible, the bounds from $\varepsilon '/\varepsilon$ and $K_L\rightarrow \mu ^+\mu ^-$ that are imposed in these plots do not allow very large enhancements of both branching ratios to occur. In particular the bound from $\varepsilon '/\varepsilon$ does not allow for the large enhancements of $\mathcal {B}(K_{L}\rightarrow \pi ^0\nu \bar{\nu })$ that we found in [26]. This analysis shows again how important the $\varepsilon '/\varepsilon$ constraint is. The correlation between $\mathcal {B}(K_{L}\rightarrow \pi ^0\nu \bar{\nu })$ versus $\mathcal {B}(K^+\rightarrow \pi ^+\nu \bar{\nu })$ shown in Fig. 14 demonstrates in a spectacular manner the action of the $\varepsilon '/\varepsilon$ and $K_L\rightarrow \mu ^+\mu ^-$ constraints. Without them the full grey region would still be allowed by $\Delta M_K$ and $\varepsilon _K$ constraints.

The correlation in the right panel of Fig. 13 is similar to the one encountered in other NP scenarios in which NP in $\varepsilon '/\varepsilon$ is dominated by electroweak penguins and the increase of $\mathcal {B}(K_{L}\rightarrow \pi ^0\nu \bar{\nu })$ implies automatically the suppression of $\varepsilon '/\varepsilon$. Therefore only for $B_6^{(1/2)}> 1.0$, where $\varepsilon '/\varepsilon$ within the SM is above the data, large enhancements of $\mathcal {B}(K_{L}\rightarrow \pi ^0\nu \bar{\nu })$ are possible. For the same sign of the neutrino coupling in scenario B for $Z^\prime$ and $\Delta _R^{qq}(Z^\prime )>0$ the correlation between $\varepsilon '/\varepsilon$ and $\mathcal {B}(K_{L}\rightarrow \pi ^0\nu \bar{\nu })$ is different, as seen in Fig. 7, because there the QCD-penguin operator $Q_6$ instead of $Q_8$ encountered here is at work.

### The RHS scenario

We discuss next the RHS scenario as here the pattern of the NP effects differs from the LHS case. In this scenario NP in $K\rightarrow \pi \pi$ is dominated by left–right primed operators. This time both $Q_6^\prime$ and $Q_8^\prime$ have to be considered although at the end only the latter operator will be important. Within a very good approximation we have

160 $$\begin{aligned} A_0^\mathrm{NP}&= C^\prime _6(m_c)\langle Q^\prime _6(m_c)\rangle _0+C^\prime _8(m_c)\langle Q^\prime _8(m_c)\rangle _0,\end{aligned}$$

161 $$\begin{aligned} A_2^\mathrm{NP}&= C^\prime _8(m_c)\langle Q^\prime _8(m_c)\rangle _2 \end{aligned}$$

where

162 $$\begin{aligned} C^\prime _6(m_c)=r_6^\prime (m_c) C^\prime _5(M_Z), \quad C^\prime _8(m_c)=r_8^\prime (m_c) C^\prime _7(M_Z)\nonumber \\ \end{aligned}$$

with

163 $$\begin{aligned} r_6^\prime (m_c)\approx r_8^\prime (m_c)=r_8(m_c)=0.76. \end{aligned}$$

Moreover, one has

164 $$\begin{aligned}&\langle Q^\prime _6(m_c)\rangle _0=-\langle Q_6(m_c)\rangle _0,\nonumber \\&\langle Q^\prime _8(m_c)\rangle _{0,2}=-\langle Q_8(m_c)\rangle _{0,2}. \end{aligned}$$

Proceeding as in the LHS scenario we again find that one cannot explain the missing piece in ${\mathrm{Re}} A_0$ with $Z$ exchange without totally destroying the agreement of the SM with the data on ${\mathrm{Re}} A_2$. Due to the different initial conditions the upper bound in (155) is replaced by a stronger bound,

165 $$\begin{aligned} |\mathrm{Re} \Delta _R^{sd}(Z)|\left[ \frac{B_8^{(3/2)}}{0.65}\right] \le 2.5\times 10^{-4}\left[ \frac{P\%}{10\,\%}\right] . \end{aligned}$$

But in the RHS scenario the bound on $|\mathrm{Re} \Delta _R^{sd}(Z)|$ from $\Delta M_K$ is the same as the one for $|\mathrm{Re} \Delta _L^{sd}(Z)|$ in the LHS scenario and consequently no problem with ${\mathrm{Re}} A_2$ arises after the bound from $\Delta M_K$ has been taken into account.

Taking first into account both the $Q^\prime _6$ and $Q^\prime _8$ contributions to $\varepsilon '/\varepsilon$, we have

166 $$\begin{aligned} \left( \frac{\varepsilon '}{\varepsilon }\right) _{Z}= -\frac{\omega _+}{|\varepsilon _K|\sqrt{2}} \left[ \frac{{\mathrm{Im}} A_0^\mathrm{NP}}{{\mathrm{Re}}A_0}(1-\Omega _\mathrm{eff})-\frac{{\mathrm{Im}} A_2^\mathrm{NP}}{{\mathrm{Re}}A_2}\right] ,\nonumber \\ \end{aligned}$$

where ${\mathrm{Re}}A_0$ and ${\mathrm{Re}}A_2$ are to be taken from (1).

While both $Q^\prime _6$ and $Q^\prime _8$ contribute, the latter operator wins easily this competition because it is not only enhanced through the $\Delta I=1/2$ rule relative to $Q_6^\prime$ contribution to $\varepsilon '/\varepsilon$ but also because its Wilson coefficient is larger than the one of $Q^\prime _6$. This is in contrast to the competition between $Q_6$ and $Q_8$ in the SM, where the much larger Wilson coefficient of $Q_6$ overcompensates the $\Delta I=1/2$ rule effect in question. Thus keeping only the $Q^\prime _8$ operator we find within an excellent approximation

167 $$\begin{aligned}&\left( \frac{\varepsilon '}{\varepsilon }\right) ^R_{Z}= \frac{\omega _+}{|\varepsilon _K|\sqrt{2}}\frac{{\mathrm{Im}} A_2^\mathrm{NP}}{{\mathrm{Re}}A_2}= -5.3\nonumber \\&\quad \times 10^3 \, \left[ \frac{114\, \mathrm{MeV}}{m_s(\mu ) + m_d(\mu )}\right] ^2 \, \left[ \frac{B_8^{(3/2)}}{0.65}\right] \,\mathrm{Im} \Delta _R^{sd}(Z) \end{aligned}$$

implying that $\mathrm{Im} \Delta _R^{sd}(Z)$ must be $\mathcal {O}(10^{-8})$ in order for $\varepsilon '/\varepsilon$ to agree with experiment. Then, similar to the LHS case just discussed, the NP contributions to $\varepsilon _K$ are negligible and consequently only scenarios $(d)$ and $(f)$ for the CKM parameters survive the test.

The final formula for $\varepsilon '/\varepsilon$ in the RHS scenario is now given by

168 $$\begin{aligned} \left( \frac{\varepsilon '}{\varepsilon }\right) _\text {RHS}= \left( \frac{\varepsilon '}{\varepsilon }\right) _\text {SM}+ \left( \frac{\varepsilon '}{\varepsilon }\right) ^R_{Z}, \end{aligned}$$

where the second term is given in (167).

As far as $K^+\rightarrow \pi ^+\nu \bar{\nu }$ and $K_{L}\rightarrow \pi ^0\nu \bar{\nu }$ are concerned we can use the formulae in [26]. Equivalently in the case of the RHS scenario one can just make a shift in the function $X(K)$:

169 $$\begin{aligned} \Delta X(K)&= \left[ \frac{\Delta _L^{\nu \bar{\nu }}(Z)}{g^2_\mathrm{SM}M_{Z}^2}\right] \left[ \frac{\Delta _R^{sd}(Z)}{\lambda _t}\right] ,\nonumber \\ \Delta _L^{\nu \bar{\nu }}(Z)&= \frac{g}{2c_W}. \end{aligned}$$

**Fig. 15:**
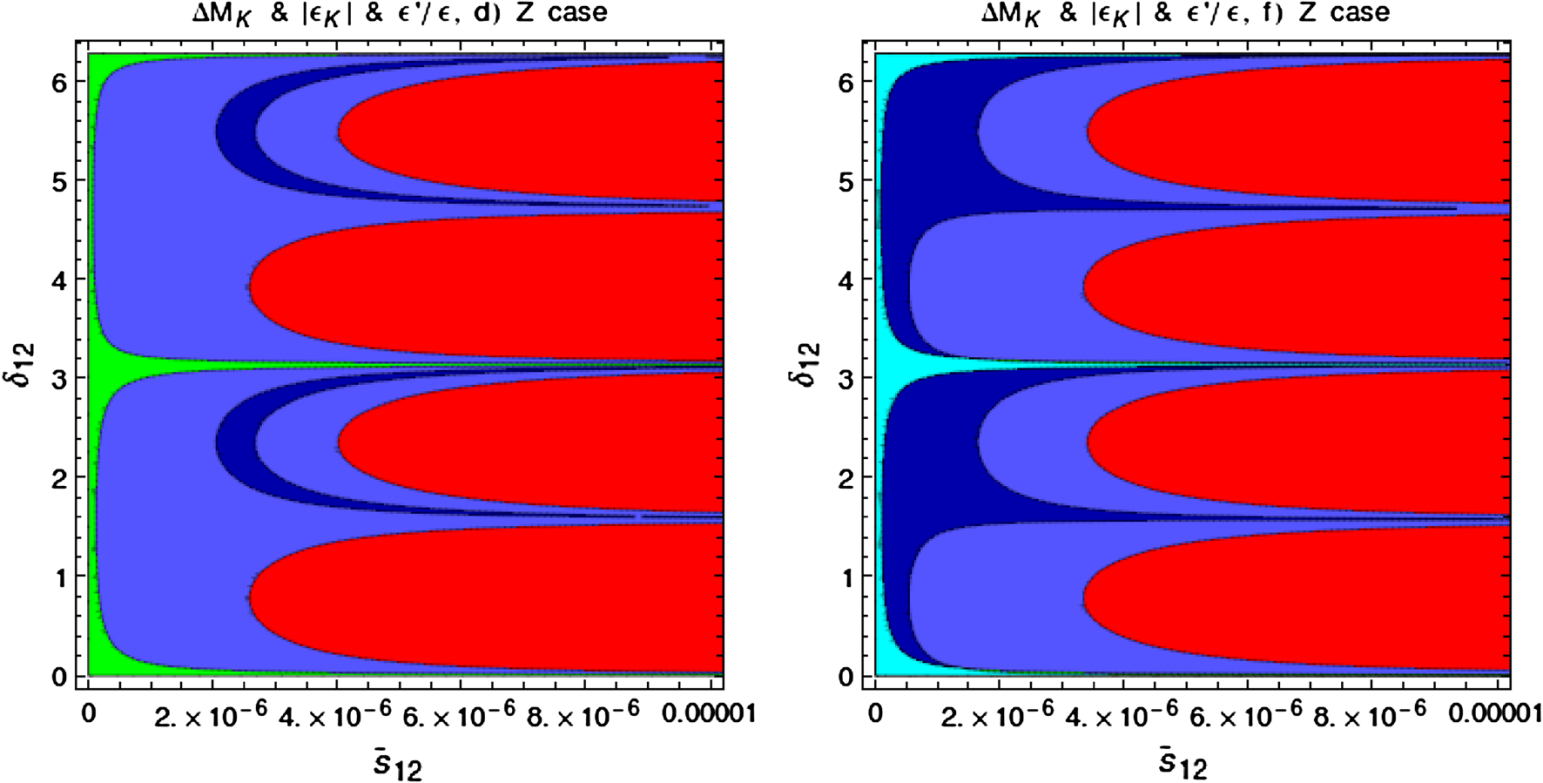
As in Fig. 12 but for RHS

**Fig. 16:**
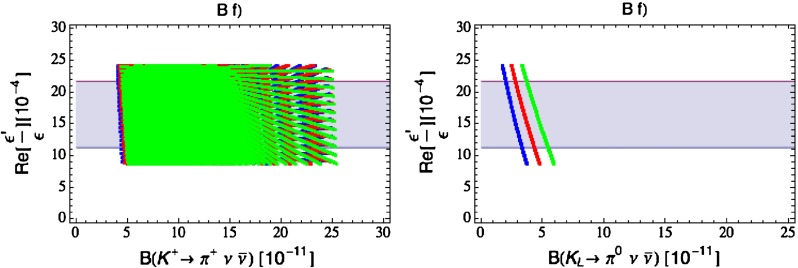
As in Fig. 13 but for RHS
- The constraint from $K_L\rightarrow \mu ^+\mu ^-$ is weaker, allowing for a larger enhancements of $\mathcal {B}(K^+\rightarrow \pi ^+\nu \bar{\nu })$.

**Fig. 17:**
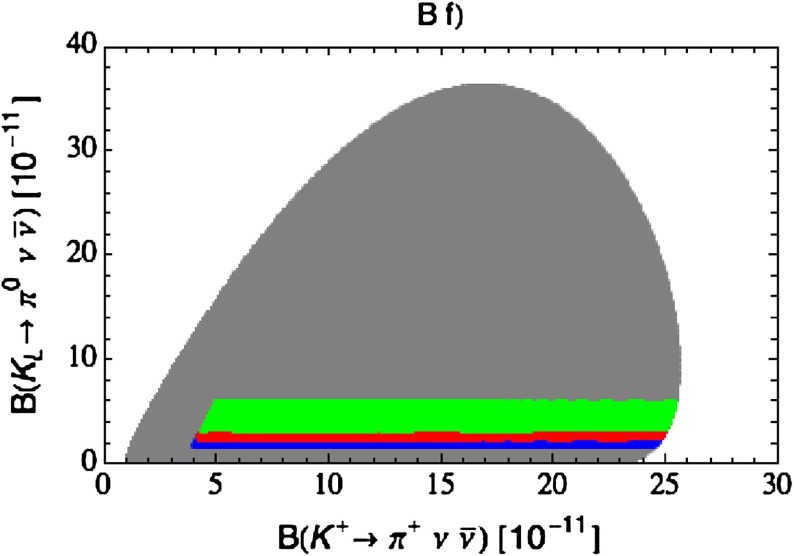
$\mathcal {B}(K_{L}\rightarrow \pi ^0\nu \bar{\nu })$ versus $\mathcal {B}(K^+\rightarrow \pi ^+\nu \bar{\nu })$ for scenario $(f)$ as in Fig. 14 but for RHS

Repeating the analysis performed in the LHS scenario for the RHS scenario we find the results in Figs. 15, 16, 17. The main messages from these plots when compared with Figs. 12, 13, 14 are as follows:

- The constraint from $\varepsilon '/\varepsilon$ is stronger, not allowing enhancements of $\mathcal {B}(K_{L}\rightarrow \pi ^0\nu \bar{\nu })$ as large as in the LHS case,

These results are easy to understand. As already discussed in [26] the outcome for the allowed values of $\Delta ^{sd}_R(Z)$ following from $\Delta M_K$ and $\varepsilon _K$ is identical to the one for $\Delta ^{sd}_L(Z)$. This is confirmed in Fig. 15, which should be compared with Fig. 12. But the Wilson coefficient $C_8^\prime (m_c)$ is by a factor of 3 larger than $C_8(m_c)$ in the LHS case. The difference in sign of these two coefficients is compensated for by the one of the hadronic matrix elements so that simply the suppression of $\varepsilon '/\varepsilon$ through NP and the $\varepsilon '/\varepsilon$ constraint in Fig. 15 is by a factor of 3 stronger than in the LHS case in Fig. 12. On the other hand for a given value of $\Delta ^{sd}_R(Z)$ the branching ratios $\mathcal {B}(K_{L}\rightarrow \pi ^0\nu \bar{\nu })$ and $\mathcal {B}(K^+\rightarrow \pi ^+\nu \bar{\nu })$ are not modified. But the values of $\mathrm{Im}\Delta ^{sd}_R(Z)$ are now stronger bounded from above by $\varepsilon '/\varepsilon$ than in the LHS case, which implies a stronger upper bound on $\mathcal {B}(K_{L}\rightarrow \pi ^0\nu \bar{\nu })$, as is clearly seen in Fig. 16. While this also has an impact on $\mathcal {B}(K^+\rightarrow \pi ^+\nu \bar{\nu })$ on the branch where the two branching ratios are strongly correlated, on the second branch where $\mathrm{Re}\Delta ^{sd}_R(Z)$ matters, the weaker constraint from $K_L\rightarrow \mu ^+\mu ^-$ allows for larger enhancements of $\mathcal {B}(K^+\rightarrow \pi ^+\nu \bar{\nu })$ than in the LHS case. The difference in this pattern between the LHS and RHS scenarios is best seen when comparing Fig. 14 with Fig. 17.

### The LRS and ALRS scenarios

When both $\Delta _L^{sd}(Z)$ and $\Delta _R^{sd}(Z)$ are present the general formula for $\varepsilon '/\varepsilon$ is given as follows:

170 $$\begin{aligned} \left( \frac{\varepsilon '}{\varepsilon }\right) = \left( \frac{\varepsilon '}{\varepsilon }\right) _\text {SM}+ \left( \frac{\varepsilon '}{\varepsilon }\right) ^L_{Z}+ \left( \frac{\varepsilon '}{\varepsilon }\right) ^R_{Z} \end{aligned}$$

with the last two terms representing the LHS and RHS contributions discussed above. Imposing relations between $\Delta _L^{sd}(Z)$ and $\Delta _R^{sd}(Z)$, which characterise the LRS and ALRS scenarios, one can calculate $\varepsilon '/\varepsilon$ in these scenarios.

As far as rare decays are concerned in the LRS scenario, the NP contributions to $K_L\rightarrow \mu ^+\mu ^-$ vanish, which allows in principle for larger enhancement of $\mathcal {B}(K^+\rightarrow \pi ^+\nu \bar{\nu })$ than is possible in other scenarios. On the other hand for fixed values of $\Delta _L^{sd}(Z)=\Delta _R^{sd}(Z)$ the $\varepsilon '/\varepsilon$ constraint is by a factor of 4 larger than in the LHS case, because the operators $Q_8$ and $Q_8^\prime$ contribute to $\varepsilon '/\varepsilon$ with the same sign. Therefore it is evident that the NP effects in $\mathcal {B}(K_{L}\rightarrow \pi ^0\nu \bar{\nu })$ will be even smaller than in the RHS scenario.

**Fig. 18:**
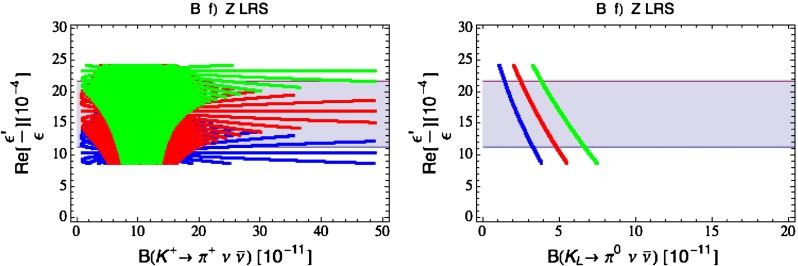
As in Fig. 13 but for LRS

**Fig. 19:**
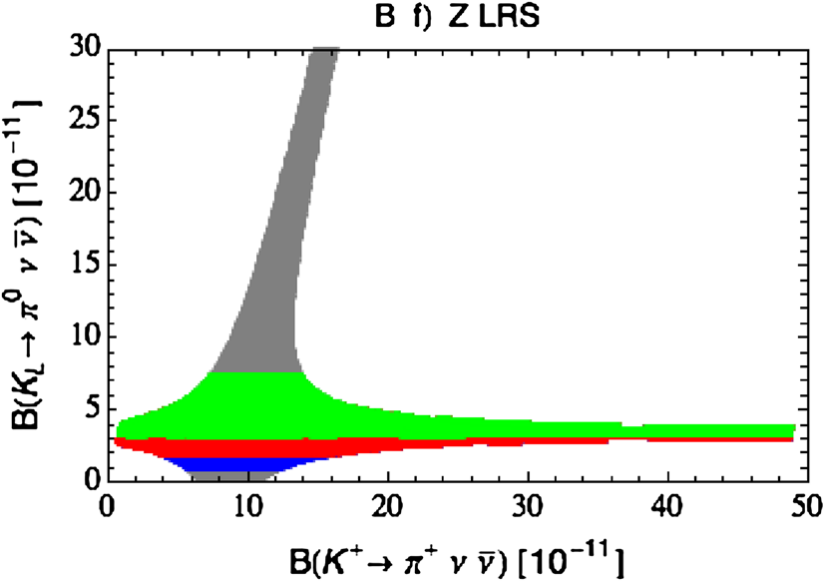
$\mathcal {B}(K_{L}\rightarrow \pi ^0\nu \bar{\nu })$ versus $\mathcal {B}(K^+\rightarrow \pi ^+\nu \bar{\nu })$ for scenario $(d)$ and $(f)$ as in Fig. 14 but for LRS

But now comes another effect which suppresses the NP contributions in $\mathcal {B}(K_{L}\rightarrow \pi ^0\nu \bar{\nu })$ even further. Indeed one should recall that in the LRS scenario the $\Delta S=2$ analysis is more involved than in the LHS and RHS scenarios because of the presence of LR operators which, as we have seen, in scenario A for the $Z^\prime$ play an essential role in allowing one to satisfy the constraints from $\Delta M_K$ and $\mathrm{Re} A_0$. But in the case at hand the constraints from $\Delta M_K$ and $\varepsilon _K$ imply simply much smaller allowed values of $\Delta _L^{sd}(Z)=\Delta _R^{sd}(Z)$ and in turn smaller NP effects in the branching ratios $\mathcal {B}(K_{L}\rightarrow \pi ^0\nu \bar{\nu })$ and $\mathcal {B}(K^+\rightarrow \pi ^+\nu \bar{\nu })$. This is partially compensated by the fact that now for fixed $\Delta _L^{sd}(Z)=\Delta _R^{sd}(Z)$ the NP contributions to the amplitudes for $K_{L}\rightarrow \pi ^0\nu \bar{\nu }$ and $K^+\rightarrow \pi ^+\nu \bar{\nu }$ are enhanced by a factor of 2 and in the case of $K^+\rightarrow \pi ^+\nu \bar{\nu }$ by the absence of the $K_L\rightarrow \mu ^+\mu$ constraint. The final result of this competition is shown in Figs. 18 and 19. In particular $\mathcal {B}(K^+\rightarrow \pi ^+\nu \bar{\nu })$ can be very much enhanced. Comparison of Figs. 14 (LHS), 17 (RHS) and 19 (LRS) could one day allow us to distinguish between these three scenarios, provided deviations from the SM predictions will be sizable.

In the ALRS scenario the NP contributions to $K^+\rightarrow \pi ^+\nu \bar{\nu }$ and $K_{L}\rightarrow \pi ^0\nu \bar{\nu }$ vanish but $\varepsilon '/\varepsilon$ is modified. For the same values of $\Delta _R^{sd}(Z)=-\Delta _L^{sd}(Z)$ the NP effect in $\varepsilon '/\varepsilon$ is only by a factor of 2 larger than in the LHS scenario because the contribution of $Q_8^\prime$ operator to $\varepsilon '/\varepsilon$ is partially cancelled by the one of $Q_8$. Moreover, as in the LRS scenario the values of the coupling $\Delta _R^{sd}(Z)=-\Delta _L^{sd}(Z)$ must be reduced in order to satisfy the $\Delta M_K$ and $\varepsilon _K$ constraints. But on the whole the results do not look interesting and we refrain from showing any plots.

## Summary and conclusions

In the present paper we had two main goals:

- to investigate whether a subleading part of the $\Delta I=1/2$ rule, at the level of $20$–$30\,\%$, could be due to NP contributions originating in tree-level FCNC transitions mediated by a heavy colourless gauge boson $Z^\prime$ or an $SU(3)_c$ colour octet of gauge bosons $G^\prime$,
- to extend our previous analysis of tree-level $Z^\prime$ and $Z$ FCNCs in [26] to the ratio $\varepsilon '/\varepsilon$ and as a byproduct to update the SM analysis of this ratio. This was in particular motivated by the rather precise value of $B_8^{(3/2)}$ obtained from QCD lattice calculations [21] that governs the electroweak penguin contributions to $\varepsilon '/\varepsilon$.

As the experimental value for the smaller amplitude $\mathrm{Re}A_2$ has been successfully explained within the SM, both within dual representation of QCD as a theory of weakly interacting mesons [17] and by QCD lattice calculations [18–21] we concentrated our analysis in the context of the first goal on the large amplitude $\mathrm{Re}A_0$, which is by a factor of 22 larger than $\mathrm{Re}A_2$ and its experimental value is not fully explained in these two approaches. In order to protect $\mathrm{Re}A_2$ from modifications we searched for NP that would have the property of the usual QCD-penguins. They are capable of shifting upwards $\mathrm{Re}A_0$ by an amount that at scales $\mathcal {O}(1\, \mathrm{GeV})$ is roughly by a factor of 3 larger than $\mathrm{Re}A_2$ without producing any relevant modification in the latter amplitude up to small isospin breaking effects.

**Fig. 20:**
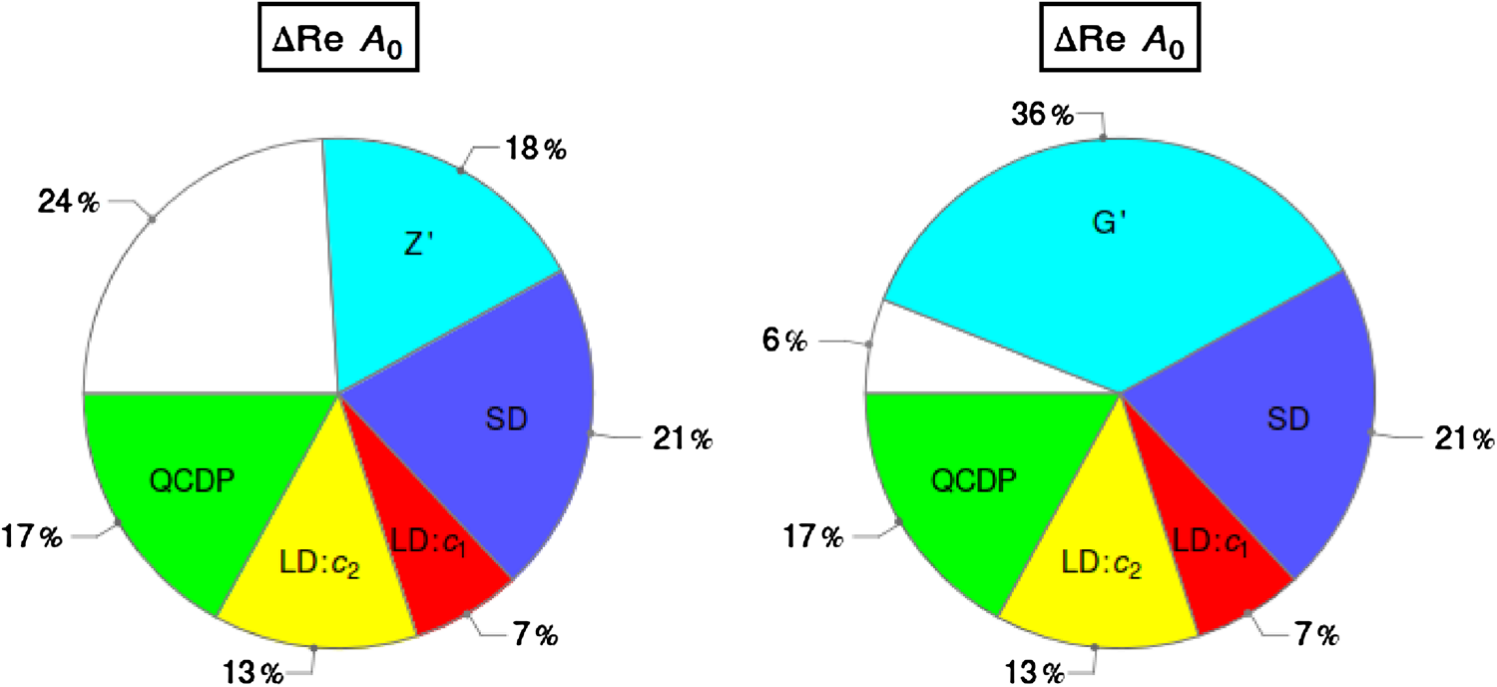
Budgets of different enhancements of $\mathrm{Re}A_0$, denoted here by $\Delta \mathrm{Re}A_0$. $Z^\prime$ and $G^\prime$ denote the contributions calculated in the present paper. The remaining coloured contributions come from the SM dynamics as calculated in [17]. The *white region* stands for the missing piece

However, due to GIM mechanism the QCD-penguin contribution within the SM is not large enough to allow one within the dual approach to QCD to fully reproduce the experimental value of $\mathrm{Re}A_0$ [17]. Therefore we searched for a QCD-penguin like contribution that is not GIM suppressed. As we have demonstrated in the present paper, a neutral heavy gauge boson with FCNCs (with or without colour) and approximately flavour universal right-handed diagonal couplings to quarks is capable of providing an additional upward shift in $\mathrm{Re}A_0$ while satisfying the constraints from $\varepsilon _K$, $\Delta M_K$, $\varepsilon '/\varepsilon$ and the LHC. Even if the structure of the relevant couplings must have a special hierarchy, summarised in (7), (84) and (133), we find this result interesting. Indeed our toy models for $Z^\prime$ and $G^\prime$ together with the dominant SM dynamics provide a better description of the $\Delta I=1/2$ rule that it is presently possibly within the SM so that in these NP scenarios we find that the values

171 $$\begin{aligned} R=\frac{\mathrm{Re}A_0}{\mathrm{Re}A_2}\approx 18~(Z^\prime ), R=\frac{\mathrm{Re}A_0}{\mathrm{Re}A_2}\approx 21~(G^\prime ) \end{aligned}$$

can be obtained. This is fully compatible with the experimental value in (2), even if in the case of $Z^\prime$ this ratio is visibly below the data. These results are summarised in Fig. 20 where also the budget of different SM contributions calculated in [17] is shown.

We identified a *quartic* correlation between the NP contributions to $\mathrm{Re}A_0$, $\varepsilon '/\varepsilon$, $\Delta M_K$ and $\varepsilon _K$, which offers means for a more precise determination of the required properties of the neutral gauge bosons in question. Moreover, in order to stay within the perturbative regime for the couplings involved and explain the $\Delta I=1/2$ rule, $M_{Z^\prime }$ in scenario A has to be at most a few TeV so that these simple extensions of the SM can be tested through the upgraded LHC and rare decays in the flavour precision era.

As our first goal, termed scenario A, led to a fine-tuned scenario that could be ruled out one day, as a plan B, we have considered scenario B for both tree-level heavy neutral gauge boson exchanges and $Z$ boson exchanges ignoring the $\Delta I=1/2$ rule constraint and concentrating on $\varepsilon '/\varepsilon$ and its correlation with branching ratios for rare decays $K^+\rightarrow \pi ^+\nu \bar{\nu }$ and $K_{L}\rightarrow \pi ^0\nu \bar{\nu }$. In this scenario $M_{Z^\prime }$ can be well above the LHC range and its increase can be compensated for by the increase of $Z^\prime$ couplings still fully within the perturbative regime.

The most important findings of our paper are as follows:

- Within models containing only left-handed or only right-handed flavour-violating $Z^\prime$ or $G^\prime$ couplings to quarks it is impossible to generate any relevant contribution to $\mathrm{Re}A_0$ without violating the constraint from $\Delta M_K$. The same applies to models with left-handed and right-handed couplings being equal or differing by sign.
- On the other hand $Z^\prime$ having in addition to $\Delta _L^{sd}(Z^\prime )=\mathcal {O}(1)$, a small right-handed coupling $\Delta _R^{sd}(Z^\prime )=\mathcal {O}(10^{-3})$ and $M_{Z^\prime }$ in the reach of the LHC can improve the present status of $\Delta I=1/2$ rule, as summarised in (171), provided the diagonal coupling $\Delta _R^{qq}(Z^\prime )=\mathcal {O}(1)$. As demonstrated in [82] and shown in Figs. 3 and 9 such couplings are still allowed by the LHC data. As seen in (171) even larger values of $R$ can be obtained in $G^\prime$ scenario.
- As far as $\varepsilon '/\varepsilon$ is concerned, the interesting feature of this NP scenario is the absence of NP contributions to the electroweak penguin part of this ratio, a feature rather uncommon in many extensions of the SM. NP enters here only through QCD-penguins and this implies interesting correlation between the new dynamics in $\varepsilon '/\varepsilon$ and the $\Delta I=1/2$ rule. In particular, we have identified an interesting correlation between the NP contributions to $\mathrm{Re}A_0$, $\varepsilon '/\varepsilon$, $\varepsilon _K$ and $\Delta M_K$, which is shown in Fig. 4 for two sets of CKM parameters, which among the six considered by us are the only ones that allow for simultaneous agreement for $\varepsilon '/\varepsilon$ and $\varepsilon _K$ and the significant contribution of $Z^\prime$ or $G^\prime$ to $\mathrm{Re}A_0$. This means that only for the inclusive determinations of $|V_{ub}|$ and $|V_{cb}|$ these heavy gauge bosons have a chance to contribute in a significant manner to the $\Delta I=1/2$ rule. This assumes the absence of other mechanisms at work, which would help in this case if the exclusive determinations of these CKM parameters would turn out to be true.
- Interestingly, in scenario A for $Z^\prime$ NP contributions to the branching ratio for $K_{L}\rightarrow \pi ^0\nu \bar{\nu }$ are negligible when the experimental constraint for $K^+\rightarrow \pi ^+\nu \bar{\nu }$ is taken into account.
- As a byproduct we updated the values of $\varepsilon '/\varepsilon$ in the SM stressing various uncertainties, originating in the values of $|V_{ub}|$ and $|V_{cb}|$. In particular we have found that the best agreement of the SM with the data is obtained for $B_6^{(1/2)}\approx 1.0$, that is, close to the large $N$ limit of QCD.
- In the case of $Z^\prime$, in the context of scenario $B$, that is, ignoring the issue of the $\Delta I=1/2$ rule and concentrating on $Z^\prime$ with exclusively left-handed couplings, we have studied correlations between $\varepsilon '/\varepsilon$ and the branching ratios for rare decays $K^+\rightarrow \pi ^+\nu \bar{\nu }$ and $K_{L}\rightarrow \pi ^0\nu \bar{\nu }$. In particular, we have found that for $B_6^{(1/2)}=0.75$ for which the SM value of $\varepsilon '/\varepsilon$ is much lower than the data, the cure of this problem through a $Z^\prime$ implies very enhanced values of $\mathcal {B}(K_{L}\rightarrow \pi ^0\nu \bar{\nu })$. Simultaneously $\mathcal {B}(K^+\rightarrow \pi ^+\nu \bar{\nu })$ is uniquely enhanced so that a triple correlation between these three observables exists. Figures 6 and 7 show this in a transparent manner.
- We have also demonstrated that the SM $Z$ boson with FCNC couplings cannot provide the missing piece in $\mathrm{Re}A_0$ without violating the constraint from $\mathrm{Re}A_2$. Still the correlation between $\varepsilon '/\varepsilon$, $K^+\rightarrow \pi ^+\nu \bar{\nu }$ and $K_{L}\rightarrow \pi ^0\nu \bar{\nu }$ can be used to test this NP scenario as demonstrated in Figs. 13 and 14. In particular very large enhancements of $\mathcal {B}(K_{L}\rightarrow \pi ^0\nu \bar{\nu })$ found by us in [26] are excluded when the constraint from $\varepsilon '/\varepsilon$ is taken into account: a property known from other studies.
- We have also investigated various scenarios for flavour-violating $Z$ couplings stressing different impact of $\varepsilon '/\varepsilon$ and $K_L\rightarrow \mu ^+\mu ^-$ constraints on rare branching ratios $\mathcal {B}(K^+\rightarrow \pi ^+\nu \bar{\nu })$ and $\mathcal {B}(K_{L}\rightarrow \pi ^0\nu \bar{\nu })$. In this context the comparison of Figs. 14 (LHS), 17 (RHS) and 19 (LRS) could one day allow us to distinguish between these three scenarios, provided the deviations from the SM predictions will be sizable.

In summary, a neutral $Z^\prime$ or $G^\prime$ with very special FCNC couplings summarised in (7) and the mass in the reach of the LHC could in principle be responsible for the missing piece in $\mathrm{Re}A_0$. Whether heavy gauge bosons with such properties exist should be answered by the LHC in this decade. In particular, a dedicated study of the dashed surface in Figs. 3 and 9 in the context of our simple models would be very interesting, as this would put the bounds used in our paper on a firm footing. This applies also to the bounds on the coupling $\Delta _L^{sd}(G^\prime )$ and the fact that the bounds obtained in [82] where derived under the condition that either $\Delta ^{sd}_L$ or $\Delta ^{qq}_R$ is vanishing. The presence of interferences between various contributions governed by these two couplings would not necessarily make the bounds on them stronger and could in fact soften them. Moreover, in the former case the version of our models in which the primed operator $Q_6^\prime$ is dominant could still provide the solution to the $\Delta I=1/2$ rule as discussed in Sect. 5.6.

If $Z^\prime$ or $G^\prime$ with such properties do not exist, it is likely that the $\Delta I=1/2$ rule follows entirely from the SM dynamics. Confirmation of this from lattice QCD would be in this case important. On the other hand any $Z^\prime$ with non-vanishing flavour-violating couplings to quarks can have impact on $\varepsilon '/\varepsilon$, $K^+\rightarrow \pi ^+\nu \bar{\nu }$ and $K_{L}\rightarrow \pi ^0\nu \bar{\nu }$ and the correlations between them. This also applies to scenario with flavour-violating $Z$ couplings. In both cases the numerous plots presented by us should help in monitoring the exciting events to be expected at the LHC and in flavour physics in the second half of this decade.

## Footnotes

1 The SM contributions are evaluated including NLO QCD corrections.

2 The reason for choosing $\mu =m_c$ will be explained below.

3 We thank Matthias Jamin for providing this table for the most recent values of $\alpha _s(M_Z)$.

**Table 1:** The coefficients $r_i^{(0)}$, $r_i^{(6)}$ and $r_i^{(8)}$ of formula (55) in the NDR scheme for three values of $\alpha _s(M_Z)$

$i$	$\alpha _s(M_Z)= 0.1179$			$\alpha _s(M_Z)= 0.1185$			$\alpha _s(M_Z)= 0.1191$		
	$r_i^{(0)}$	$r_i^{(6)}$	$r_i^{(8)}$	$r_i^{(0)}$	$r_i^{(6)}$	$r_i^{(8)}$	$r_i^{(0)}$	$r_i^{(6)}$	$r_i^{(8)}$
0	–3.572	16.424	1.818	–3.580	16.801	1.782	–3.588	17.192	1.744
$X_0$	0.575	0.029	0	0.572	0.030	0	0.569	0.031	0
$Y_0$	0.405	0.119	0	0.401	0.121	0	0.398	0.123	0
$Z_0$	0.709	–0.022	–12.447	0.724	–0.023	–12.631	0.739	–0.023	–12.822
$E_0$	0.215	–1.898	0.546	0.211	–1.929	0.557	0.208	–1.961	0.568

4 As mentioned in Sect. 5.2 the complete exclusion of the grey area would require a more intensive study of points corresponding to larger values of $\Delta _R(Z^\prime )$ and $M_{Z^\prime }< 3\, \mathrm{TeV}$.

5 A.J.B. would like to thank Bogdan Dobrescu, Maikel de Vries and Andreas Weiler for discussions on this issue.

6 We prefer to quote for the central value of $|V_{cb}|$ the most recent value from [74] rather than the one given in Table 2.

7 The details of this analysis will be presented elsewhere.

8 The case of $\Delta _R^{qq}(G^\prime )=1.0$ and $M_{G^\prime }=3.0\, \mathrm{TeV}$ is ruled out by dijet data from CMS and direct comparison with $Z^\prime$ for these parameters is not possible.
